# Cell-Free Gene Expression Dynamics in Synthetic Cell Populations

**DOI:** 10.1021/acssynbio.1c00376

**Published:** 2022-01-04

**Authors:** David T. Gonzales, Naresh Yandrapalli, Tom Robinson, Christoph Zechner, T-Y. Dora Tang

**Affiliations:** † Max Planck Institute of Molecular Cell Biology and Genetics, 01307 Dresden, Germany; ‡ Center for Systems Biology Dresden, 01307 Dresden, Germany; § Max Planck Institute of Colloids and Interfaces, 14476 Potsdam, Germany; ∥ Physics of Life, Cluster of Excellence, TU Dresden, 01603 Dresden, Germany

## Abstract

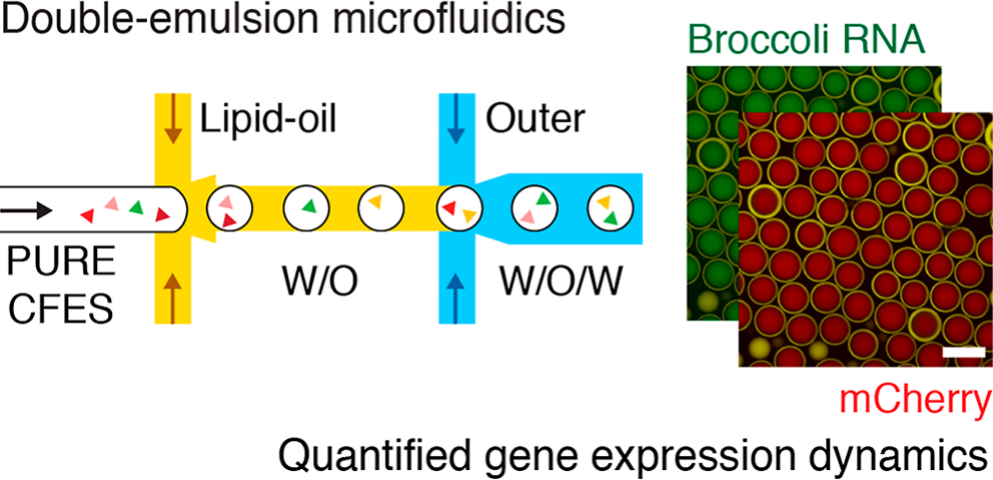

The ability to build synthetic cellular populations from the bottom-up provides the groundwork to realize minimal living tissues comprising single cells which can communicate and bridge scales into multicellular systems. Engineered systems made of synthetic micron-sized compartments and integrated reaction networks coupled with mathematical modeling can facilitate the design and construction of complex and multiscale chemical systems from the bottom-up. Toward this goal, we generated populations of monodisperse liposomes encapsulating cell-free expression systems (CFESs) using double-emulsion microfluidics and quantified transcription and translation dynamics within individual synthetic cells of the population using a fluorescent Broccoli RNA aptamer and mCherry protein reporter. CFE dynamics in bulk reactions were used to test different coarse-grained resource-limited gene expression models using model selection to obtain transcription and translation rate parameters by likelihood-based parameter estimation. The selected model was then applied to quantify cell-free gene expression dynamics in populations of synthetic cells. In combination, our experimental and theoretical approaches provide a statistically robust analysis of CFE dynamics in bulk and monodisperse synthetic cell populations. We demonstrate that compartmentalization of CFESs leads to different transcription and translation rates compared to bulk CFE and show that this is due to the semipermeable lipid membrane that allows the exchange of materials between the synthetic cells and the external environment.

## 1 Introduction

The establishment of synthetic multicellular systems that can sustain out-of-equilibrium behavior is a major challenge in bottom-up synthetic biology. This requires the integration of chemical reaction networks for intercellular communication between synthetic cells within populations. Recent examples of bottom-up multicellular systems demonstrate how simple chemical building blocks can be used to make functional systems that scale from molecules to cells to synthetic cell communities^1,2^ or tissues.^3,4^ This provides a route for characterizing how cell–cell communication could lead to collective behavior in a minimal and multicellular context. Importantly, relative to biological systems, bottom-up synthetic cells and cell populations are highly amenable to perturbations and modifications for experiments and quantitative analysis. However, transitioning from single synthetic cells to multicellular systems is challenging because of our poor ability to control molecular assembly beyond a certain number of modules. Highly engineered systems and controllable methodologies coupled with mathematical modeling can facilitate building more complex and multiscale chemical systems from the bottom-up. It has already been shown how models of cell-free expression systems (CFESs) can help unravel the modular response of highly complex systems responsible for supporting out-of-equilibrium behavior.^5^

CFESs encapsulated in lipid vesicles have been established as one of the most popular and utilized types of synthetic cells as they encompass both cellular compartmentalization and the central biological dogma of transcription and translation in a minimalistic fashion. These minimal synthetic cells have demonstrated simple transcription and translation pathways,^6^ gene expression cascades,^7^ gene expression bursting,^8^ noise from macromolecular crowding,^9^ stochastic gene expression,^10^ genetic circuits for intercellular communication,^11^ and CFESs coupled with other metabolic processes including ATP production^12^ and DNA replication,^13^ demonstrating their potential as a platform to build communicating populations of synthetic cells. Despite these successes, compartmentalized cell-free gene expression dynamics has not yet been fully quantified and modeled. This can provide a simplified system that focuses on the effect of compartmentalization on gene expression dynamics.

Even though CFESs are dramatically reduced in complexity compared to biological cells, they still contain as many as 37 enzymes and 32 small-molecule compounds or substrates in purified reconstituted cell-free systems^14,15^ and even more for crude extract-based CFES. This makes it challenging to collect sufficient data to test existing models or to develop tractable models that rely on the knowledge of precise chemical species as a function of time. Fortunately, there is an increasing effort toward proteomic and metabolic profiling of CFES reactions that have the potential to provide quantitative molecular analysis to give details on reaction dynamics and limits on resources.^16−19^ Alternatively, coarse-grained CFES models circumvent the need to measure all molecular species by focusing on only a few species such as DNA, RNA, proteins, RNA polymerases, and ribosomes. Several models have already been demonstrated to faithfully capture quantified cell-free gene expression dynamics in bulk solutions. For instance, several coarse-grained models have used first-order and Michaelis–Menten kinetics to describe cell-free transcription and translation dynamics^20−22^ and extended to explicitly account for RNA polymerase and ribosome species.^23,24^ Dynamic models that include the initiation, elongation, and termination steps of translation^25,26^ or central carbon metabolism^27^ have been used to identify bottlenecks in transcription and translation, which can be experimentally relieved to improve protein productivity in CFESs. These examples demonstrate how quantitative coarse-grained models can provide a better understanding of the CFES building blocks and will be crucial for further engineering of more complex synthetic multicellular systems.

As studies have shown differences in protein production by mammalian cell-free expression within water–oil emulsions,^28^ quantifying and modeling compartmentalized cell-free gene expression could provide insights into the effect of encapsulation on gene expression. Methodologies to monitor mRNA and protein dynamics in bulk and semipermeable liposomes using cell-free systems have been demonstrated by utilizing fluorescence resonance energy transfer (FRET) donor–acceptor pairs for mRNA,^22,29^ fluorescent proteins such as GFP and YFP,^30,31^ and fluorescent Spinach RNA aptamers simultaneously with YFP^32,33^ or mCherry.^9^ However, expressed mRNA and protein levels in encapsulated CFESs have only been measured as either relative fluorescence units within the synthetic cells or quantified concentrations for mRNA or protein levels separately. To the best of our knowledge, an absolute and simultaneous quantification of both transcription and translation dynamics within liposome synthetic cell populations has not yet been presented. This is crucial for models of compartmentalized cell-free gene expression to provide a better understanding of the effects of physical processes and environments, such as trans-membrane diffusion and surface effects, on gene expression dynamics and provides the basis to model increasingly complex compartmentalized cell-free gene expression circuits. Notably, studies on the effects of liposome compartmentalization to CFESs has revealed that optimization of the outer solution and liposome permeability can increase expression yield and prolong gene expression activity;^6^ CFESs encapsulated in small cell-sized volumes can result in stochastic gene expression^10^ and rare favorable phenotypes such as high expression,^30^ and gene expression resources and macromolecular crowding can affect gene expression bursting.^8,9^

Liposome-encapsulated transcription and translation machinery can be generated by lipid swelling^30,35^ or phase transfer methods.^8,10,33,36^ These methods result in synthetic cells with large variations in cell size and gene expression profiles.^8,9,30,31^ Bulk methods can be advantageous due to their accessibility and opportunity in exploring a large random space in terms of encapsulation and size,^30^ and populations of vesicles can be generated without specialized equipment. However, it can also be advantageous to generate uniform populations of synthetic cells for reproducibility and predictability. This can be achieved by leveraging recent developments in microfluidics^37−39^ and droplet printing technologies^40^ to generate populations of synthetic cells with greater throughput, control, and uniformity compared to standard bulk methods.^41,42^ Using microfluidic-generated synthetic cells has already been shown to be effective in generating monodisperse synthetic cells to study the effect of macromolecular crowding on gene expression without the use of synthetic crowding agents^43^ and to qualitatively monitor Spinach2 RNA aptamer transcription dynamics.^44^ This is especially important for quantitative approaches, as it enables the generation of statistically robust data that is amenable to accurate modeling.

In this study, we monitored and quantified both transcription and translation dynamics in bulk and liposome-encapsulated CFES reactions expressing Broccoli RNA aptamer and mCherry protein reporters. Using double-emulsion microfluidics,^38^ we generated monodisperse populations of synthetic cells encapsulating cell-free gene expression systems. Fluorescent readings were converted into absolute concentration units by a standard calibration curve to obtain a simultaneous and quantitative characterization of transcription and translation. Bulk reaction results were used to develop and select from several models of resource-limited cell-free gene expression.^22^ These models were compared to each other using the Akaike information criterion (AIC), and profile likelihood analysis was performed to quantify the kinetic parameters and their uncertainties. The best-ranking model was then used to compare rate parameters of gene expression dynamics between bulk experiments and synthetic cell populations. Overall, our work combines bottom-up assembly with robust modeling to provide a quantitative outlook of liposome-compartmentalized gene expression dynamics in synthetic cell populations. This has facilitated direct comparisons between experiments of bulk and compartmentalized CFES reactions and provides the basis for the design and construction of multicellular systems with increased levels of complexity.

## 2 Results and Discussion

### 2.1 Monitoring Transcription and Translation Dynamics in Cell-Free Expression Systems

**Figure 1:**
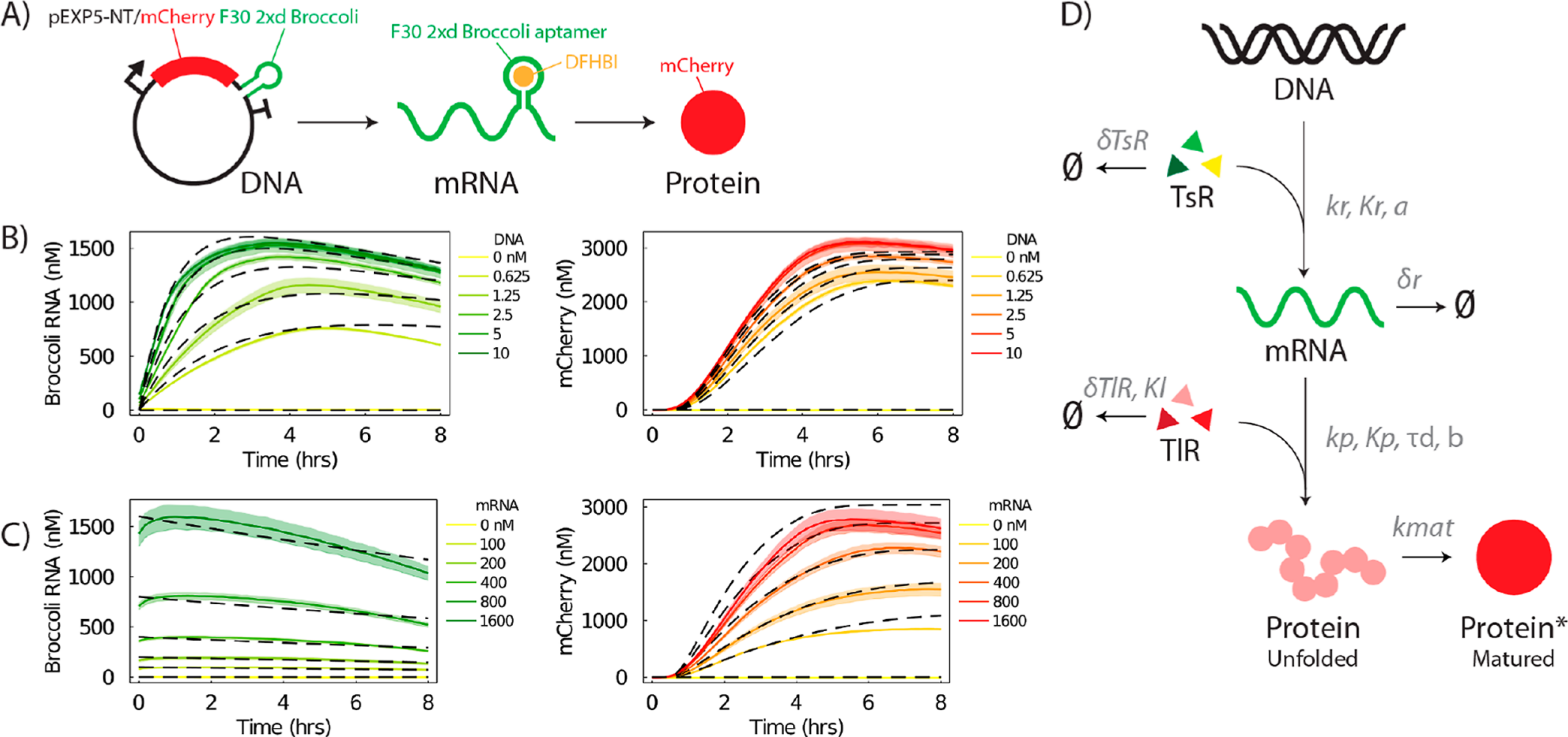
Monitoring transcription and translation dynamics in cell-free expression. (A) Construct of the pEXP5-NT/6xHis mCherry F30-2xdBroccoli plasmid containing a constitutive T7 RNAP-mediated promoter expressing 6xHis mCherry with an F30-2xdBroccoli RNA aptamer tag. The small-molecule dye DFHBI becomes fluorescent upon binding with the Broccoli RNA aptamer. (B) mRNA and mCherry protein expression levels over time from bulk PURExpress CFES titrated with varying concentrations of pEXP5-NT/6xHis mCherry F30-2xdBroccoli DNA plasmid. (C) mRNA and mCherry protein expression levels over time from bulk PURExpress CFESs titrated with varying concentrations of purified 6xHis mCherry F30-2xdBroccoli RNA transcripts. Solid lines and shaded areas correspond to mean and standard deviation values from triplicate experiments, respectively. Dashed lines are resource-limited CFES model fits. (D) Illustration of the resource-limited gene expression model for CFESs. Parameters are *k*_r_: RNA transcription rate, *K*_r_: dissociation constant between RNAP and DNA, δ_r_: RNA degradation rate, *k*_p_: protein translation rate, *K*_p_: dissociation constant between ribosome and RNA, *k*_mat_: mCherry maturation rate, δ_TsR_: TsR degradation rate, δ_TlR_: TlR degradation rate, *K*_l_: Michaelis–Menten constant for TlR degradation, *a*: scaling factor for consumption of TsR with transcription, *b*: scaling factor for consumption of TlR with translation, and τ_d_: time delay for protein translation.

To monitor both transcription and translation dynamics in CFESs, we constructed the pEXP5-NT/6xHis mCherry F30-2xdBroccoli plasmid. This plasmid consists of a constitutive T7 RNA polymerase-mediated promoter to express a red fluorescent mCherry protein and two copies of a dimeric Broccoli RNA aptamer stabilized by the F30 stem-loop^45,46^ between the stop codon of mCherry and the terminator of the gene construct (Figure 1A). Binding of a small-molecule dye, 3,5-difluoro-4-hydroxybenzylidene imidazolinone (DFHBI), to the Broccoli RNA aptamer results in a green fluorescence signal. This allows simultaneous fluorescence monitoring of transcribed mRNA and reporter protein levels. PURExpress CFES reactions were titrated with pEXP5-NT/6xHis mCherry F30-2xdBroccoli plasmid DNA or purified mRNA transcripts from the same plasmid. Reaction mixtures were incubated at 30 °C and monitored for mRNA and protein levels over time in a fluorescence well plate reader (Figure 1B,C). Relative fluorescence units were converted into nM concentration units using calibration curves from serial dilutions of Broccoli RNA aptamer and mCherry protein in the same reaction mix composition and acquisition settings (Section 5 in the Supporting Information). We observed typical profiles of gene expression in CFESs, where a signal is first detected from transcription of mRNA followed by translation of the mCherry protein. The gene expression profiles show a plateau at ∼3 h for mRNA and ∼5 h for protein. Rates of transcription reach a maximum at the initial point, while translation rates peak at 1.5–2 h (Figure S14 in the Supporting Information) and then gradually decrease. Our results also show that the endpoint protein concentrations increase with increasing plasmid DNA or mRNA transcript concentrations until a saturation concentration of 3.75 nM for plasmid DNA and 600 nM for mRNA transcript (Figure 1B,C). This indicates that gene expression rates and yield are dependent on both the consumption and degradation of resources. If gene expression was dependent solely on the consumption of resources, the final protein production would be constant regardless of DNA or mRNA input. This hypothesis is supported by previous work where the addition of ribosomes to PURExpress after exhaustion restores gene expression activity^22^ and a delayed addition of a DNA template into PURExpress after incubation results in reduced rates and yield of gene expression.^22,26^

### 2.2 Resource-Limited Gene Expression Model for Cell-Free Expression

To describe the dynamics of cell-free gene expression, a coarse-grained model based on Stögbauer et al. (2012)^22^ was developed to quantitatively compare results across experiments and literature values. This model accounts for both transcription and translation dynamics driven by a limited pool of resources for gene expression. Transcription (TsR) and translation resources (TlR) are assigned unitless quantities initialized at 1 and then gradually decreased to 0 as they are consumed by transcription or translation and degradation. These species serve as a phenomenological proxy to account for the cumulative effect of different limiting factors that fuel transcription and translation processes, such as RNA polymerase, ribosome concentrations, NTP, amino acids, and other energy resources. Based on this model, we generated several candidate CFES models composed of a system of delay and ordinary differential equations. We used our bulk experimental results from both transcription and translation dynamics and mCherry maturation experiments (Section 7 in the Supporting Information), to guide model selection. Candidate models were ranked among each other using the AIC.^47^ Profile likelihoods were then used to determine the parameter identifiability and confidence intervals (CIs) for each of the candidate models.^48,49^ The best-scoring model resulting from this analysis is shown in eqs 1–6 (Section 9 in the Supporting Information) below.
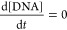
1
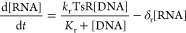
2
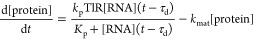
3
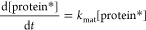
4
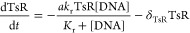
5
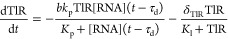
6

This model uses Michaelis–Menten-type kinetics for transcription and translation. Translation is additionally modeled by a delay differential equation with a time delay (τ_d_) to account for the time delay of protein expression observed in our mRNA titration experiments. Transcription and translation resources (TsR and TlR, respectively) are consumed by transcription and translation processes and degraded independently. These are consumed during transcription and translation with a scaling factor, *a* and *b*, respectively. Both resources, also, spontaneously degrade with first-order and Michaelis–Menten kinetics for TsR and TlR, respectively. RNA degradation and mCherry protein maturation are assigned first-order reactions (see model derivation in Section 8 of the Supporting Information). In contrast to the previously published model,^22^ TsR degradation was included to account for the independent exhaustion of transcription resources. Lastly, we included a time lag (τ_l_) in the fitting procedure of the model to account for the time between starting the CFES reaction and acquiring the first data point (Section 9 in the Supporting Information). This was negligible for our bulk experiments that took less than 10 min from adding the DNA or RNA template into the CFE bulk reactions to acquiring the first data points in the plate reader. However, it was important for the encapsulated experiments which had a lag period between sample preparation and measurements of ∼30 min. The model fit and optimized rate parameters and CIs are shown in Figure 1B,C (dashed lines) and Table 1. All parameters except for *K*_l_ and τ_l_ are well-identifiable. The estimates of *K*_l_ and τ_l_ turn out to be very low, and varying these parameters within one order of magnitude does not significantly affect the model fit. Overall, the model captures the general behavior of gene expression dynamics across different initial DNA and RNA conditions. The remaining quantitative mismatch is likely due to additional chemical complexity not captured by our coarse-grained model. The mCherry maturation rate parameter *k*_mat_ was determined independently using a protein maturation assay^50^ at 2.15 ± 0.12 h^–1^ (Section 7 in the Supporting Information). This corresponds to a maturation half-time (*t*_0.5_) of 19.31 ± 2.24 min, which is also comparable to previous reports of mCherry maturation at 15 min in *Escherichia coli*.^51^ Assuming that a standard PURExpress reaction contains 100 nM T7 RNA polymerase^14^ and 2.4 μM ribosomes (NEB), T7 RNA polymerase transcription and ribosome translation rates are approximately 8.2–11.1 NTP/s and 0.20–0.28 amino acid/s, respectively (calculated from *k*_r_ = 2728–3674 nM/h for a 1087 bp transcript and *k*_p_ = 2211–3108 nM/h for a 777 aa protein in Table 1). These values are lower than the reported *in vivo* rates in *E. coli* bacterial cells (230 ± 20 NTP/s^52^ and 8-18 amino acid/s^53^). However, the polymerase transcription rates and ribosome translation rates are similar to previous work in PURExpress expressing GFP at 37 °C (2.2 NTP/s and 0.03 amino acid/s, respectively).^22^ Using a FRET sensor to measure RNA transcription in PURExpress, initial transcription rates from 10 nM DNA plasmid template were previously measured at ∼7 nM/min.^29^ This is also comparable to our initial transcription rate measurements at 15.9 nM/min for 10 nM DNA plasmid (Figure S14 in the Supporting Information). The differences could be attributed to different reaction conditions, T7 RNA polymerase concentrations, the encoding gene, and/or batch-to-batch variability of the expression system.

**Table 1:** Parameter Estimates and Likelihood-Based 95% CI from the Resource-Limited Gene Expression Model Fitting on Bulk DNA and RNA Titration Experiments (θ̂_Bulk_) and Synthetic Cell Population DNA Titration Experiments (θ̂_Cell_)^a^

parameter	description	θ̂_bulk_	95% CI	θ̂cell	95% CI	units
*k*_r_	RNA transcription rate	2894	2728 - 3674	1899	1631 - 3537	nM/h
*K*_r_	Dissociation constant between RNAP and DNA	3.67	2.89 - 5.68	8.86	6.97 - 18.66	nM
δ_r_	RNA degradation rate	0.0392	0.0361 - 0.0422	0.0081	0.00239 - 0.0143	1/h
*k*_p_	Protein translation rate	2568	2211 - 3108	1954	1617 - 2696	nM/h
*K*_p_	Dissociation constant between ribosome and RNA	703	530 - 1347	1319	819 - 2038	nM
*k*_mat_	mCherry maturation rate	2.15	(±0.12)	2.15	(±0.12)	1/h
δ_TsR_	TsR degradation rate	0.231	0.171 - 0.298	0.154	0.136 - 0.175	1/h
δ_TlR_	TlR degradation rate	0.0884	0.0441 - 0.1187	0.244	0.184 - 0.684	1/h
*K*_l_	Michaelis–Menten constant for TlR degradation	1.21 × 10^–6^	–∞ - +∞	0.232	–∞ - 0.713	
*A*	scaling factor for consumption of TsR with transcription	4.45 × 10^–4^	4.18 × 10^–4^ - 4.57 × 10^–4^	6.60 × 10^–4^	6.21 × 10^–4^ - 6.74 × 10^–4^	
*b*	scaling factor for consumption of TlR with translation	1.78 × 10^–4^	1.18 × 10^–4^ - 2.42 × 10^–4^	4.46 × 10^–13^	–∞ - +∞	
τ_d_	time delay for protein translation	0.433	0.254 - 0.560	0.0576	–∞ - 0.279	h
τ_l_	time lag between the reaction start and data collection	2.81 × 10^–9^	–∞ - +∞	0.457	0.342 - 0.535	h

a Parameters with CIs at −∞ and/or +∞ are non/weakly-identifiable within one order of magnitude from θ̂.

### 2.3 Production of Synthetic Cell Populations with Low Variability

**Figure 2:**
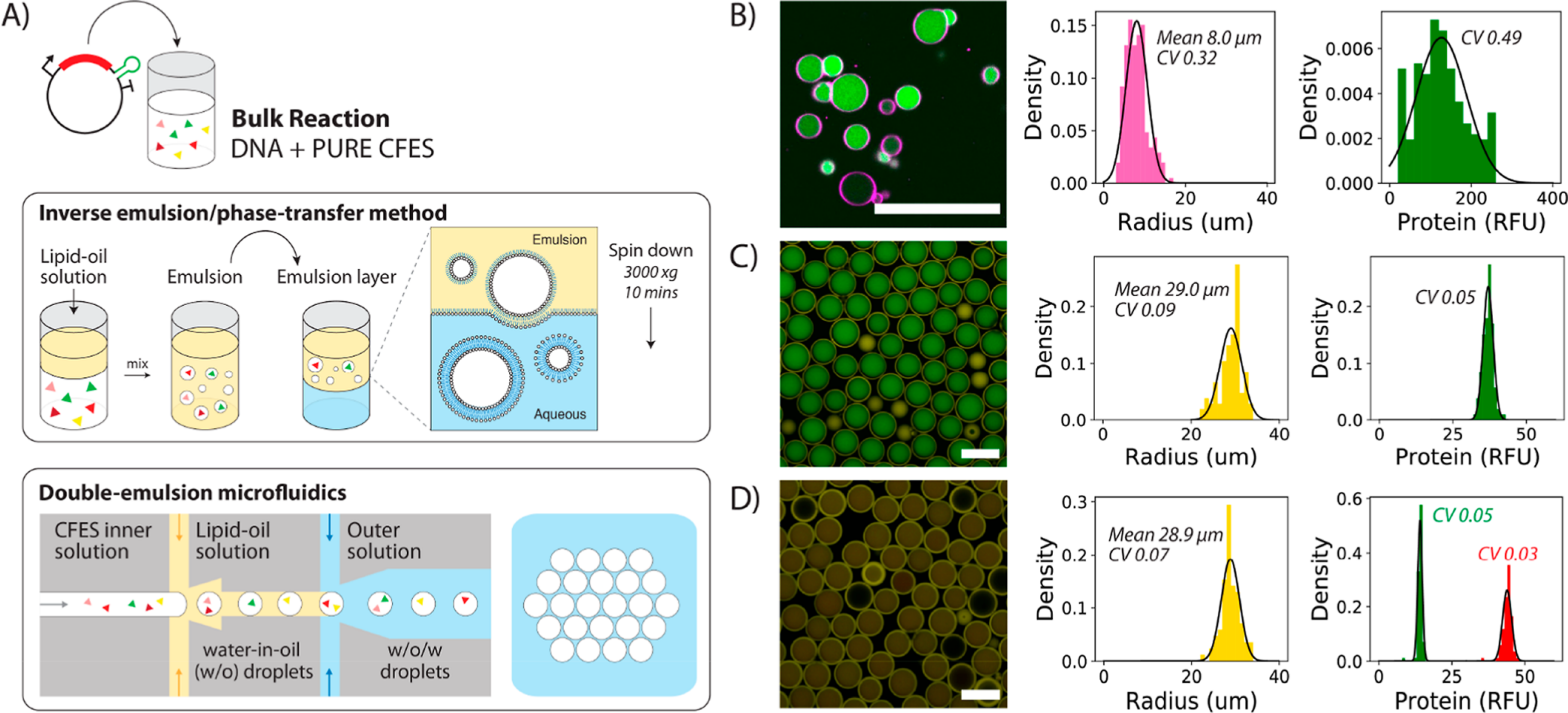
Variability in synthetic cell populations. (A) Schematic of the bulk inverse emulsion phase transfer method and double-emulsion microfluidics to generate liposomes or synthetic cells. (B) Synthetic cell population expressing eGFP protein from 1.17 nM pEXP5-NT/6xHis eGFP plasmid DNA generated using the bulk inverse emulsion phase transfer method. (C) Microfluidic-generated synthetic cells expressing eGFP protein from 4.5 nM pEXP5-NT/6xHis eGFP F30-2xdBroccoli plasmid DNA. (D) Merged image of the synthetic cell population expressing both eGFP and mCherry protein from two plasmids (4.5 nM pEXP5-NT/6xHis eGFP and 4.5 nM pEXP5-NT/6xHis mCherry plasmid DNA). Endpoint histograms of radius and protein RFU are plotted alongside each of the synthetic cell populations (B–D). The number of cells analyzed is 206, 106, and 85 for (B–D), respectively. Black lines are Gaussian distributions obtained by fitting mean and variance of the data. These experiments show the relative levels of expressed protein and do not refer to absolute concentrations. RFU values between the microfluidic-generated synthetic cells in (C,D), but not the inverse emulsion-made synthetic cells, are comparable, as these images were acquired using the same microscopy settings. However, CV values can be compared across all populations. All images are taken at the endpoint after 12 h of incubation at 30 °C using confocal microscopy with a 40× objective for (B) and 10× objective for (C,D). Scale bars are all 100 μm. Calibrated units of (C,D) are shown in Section 15 of the Supporting Information.

Having established a quantitative model for cell-free gene expression in bulk reactions, we next wanted to test its applicability on populations of compartmentalized reactions. To this end, we encapsulated PURExpress CFESs in lipid-based synthetic cell populations using either an inverse emulsion/phase transfer method^54^ or a double-emulsion microfluidic methodology^37,38^ (Figure 2A) (Materials and Methods and Sections 10–12 in the Supporting Information). The inner solution was composed of the PURExpress CFES and a plasmid DNA for constitutive T7 RNAP-mediated expression of a fluorescent protein gene (eGFP or mCherry). Confocal microscopy images for the inverted emulsion and microfluidic-generated synthetic cells were segmented to obtain relative fluorescence units (RFU) of the expressed protein in single cells in each population. These were then used to calculate the coefficient of variation (CV) of the distribution of the expressed protein in each cell population. The CV allows comparison of the variability of distributions with different scales of measurement. The inverted emulsion method generated liposomes with a mean radius of 8.0 μm and a CV of 0.32 (Figure 2B). In comparison, the microfluidic-generated synthetic cells were larger with a mean radius of 29.0 μm and exhibited lower size variation with a CV of 0.09 (Figure 2C) as expected. Protein expression in the inverted emulsion-generated synthetic cells also showed a greater variation (CV 0.49, Figure 2B) compared to synthetic cells produced in microfluidics (CV 0.05) (Figure 2C). These results are in agreement with previous studies of inverse emulsion-generated cells with expressed protein concentration CVs ranging from 0.20 to 0.80.^10^ We further show that simultaneous encapsulation of two plasmids in a microfluidic-generated synthetic cell population results in expression of both eGFP and mCherry proteins in each cell at a consistent ratio (3.11 ± 0.133 eGFP/mCherry RFU) (Figure 2D). This demonstrates the robustness of our synthetic cell production where the inner CFES solution is well-mixed and the microfluidic method maintains the homogeneity throughout encapsulation. The increased variance in phase transfer-generated cells is most likely due to a combined result of fluctuations in cell size and encapsulation. In contrast, the synthetic cell populations generated using double-emulsion microfluidics resulted in larger and more uniform cell populations, making them highly suitable for our quantitative analysis. In addition, it was also observed that the fluorescence from expression of the pEXP5-NT/6xHis eGFP plasmid is decreased in the two-plasmid synthetic cells (mean RFU 14.1, Figure 2D) compared to the single-plasmid synthetic cells (mean RFU 37.0, Figure 2C) (see also Section 15 in the Supporting Information). This is a result of gene expression resources being split between the expression of both eGFP and mCherry proteins in the two-plasmid synthetic cells.

### 2.4 Quantitative Transcription and Translation Dynamics in Synthetic Cell Populations

**Figure 3:**
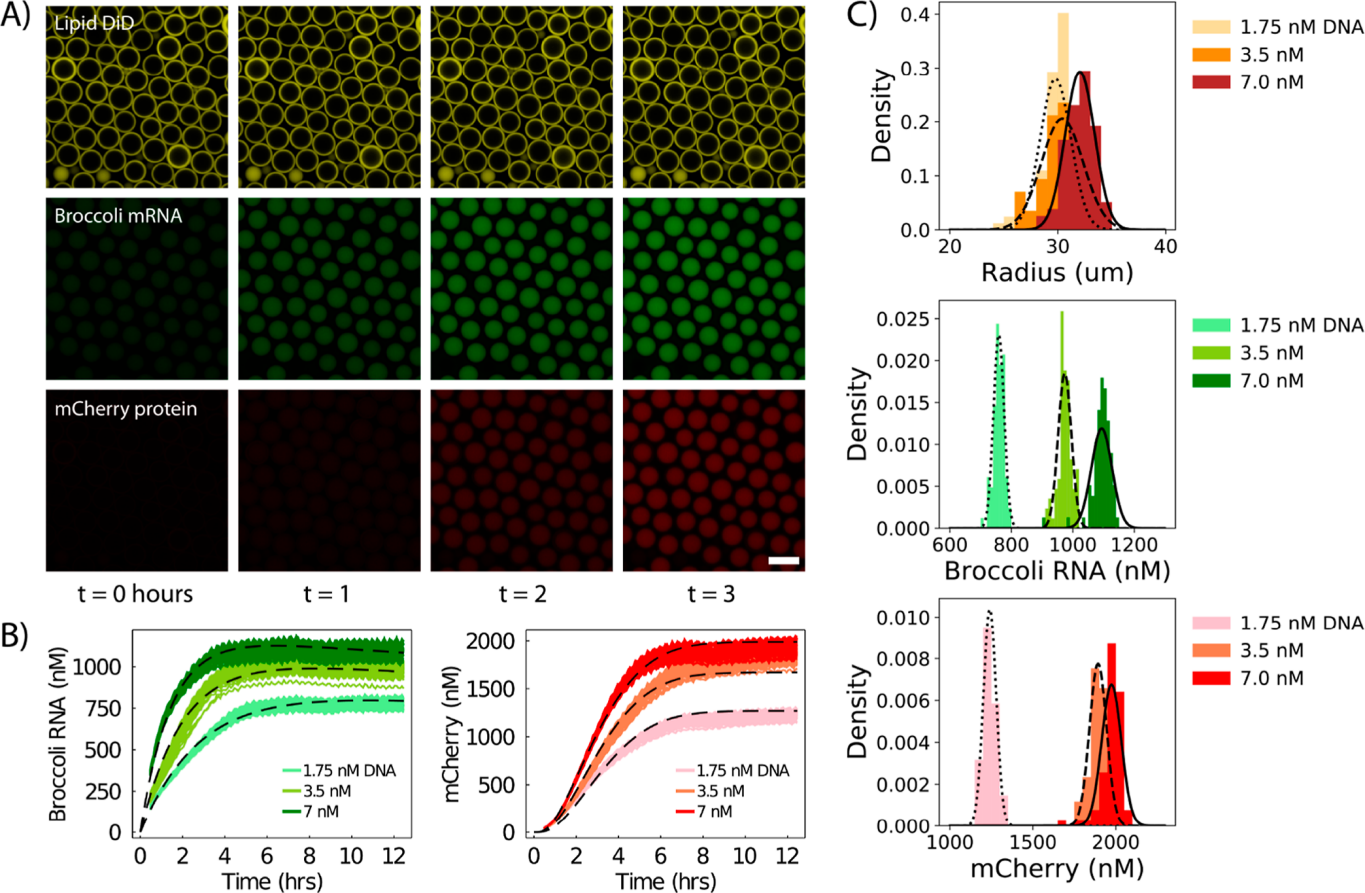
Quantifying transcription and translation dynamics in synthetic cell populations. (A) Timelapse confocal images of a synthetic cell population containing PURE CFES and 3.5 nM pEXP5-NT/6xHis mCherry F30-2xdBroccoli plasmid DNA. Images are divided into three channels: DiD dye-tagged lipid membrane (top row), Broccoli mRNA (middle row), and mCherry protein (bottom row). Timelapse images were taken every 5 min for a total of 12 h incubation at 30 °C using confocal microscopy. Scale bars are all 100 μm. (B) Single-cell traces of mRNA and protein expression in three synthetic cell populations with 1.75, 3.5, and 7.0 nM pEXP5-NT/6xHis mCherry F30-2xdBroccoli plasmid DNA with 82, 85, and 78 cells, respectively. RFU values are converted into nM concentrations units. (C) Endpoint distributions of radius, mRNA, and protein concentrations of the synthetic cell populations.

Using our microfluidic platform, we generated synthetic cell populations comprising large populations of monodisperse liposome-encapsulated CFESs and quantified RNA and protein levels over time to study transcription and translation dynamics using fluorescence microscopy methods. To alleviate non-identifiabilities during model fitting, we prepared three populations of synthetic cells with different DNA concentrations (1.75, 3.5, and 7.0 nM pEXP5-NT/6xHis mCherry F30-2xdBroccoli plasmid DNA) during one microfluidic session from one batch of CFES master mix and outer feeding buffer solution. RNA and protein levels in the synthetic cell populations were then monitored with confocal microscopy at 30 °C for 12 h (Figure 3A). Similar to the bulk CFES experiments, RFU was converted into absolute concentrations using a standard calibration curve, and our image analysis protocols were used (Sections 5 and 13 in the Supporting Information) to quantify RNA and protein dynamics in the synthetic cell populations (Figure 3B). Cell sizes from the three populations containing different plasmid DNA concentrations were monodisperse at ∼30 μm radius with a CV ranging from 0.04 to 0.065. The variability of gene expression from mRNA to protein remained constant with CV values ranging from 0.02 to 0.03 (Figure 3C and Table 2). This indicates a low degree of variability in translation across the synthetic cells, as CV values were not altered between mRNA and protein levels. Based on cell size and concentration measurements, copy numbers of DNA, mRNA, and protein molecules in a single synthetic cell are estimated to be in the order of 10^5^, 10^7^, and 10^8^, respectively. Other components required for gene expression in the PURExpress CFES are also present in similar or higher concentrations,^10,55^ such that stochastic effects associated with low copy numbers should be virtually absent. Time scales of active gene expression were comparable between bulk (Figure 1B) and encapsulated reactions (Figure 3B) (approx. 8 h). Maximum gene expression rates and endpoint mRNA and protein concentrations differ between the bulk expression and compartmentalized expression (Figures S14–S15 and S45–S46 in the Supporting Information). For example, protein expression levels in the liposomes are consistently lower than in bulk reactions. To quantify gene expression dynamics, mean RNA and protein dynamics from all three synthetic cell populations were globally fit to the resource-limited CFES model in eqs 1–6. Sample preparation of the synthetic cells typically took 0.5–1 h due to the encapsulation of different plasmid concentrations. As a result, the initial points of gene expression were not fully captured in the time series data. To account for sample preparation time, a time lag parameter (τ_l_) was included into the fitting procedure of the model for the time between starting the CFES reaction and acquisition of the first data point.

**Table 2:** Mean and Standard Deviations of the Microfluidic-Generated Synthetic Cell Populations^a^

Population	DNA (nM)	Radius (μm)	Broccoli RNA (nM)	mCherry (nM)
1	1.75	29.8 ± 1.4 (0.048)	759.0 ± 17.3 (0.022)	1240.5 ± 38.4 (0.031)
2	3.5	30.4 ± 1.9 (0.064)	973.8 ± 21.6 (0.022)	1892.7 ± 51.5 (0.028)
3	7.0	32.0 ± 1.4 (0.043)	1093.7 ± 33.5 (0.03)	1973.9 ± 59.1 (0.03)

a The total number of cells analyzed is 82, 85, and 78 for populations 1, 2, and 3, respectively.

The fitted rate parameters and 95% CIs obtained by fitting the experimental data to the model are shown in Table 1. It is important to note that the parameter estimation was performed on only one batch of experiments. This was done to avoid known batch-to-batch variability in CFESs that is not accounted for in our parameter estimation methodology. However, we also observed that different batches of synthetic cell populations prepared on different days can result in different endpoint protein concentrations and maximum translation rates with a batch-wise CV of 0.10 and 0.16, respectively (Figure S48 in the Supporting Information). This is comparable to previously reported batch CV values of expressed eGFP or RFP in bulk PURE systems at 0.05–0.2.^15,56^ The identifiable parameters are comparable to the bulk reaction parameters within one order of magnitude. However, more parameters were weakly identifiable as only three DNA concentrations (1.75, 3.5, and 7 nM) were considered for model fitting in the synthetic cell population experiment (Section 14 in the Supporting Information). The parameters *K*_l_ and τ_d_ were weakly identifiable, and *b* is non-identifiable. Additional populations were not prepared, as the time required to generate the populations of vesicles would have led to less data being obtained in the initial rates of gene expression, which are important for the purpose of parameter inference. We hypothesized that deviations of rate parameters between bulk and encapsulated formats are due to the different chemical conditions. Specifically, the composition of the outer feeding buffer solution can affect the inner CFES reaction by diffusion of materials across the semipermeable membrane.^6^ In preparing inner and outer solutions, we ensured that inner CFESs and outer solutions were osmotically balanced by matching freezing-point osmometer measurements. However, the outer feeding buffer and inner PURExpress CFES had slightly different compositions. To determine whether the composition of the outer solution would significantly affect gene expression in the microfluidic-generated synthetic cells, synthetic cell populations with identical inner and outer solutions (except for the DNA plasmid template in the outer solution) were compared with progressively diluted outer solutions. Diluted outer solutions resulted in a lower expression of RNA and protein in the synthetic cell populations, which shows that the composition of the outer solution influences the dynamics of the encapsulated CFES reaction (Figures S53 and S54 in the Supporting Information). The higher expression in the undiluted PURExpress outer solution agrees with previous experiments, showing that an outer solution that has been chemically tuned can improve gene expression in liposome-encapsulated CFES.^6,57^

## 3 Conclusions

In summary, our study tested different variations of a coarse-grained model of CFES reactions using simultaneously quantified RNA and protein dynamics with likelihood-based methods for model selection and parameter identification. Using a coarse-grained model, gene expression parameters were estimated without the knowledge of the full composition of the CFES. This is particularly useful for crude extract systems or proprietary CFESs such as the NEB PURExpress CFES that we used in this study. Several models have been developed to include more details of CFE reactions such as initiation and elongation factors^26^ or multiple translating ribosomes on an mRNA template.^24^ These models provide a more detailed interpretation of the data but require either additional information on the time-varying states of these gene expression factors or additional unknown parameters that can result in overparameterized models and non-identifiability. In the present study, a coarse-grained model of transcription and translation was able to recapitulate the full gene expression dynamics across DNA and RNA titration experiments. While we focused on a simple constitutively expressed gene in this model, it can be readily extended to more complex gene circuitry, CFES characteristics, and protein maturation properties. We then showed that large populations of highly monodisperse synthetic cells can be reproducibly generated using double-emulsion microfluidics. Gene expression in these synthetic cells is uniform and deterministic. Using our methodologies, we demonstrated that bulk and encapsulated CFES reactions result in different gene expression dynamics. These differences are attributed to the semipermeable lipid membrane, which allows the exchange of ions and water that alters the internal composition of the synthetic cells. This emphasizes the importance of the physical environment to compartmentalized biochemical reactions. Our results demonstrate a high degree of control over synthetic cell production and relative ease of analysis compared to synthetic cells with high variability generated by bulk encapsulation methods which will be critical for bottom-up synthetic biology to build synthetic multicellular systems.

## 4 Materials and Methods

### 4.1 Plasmid Design

The plasmids pEXP5-NT/6xHis eGFP^58^ and pEXP5-NT/6xHis mCherry^59^ were kindly provided by J. L. Ross Anderson, University of Bristol. These plasmids consist of a constitutive T7 RNA polymerase-mediated promoter with a strong ribosomal binding site to express 6xHis-tagged eGFP and mCherry fluorescent proteins, respectively. The pEXP5-NT/6xHis mCherry F30-2xdBroccoli plasmid was made by inserting the F30-2xdBroccoli fragment downstream the mCherry stop codon and upstream the terminator of the mCherry gene. This results in transcribed mRNA that includes the F30-2xBroccoli sequence but a translated protein without the F30-2xBroccoli sequence. The F30 structure acts as a stable RNA scaffold for the two dimeric Broccoli units (2xdBroccoli).^60,61^ Broccoli binds and activates the fluorescence of the small molecule (*Z*)-4-(3,5-difluoro-4-hydroxybenzylidene)-1,2-dimethyl-1*H*-imidazol-5(4*H*)-one (DFHBI) (Sigma, USA). Plasmid construction protocols and sequences are further described in Section 2 of the Supporting Information. The pEXP5-NT/6xHis mCherry F30-2xdBroccoli plasmid was sequenced, confirmed by Sanger sequencing, and is available in Addgene (www.addgene.org, plasmid ID 169233).

### 4.2 Bulk CFES Experiments

Bulk CFES expression experiments were run using a standard half-volume (12.5 μL) reaction mix of the PURExpress *in vitro* protein synthesis kit (NEB, USA). All CFES experiments were supplemented with sucrose at a final concentration of 80.4 mM. The additional sucrose was included to balance the osmolarity between inner and outer buffer solutions in the encapsulated experiments and was also included in the bulk experiments to maintain the same reaction conditions. To detect levels of the Broccoli RNA aptamer, 10 μM DFHBI was added in CFES reactions using the pEXP5-NT/6xHis mCherry F30-2xdBroccoli plasmid or its purified transcripts. All plasmid DNA templates were prepared and purified by ethanol precipitation using the QIAGEN plasmid maxi kit (QIAGEN, Germany) and then dissolved in nuclease-free water. 6xHis mCherry F30-2xdBroccoli mRNA transcripts were prepared by in vitro transcription of the pEXP5-NT/6xHis mCherry F30-2xdBroccoli plasmid using the HiScribe T7 high-yield RNA synthesis kit (NEB, USA), treated with DNAse I (NEB, USA), purified using the QIAGEN RNeasy mini kit, and dissolved in nuclease-free water. Triplicate CFES reactions with the required DNA or mRNA template concentrations were prepared in 384-well plates, sealed with a clear film, and incubated in a TECAN Spark 20M plate reader at 30 °C. Fluorescence measurements were undertaken for each sample at 10 min intervals for 8 h. Excitation and emission wavelengths used were 485/535, 570/620, and 450/510 nm with a bandwidth of ±20 nm each, for eGFP, mCherry, and Broccoli RNA, respectively. Fluorescence values were then converted into concentration units using a linear calibration curve from serial dilutions of purified eGFP protein, mCherry protein, and Broccoli RNA in the same CFES reaction mix and plate reader acquisition settings. Further details for the calibration and bulk CFES experiments are available in Sections 5–7 of the Supporting Information.

### 4.3 Encapsulated CFES Experiments

CFES reactions were encapsulated into liposomes using a double-emulsion microfluidic device and methodology as presented in ref (38). Inner CFES solutions were prepared with a plasmid DNA template similar to the bulk CFES experiments. The lipid oil phase was composed of 1-octanol (Sigma, USA) with 6.5 mM l-α-phosphatidylcholine (Egg PC) phospholipids (Avanti, USA) and 53.3 μM 1,1′-dioctadecyl-3,3,3′,3′-tetramethylindodicarbocyanine, 4-chlorobenzenesulfonate salt (DiD) fluorescent dye (Invitrogen, USA). The outer aqueous solution was composed of a CFES feeding buffer solution modified using previous work,^8^ which contains NTPs (6 mM ATP (Sigma, USA), 4 mM CTP (Sigma, USA), 4 mM UTP (Sigma, USA), 6 mM GTP (Roche, Switzerland)), amino acids (0.5 mM each) (Sigma, USA), 1.5 mM spermidine(Sigma, USA), 1.5 mM dithiothreitol (Thermo, USA), 0.02 mM folinic acid (Sigma, USA), 280 mM potassium glutamate (Sigma, USA), 20 mM magnesium glutamate (Sigma, USA), 100 mM HEPES (Roth, Germany), 480 mM glucose (Sigma, USA), and 2% (w/v) Pluronic F-68 (Gibco, USA) at pH 7.6. We can generate hundreds to thousands of synthetic cells per microfluidic session but typically prepare up to a hundred synthetic cells in a glass slide for imaging. Prepared synthetic cell populations were imaged by confocal laser scanning microscopy using an inverted Zeiss LSM 880 with Airyscan and a 10X/0.45 Plan-Apochromat M27 objective. The samples were maintained at 30 °C. Laser excitation wavelengths were 488, 488, 561, and 633 nm for Broccoli RNA, eGFP protein, mCherry protein, and DiD dye, respectively. Emission wavelengths were 499–561, 499–561, 579–641, and 640–720 nm for Broccoli RNA, eGFP protein, mCherry protein, and DiD dye detection, respectively. Images were focused at the equator of the synthetic cells and then acquired every 5 min for a total of 12 h. z-stack images of the samples were taken at the 12 h endpoint. Timelapse and z-stack images were processed using Fiji (v1.53c)^62^ and Python (v3.6) with Scikit-image.^63^ Synthetic cells were segmented, and fluorescence values for each cell were taken and converted to concentration units using a linear calibration curve from serial dilutions of purified eGFP protein, mCherry protein, and Broccoli RNA in bulk CFES reaction solutions with the same confocal microscopy acquisition settings and corrected for changes in laser power (Section 5 in the Supporting Information). Further details for microfluidic chip fabrication and pretreatment, bulk phase transfer and microfluidic CFES encapsulation, image analysis, and synthetic cell population experiments are available in Sections 10–16 in the Supporting Information.

### 4.4 CFES Model Selection and Parameter Estimation

A cell-free gene expression model was developed based on a previously published resource-limited gene expression model.^22^ We tested seven variations of the model using mass action or Michaelis–Menten kinetics for transcription and translation and the degradation and consumption of transcription resources (TsR) and translation resources (TlR). These models were fit on the Broccoli RNA aptamer and mCherry protein time series data from our bulk experiments. The agreement between the experimental data and model was measured by the negative natural logarithm of the likelihood of the model parameters given the experimental data
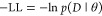
7where θ = {θ_1_, ···, θ_k_} is the set of parameters for the model and *D* is the experimental data. The term on RHS is the log-likelihood of observing data *D* given model parameters θ. Rate parameters of a model were estimated by minimizing the log-likelihood, that is
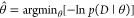
8where θ̂ is the maximum likelihood estimator (MLE) of the model parameters. The different models were ranked according to the AIC
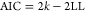
9where *k* is the number of parameters and LL is the log-likelihood evaluated at the MLE θ̂. The models with the lowest AIC values were selected and used for profile likelihood analysis. Profile likelihoods and likelihood-based CIs from the parameter estimates were calculated to assess the parameter identifiability.^48,49^ The profile likelihoods of each parameter were calculated by
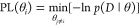
10

which is the minimum of the negative log-likelihood with respect to all parameters θ_*j*≠*i*_ while holding the parameter θ_*i*_ fixed. Likelihood-based CIs of each parameter were estimated by the regions in
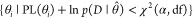
11where χ^2^(α,df) is the chi-squared distribution with α = 0.95 confidence level and df is the degree of freedom, which is the number of parameters of the model.^49^ The final model used (eqs 1–6) was chosen based on the AIC. Further details and results of the model selection and parameter estimation are available in Section 9 of the Supporting Information.

## Supporting Information Available

The Supporting Information is available free of charge at https://pubs.acs.org/doi/10.1021/acssynbio.1c00376. Data, code, and plasmid sequences used in this study available in https://dx.doi.org/10.17617/3.77. The pEXP5-NT/6xHis mCherry F30-2xdBroccoli plasmid generated in this study is available in Addgene (addgene.org) with plasmid ID 169233.

Chemicals, materials, and equipment; plasmid construction; mRNA expression and purification; protein expression and purification; protein and mRNA calibration; bulk cell-free expression experiments; mCherry maturation; resource-limited gene expression models; parameter estimation, model selection, and profile likelihoods; microfluidic chip fabrication and pretreatment; encapsulation of CFES in liposomes using microfluidics; inverse emulsion phase transfer method; image acquisition and analysis; DNA titration series in synthetic cell populations; two plasmids in a synthetic cell population; dilution of CFES outer solution in synthetic cell populations (PDF)
