# Correction to “Cell-Free Gene Expression Dynamics in Synthetic Cell Populations”

**DOI:** 10.1021/acssynbio.4c00583

**Published:** 2024-10-01

**Authors:** David T. Gonzales, Naresh Yandrapalli, Tom Robinson, Christoph Zechner, T-Y. Dora Tang

**Affiliations:** † Max Planck Institute of Molecular Cell Biology and Genetics, 01307 Dresden, Germany; ‡ Center for Systems Biology Dresden, 01307 Dresden, Germany; § Max Planck Institute of Colloids and Interfaces, 14476 Potsdam, Germany; ∥ Physics of Life, Cluster of Excellence, TU Dresden, 01603 Dresden, Germany

After publication, we identified an error in the calculation of the likelihood threshold used to determine approximate 95%-confidence intervals from profile likelihoods. The likelihood-based confidence intervals are estimated as the regions with
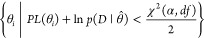
where χ^2^(*α,df*) is the α-quantile of the chi-squared distribution with *df* degrees of freedom, which is set to the number of model parameters. In our original publication, the factor 1/2 with χ^2^(*α,df*) was omitted in eq 11 and eq S34. Our error resulted in an overestimation of each parameter’s 95%-confidence interval. The maximum likelihood estimates of the parameters themselves were not affected by the mistake. After correcting this in our calculations, the confidence intervals became narrower. In one instance, this caused a parameter, which was previously found to be weakly identifiable, to become identifiable (*K*_*l*_). All original and corrected items are provided.
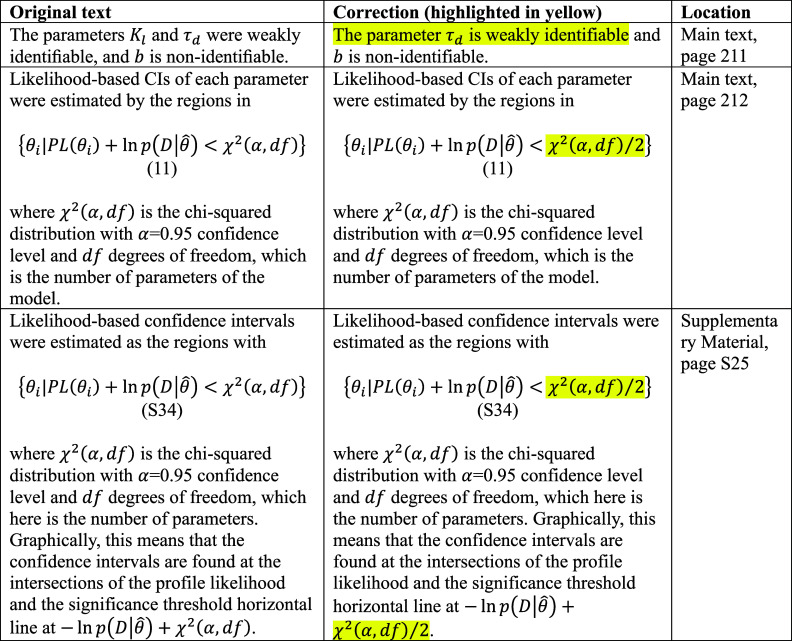


**Table 1a:**
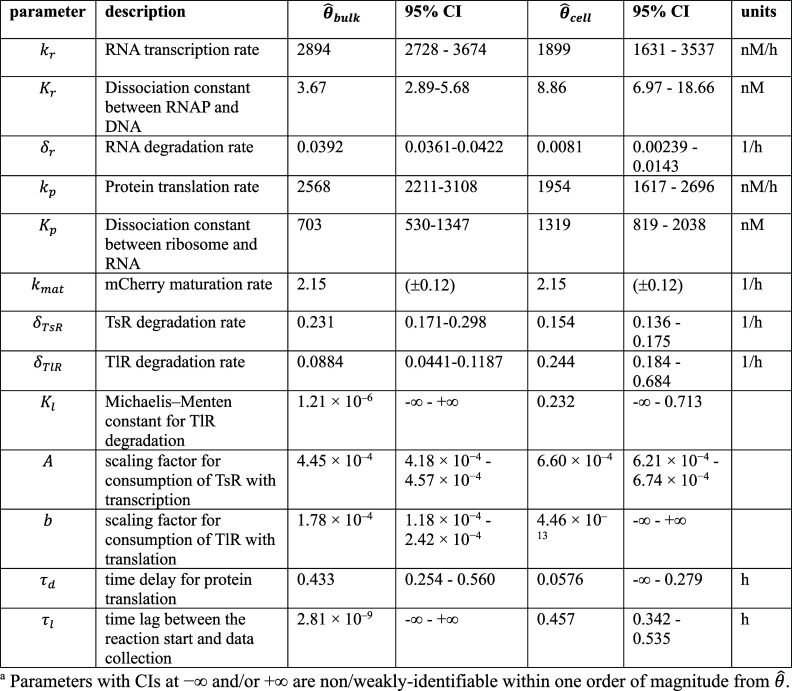
**(Original)** Parameter Estimates and Likelihood-Based 95% CI from the Resource-Limited Gene Expression Model Fitting on Bulk DNA and RNA Titration Experiments (θ̂_bulk_) and Synthetic Cell Population DNA Titration Experiments (θ̂_cell_)^*a*^

**Table 1b:**
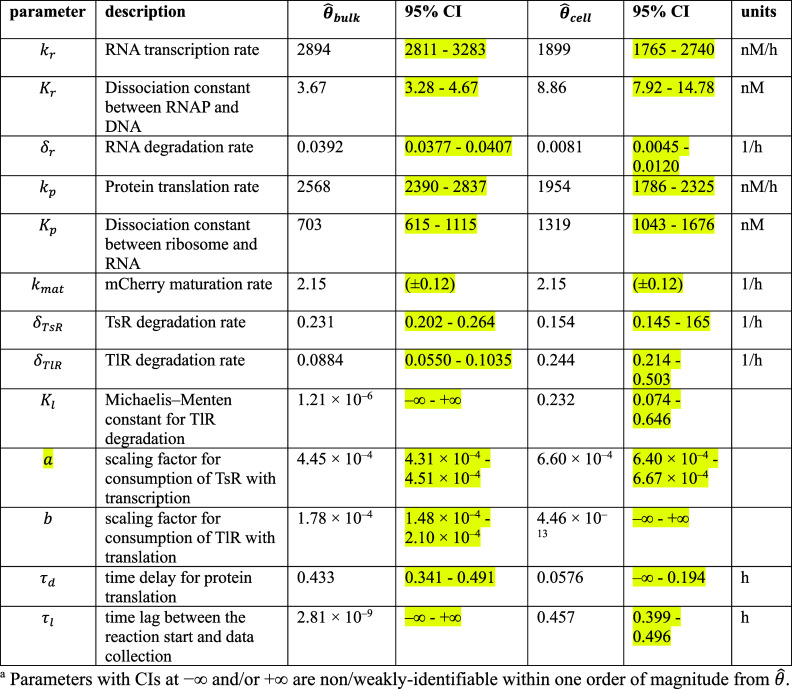
**(Corrected)** Parameter Estimates and Likelihood-Based 95% CI from the Resource-Limited Gene Expression Model Fitting on Bulk DNA and RNA Titration Experiments (θ̂_bulk_) and Synthetic Cell Population DNA Titration Experiments (θ̂_cell_)^*a*^

**Table S26a:**
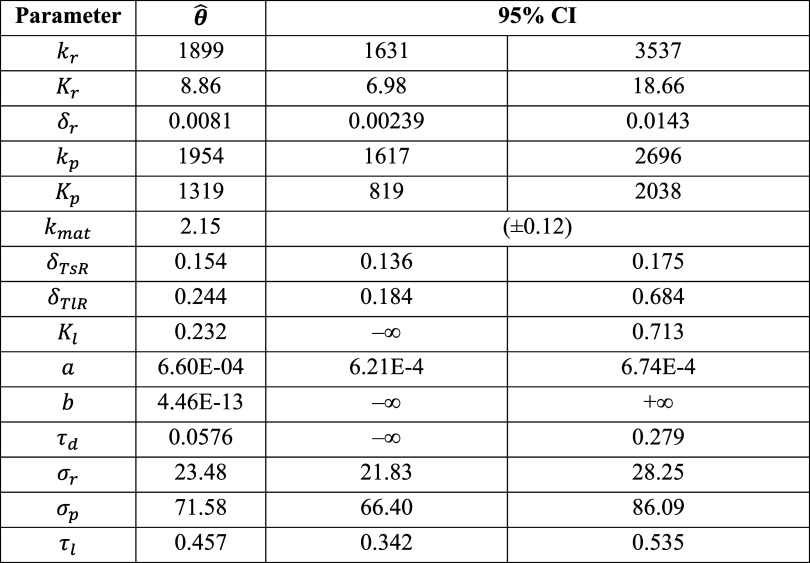
**(Original)** Parameter Estimates and 95% Likelihood-Based Confidence Intervals of Model 2 Using Synthetic Cell Population Data

**Table S26b:**
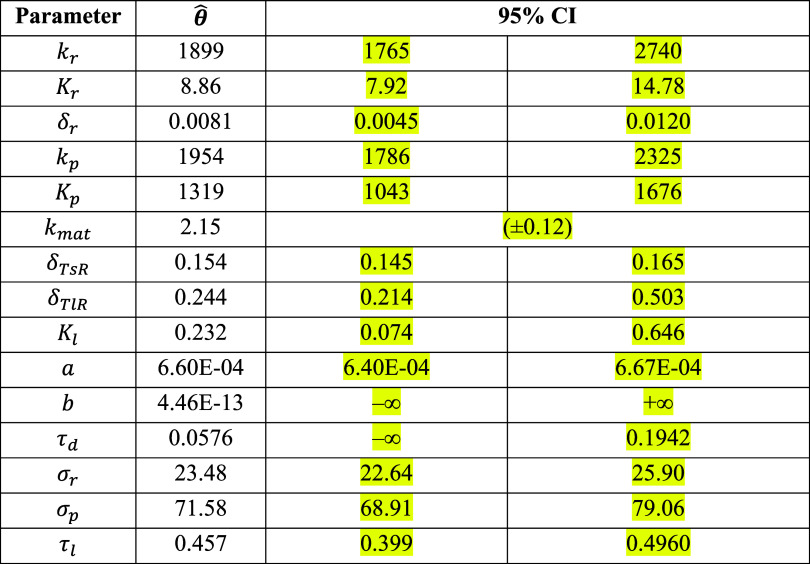
**(Corrected)** Parameter Estimates and 95% Likelihood-Based Confidence Intervals of Model 2 Using Synthetic Cell Population Data

**Figure S25a:**
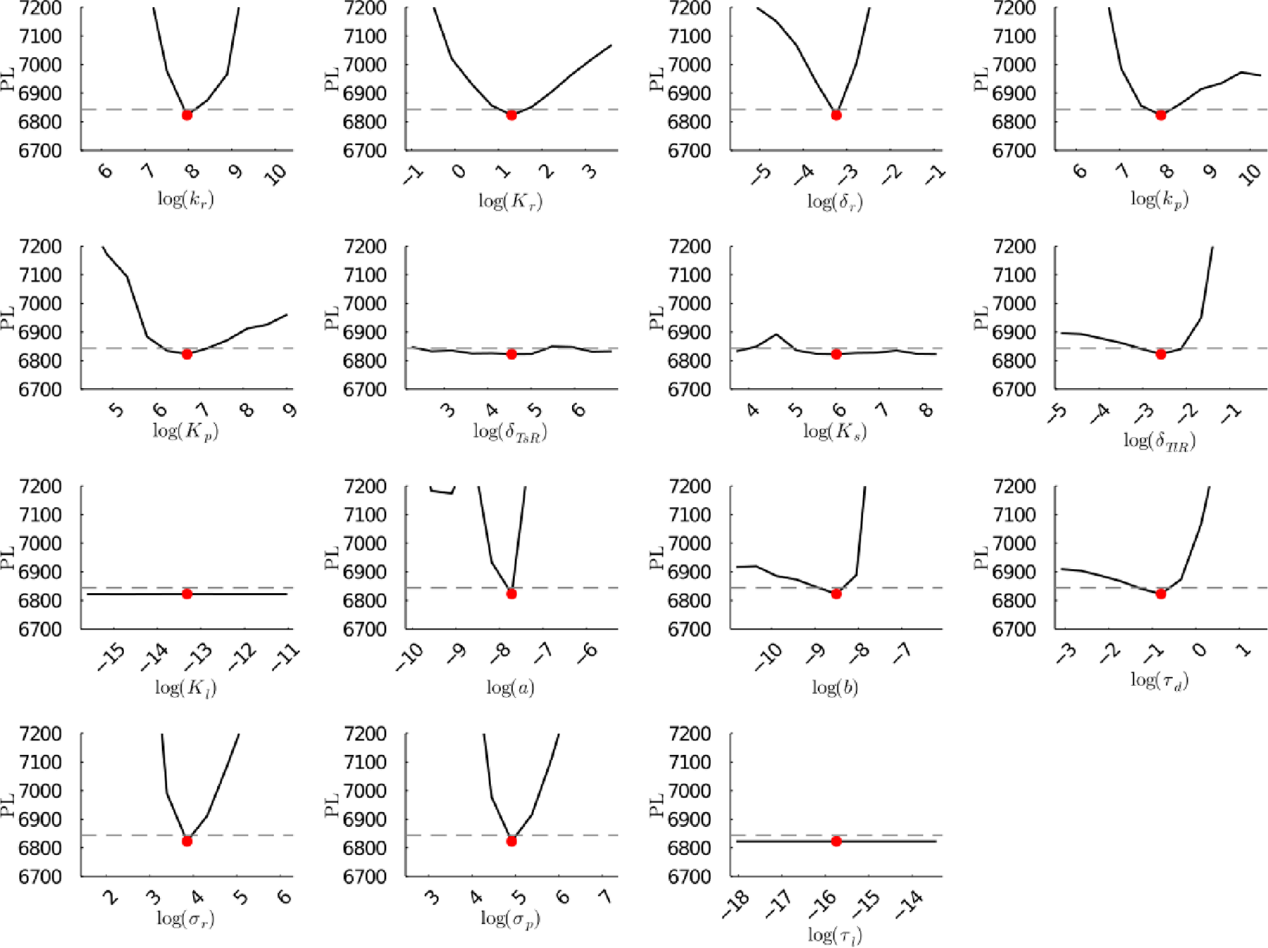
**(Original)** Profile likelihoods of parameters from model 1. Each plot corresponds to a parameter in the model and additional fitting parameters (σ_*r*_, σ_*p*_, τ_*d*_). The *y*-axis of each plot shows the minimized negative log-likelihood of the model with respect to all parameters except for the parameter given its value in the *x*-axis. The red dot shows the optimized parameter set with the negative MLE. The dashed gray line is the 95% significance threshold line. All logarithms are in natural log.

**Figure S25b:**
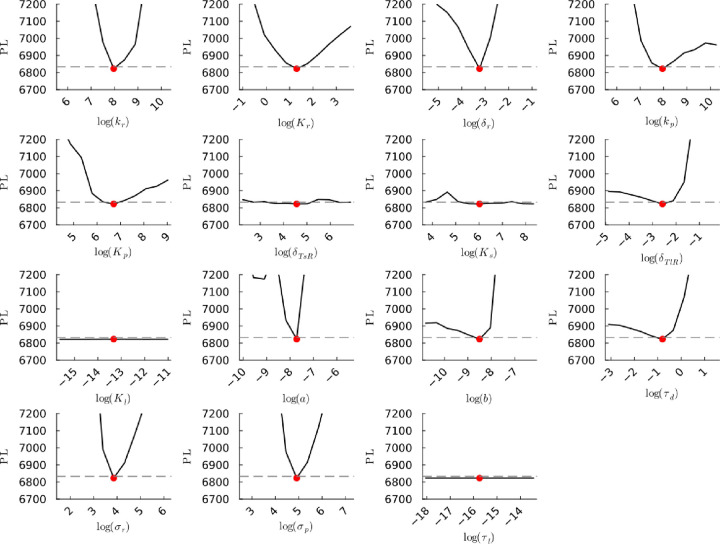
**(Corrected)** Profile likelihoods of parameters from model 1. Each plot corresponds to a parameter in the model and additional fitting parameters (σ_*r*_, σ_*p*_, τ_*d*_). The *y*-axis of each plot shows the minimized negative log-likelihood of the model with respect to all parameters except for the parameter given its value in the *x*-axis. The red dot shows the optimized parameter set with the negative MLE. The dashed gray line is the 95% significance threshold line. All logarithms are in natural log.

**Figure S26a:**
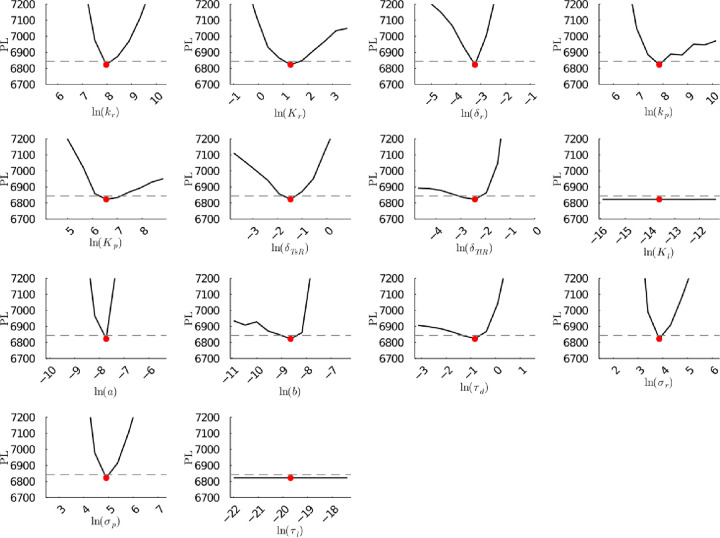
**(Original)** Profile likelihoods of parameters from model 2. Each plot corresponds to a parameter in the model and additional fitting parameters (σ_*r*_, σ_*p*_, τ_*d*_). The *y*-axis of each plot shows the minimized negative log-likelihood of the model with respect to all parameters except for the parameter given its value in the *x*-axis. The red dot shows the optimized parameter set with the negative MLE. The dashed gray line is the 95% significance threshold line. All logarithms are in natural log.

**Figure S26b:**
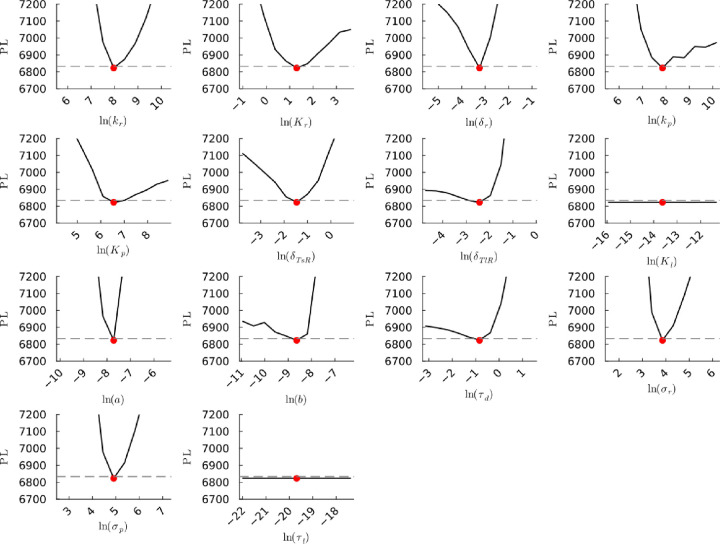
**(Corrected)** Profile likelihoods of parameters from model 2. Each plot corresponds to a parameter in the model and additional fitting parameters (σ_*r*_, σ_*p*_, τ_*d*_). The *y*-axis of each plot shows the minimized negative log-likelihood of the model with respect to all parameters except for the parameter given its value in the *x*-axis. The red dot shows the optimized parameter set with the negative MLE. The dashed gray line is the 95% significance threshold line. All logarithms are in natural log.

**Figure S27a:**
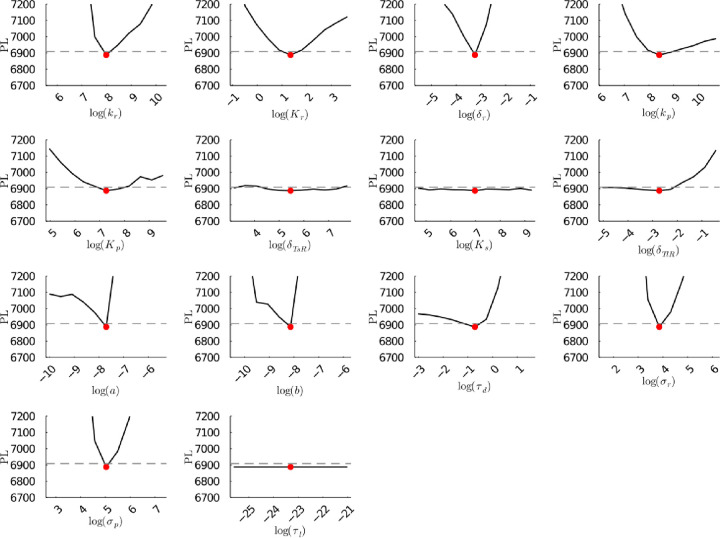
**(Original)** Profile likelihoods of parameters from model 3. Each plot corresponds to a parameter in the model and additional fitting parameters (σ_*r*_, σ_*p*_, τ_*d*_). The *y*-axis of each plot shows the minimized negative log-likelihood of the model with respect to all parameters except for the parameter given its value in the *x*-axis. The red dot shows the optimized parameter set with the negative MLE. The dashed gray line is the 95% significance threshold line. All logarithms are in natural log.

**Figure S27b:**
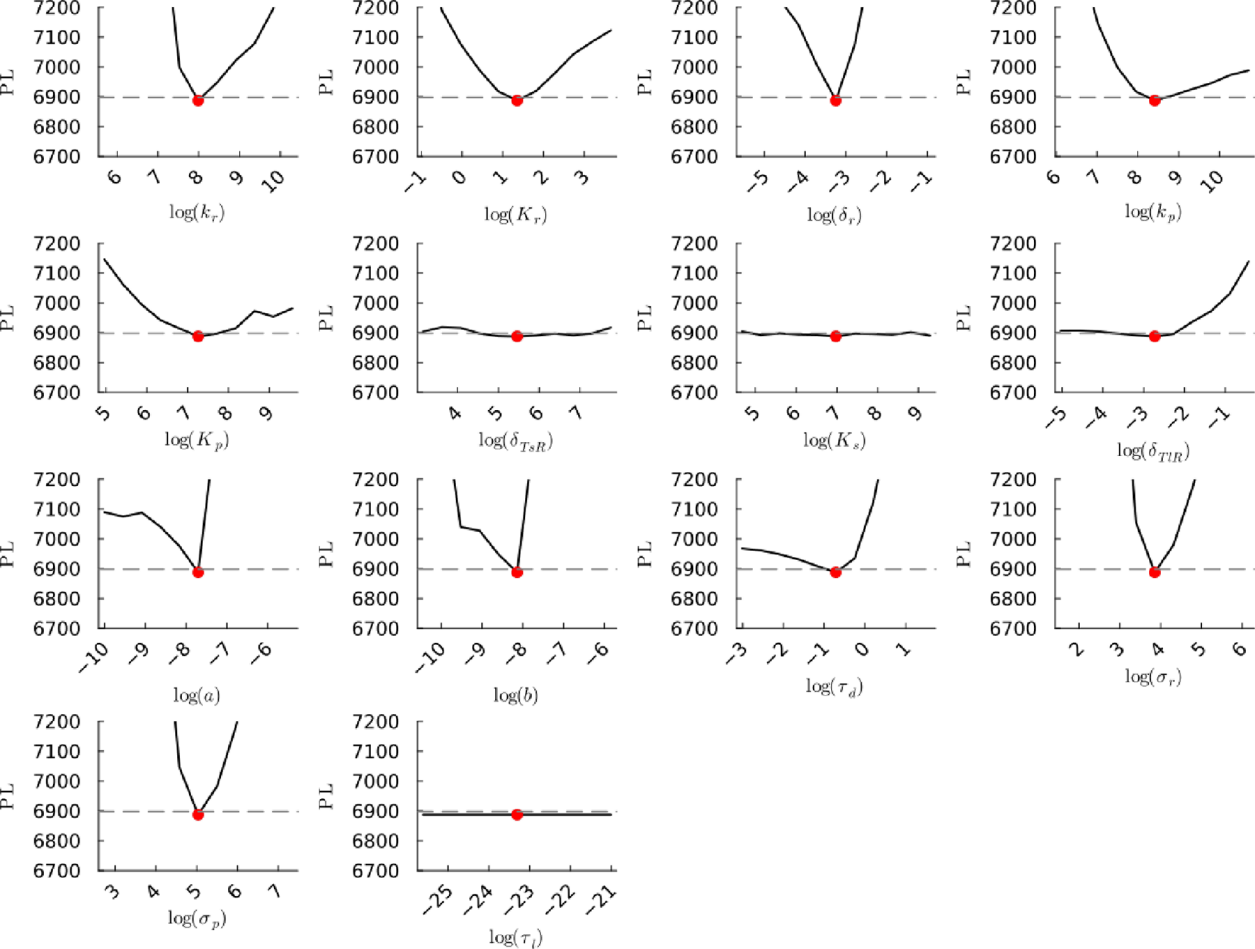
**(Corrected)** Profile likelihoods of parameters from model 3. Each plot corresponds to a parameter in the model and additional fitting parameters (σ_*r*_, σ_*p*_, τ_*d*_). The *y*-axis of each plot shows the minimized negative log-likelihood of the model with respect to all parameters except for the parameter given its value in the *x*-axis. The red dot shows the optimized parameter set with the negative MLE. The dashed gray line is the 95% significance threshold line. All logarithms are in natural log.

**Figure S28a:**
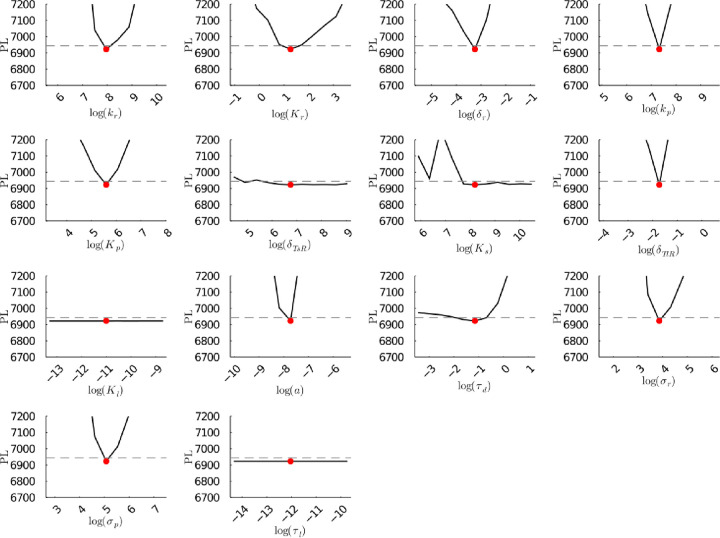
**(Original)** Profile likelihoods of parameters from model 4. Each plot corresponds to a parameter in the model and additional fitting parameters (σ_*r*_, σ_*p*_, τ_*d*_). The *y*-axis of each plot shows the minimized negative log-likelihood of the model with respect to all parameters except for the parameter given its value in the *x*-axis. The red dot shows the optimized parameter set with the negative MLE. The dashed gray line is the 95% significance threshold line. All logarithms are in natural log.

**Figure S28b:**
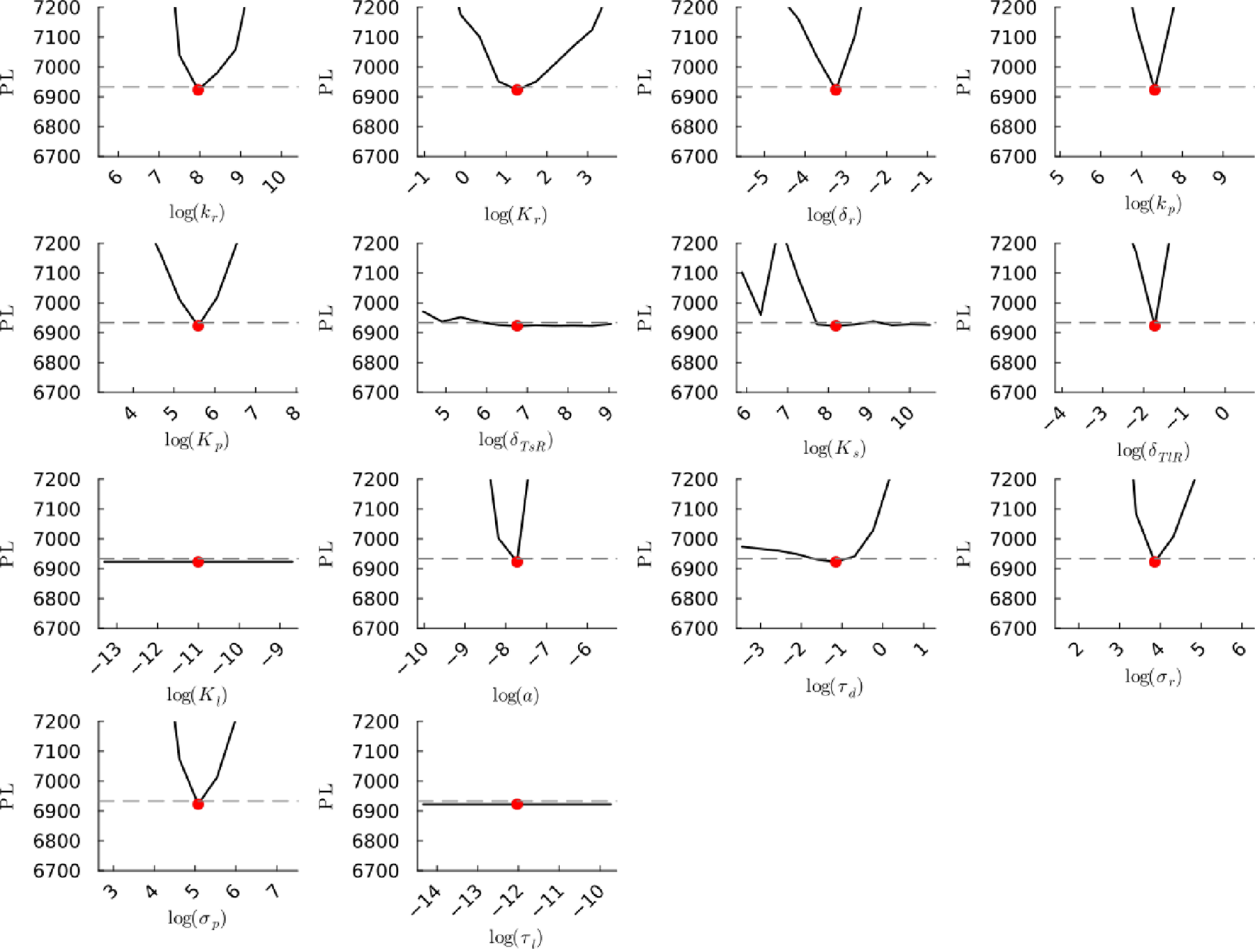
**(Corrected)** Profile likelihoods of parameters from model 4. Each plot corresponds to a parameter in the model and additional fitting parameters (σ_*r*_, σ_*p*_, τ_*d*_). The *y*-axis of each plot shows the minimized negative log-likelihood of the model with respect to all parameters except for the parameter given its value in the *x*-axis. The red dot shows the optimized parameter set with the negative MLE. The dashed gray line is the 95% significance threshold line. All logarithms are in natural log.

**Figure S29a:**
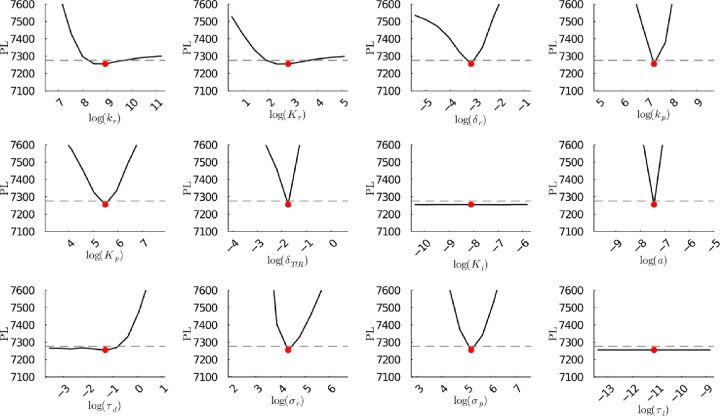
**(Original)** Profile likelihoods of parameters from model 5. Each plot corresponds to a parameter in the model and additional fitting parameters (σ_*r*_, σ_*p*_, τ_*d*_). The *y*-axis of each plot shows the minimized negative log-likelihood of the model with respect to all parameters except for the parameter given its value in the *x*-axis. The red dot shows the optimized parameter set with the negative MLE. The dashed gray line is the 95% significance threshold line. All logarithms are in natural log.

**Figure S29b:**
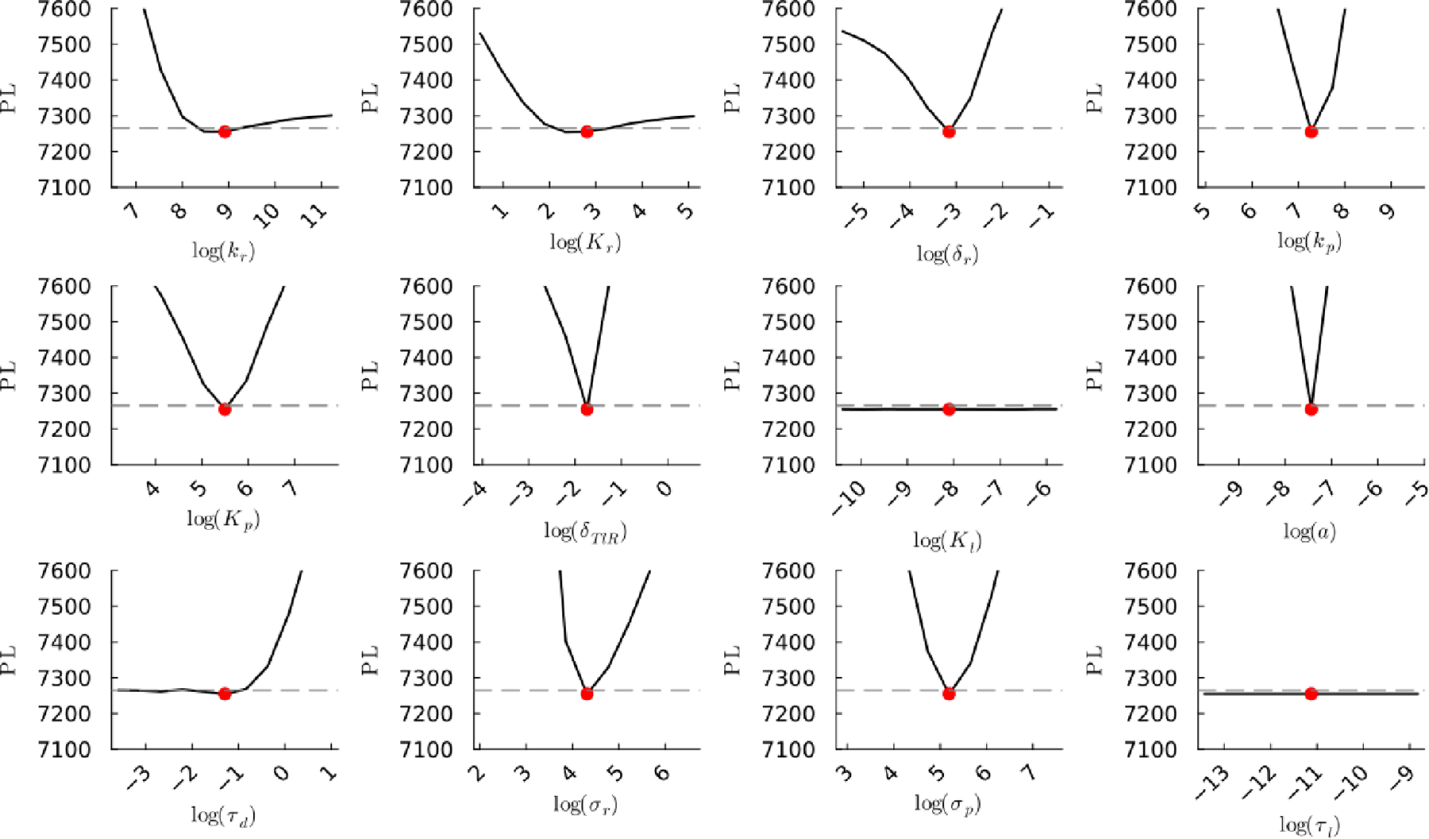
**(Corrected)** Profile likelihoods of parameters from model 5. Each plot corresponds to a parameter in the model and additional fitting parameters (σ_*r*_, σ_*p*_, τ_*d*_). The *y*-axis of each plot shows the minimized negative log-likelihood of the model with respect to all parameters except for the parameter given its value in the *x*-axis. The red dot shows the optimized parameter set with the negative MLE. The dashed gray line is the 95% significance threshold line. All logarithms are in natural log.

**Figure S30a:**
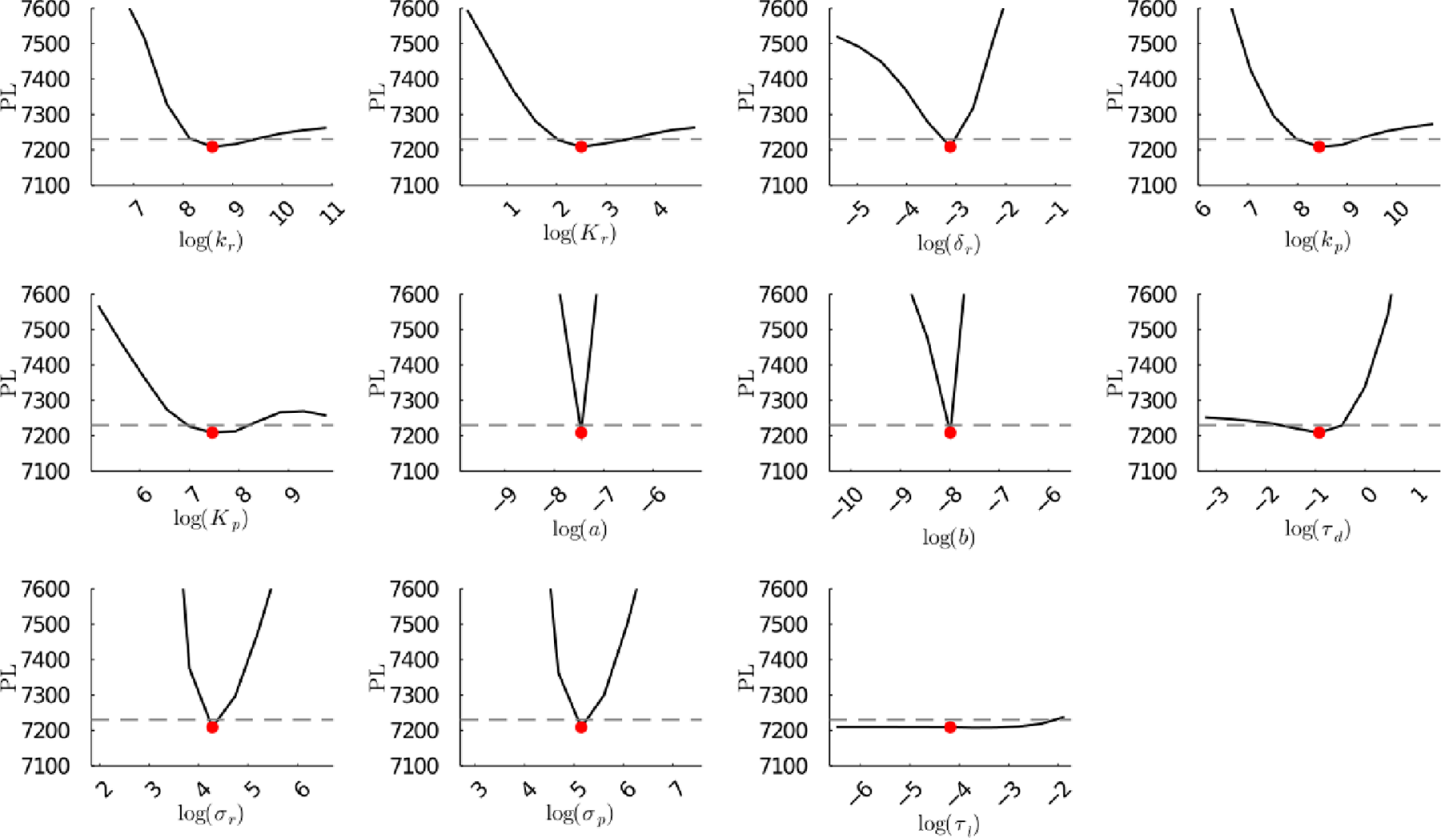
**(Original)** Profile likelihoods of parameters from model 6. Each plot corresponds to a parameter in the model and additional fitting parameters (σ_*r*_, σ_*p*_, τ_*d*_). The *y*-axis of each plot shows the minimized negative log-likelihood of the model with respect to all parameters except for the parameter given its value in the *x*-axis. The red dot shows the optimized parameter set with the negative MLE. The dashed gray line is the 95% significance threshold line. All logarithms are in natural log.

**Figure S30b:**
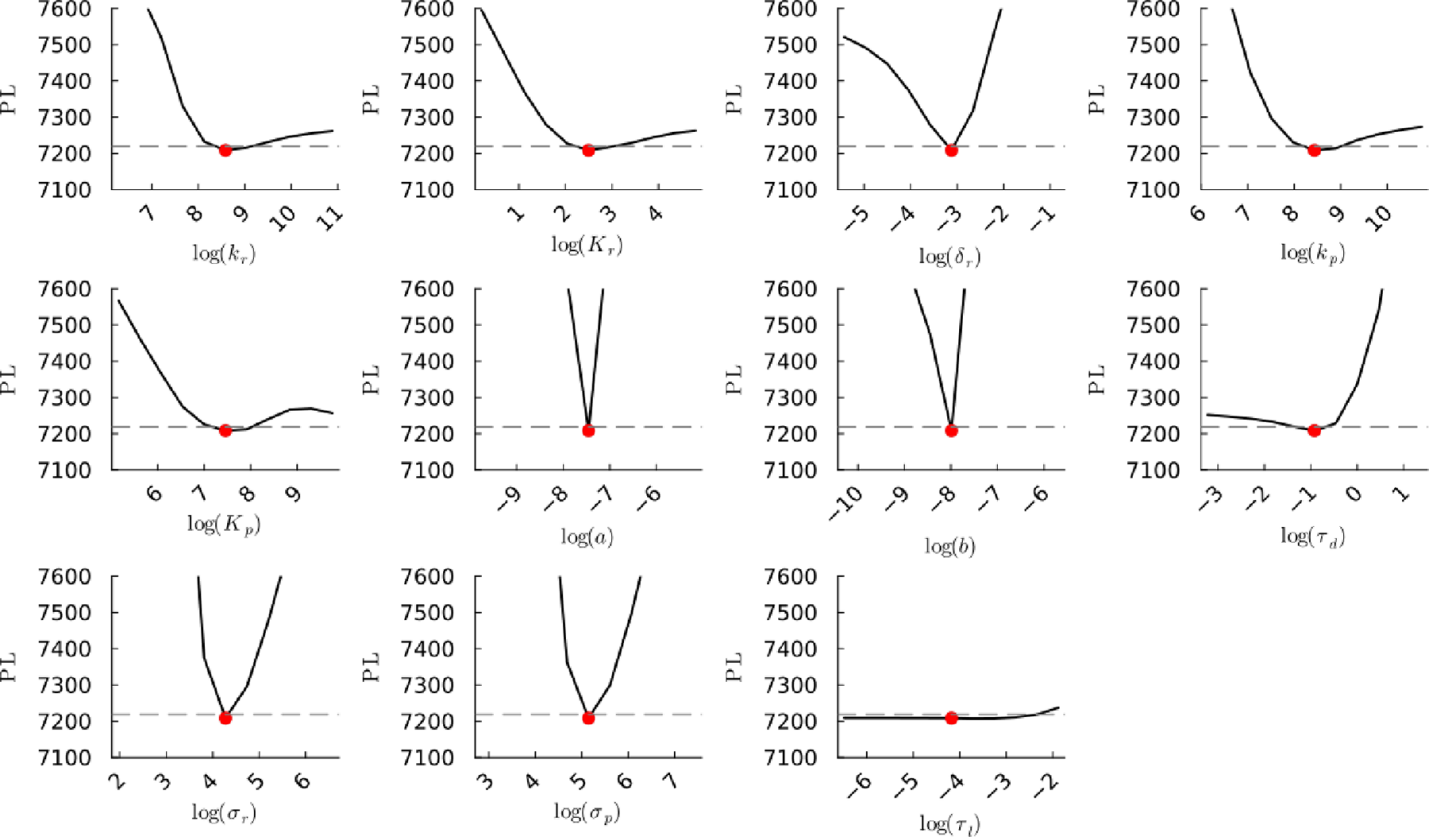
**(Corrected)** Profile likelihoods of parameters from model 6. Each plot corresponds to a parameter in the model and additional fitting parameters (σ_*r*_, σ_*p*_, τ_*d*_). The *y*-axis of each plot shows the minimized negative log-likelihood of the model with respect to all parameters except for the parameter given its value in the *x*-axis. The red dot shows the optimized parameter set with the negative MLE. The dashed gray line is the 95% significance threshold line. All logarithms are in natural log.

**Figure S49a:**
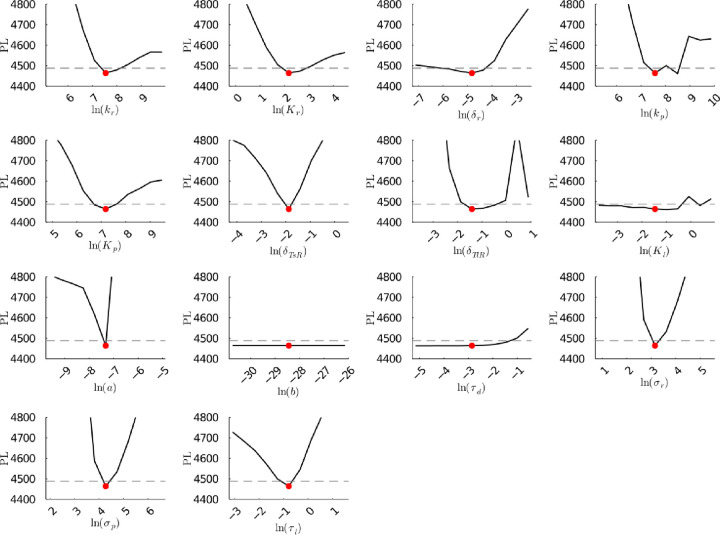
**(Original)** Profile likelihoods of parameters from model 2 using synthetic population experiment data. Each plot corresponds to a parameter in the model and additional fitting parameters (σ_*r*_, σ_*p*_, τ_*d*_). The *y*-axis of each plot shows the minimized negative log-likelihood of the model with respect to all parameters except for the parameter given its value in the *x*-axis. The red dot shows the optimized parameter set with the minimum negative log likelihood. The dashed gray line is the 95% significance threshold line. The intersections of the significance threshold line and profile likelihood are the likelihood-based confidence intervals of the optimized parameter.

**Figure S49b:**
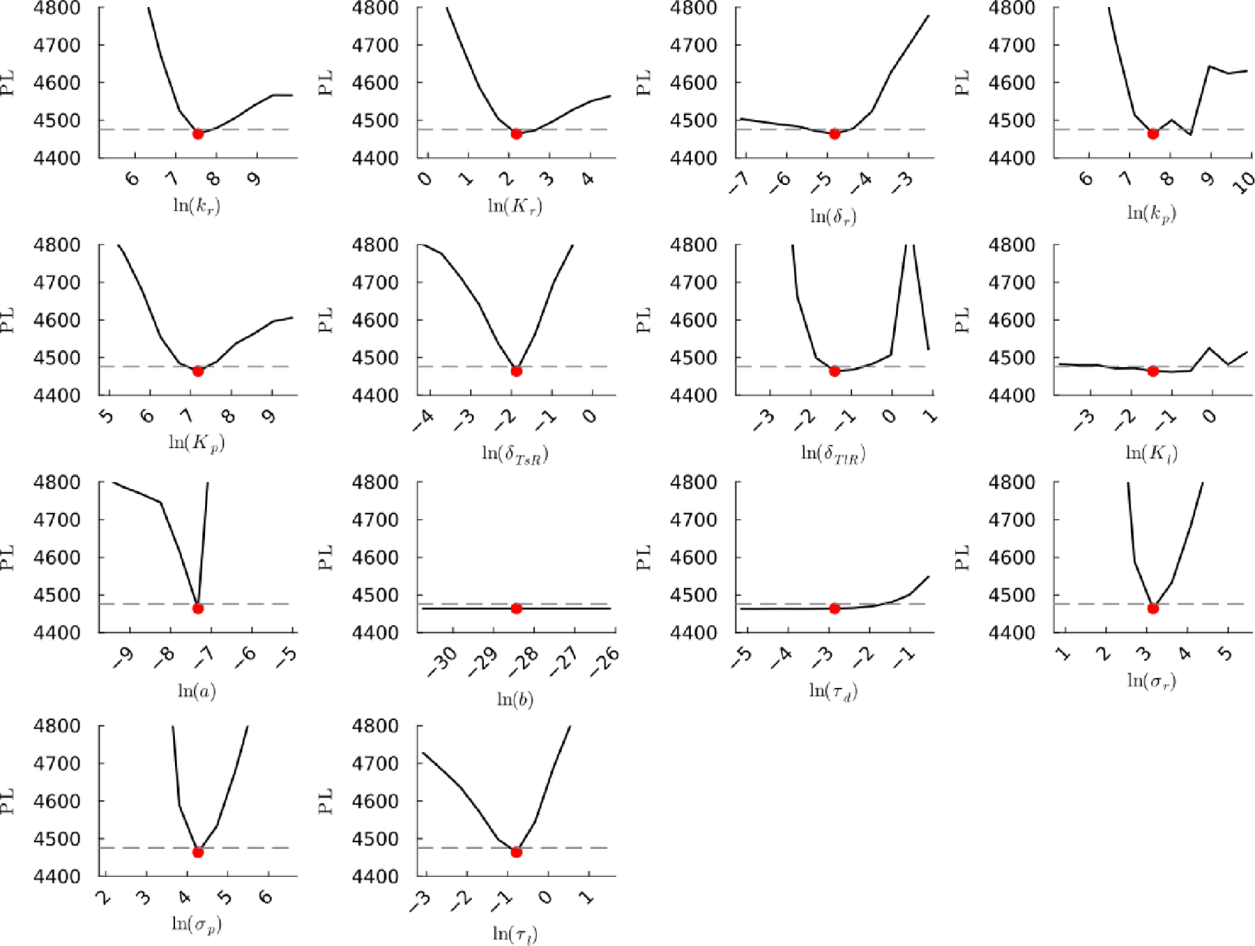
**(Corrected)** Profile likelihoods of parameters from model 2 using synthetic population experiment data. Each plot corresponds to a parameter in the model and additional fitting parameters (σ_*r*_, σ_*p*_, τ_*d*_). The *y*-axis of each plot shows the minimized negative log-likelihood of the model with respect to all parameters except for the parameter given its value in the *x*-axis. The red dot shows the optimized parameter set with the minimum negative log likelihood. The dashed gray line is the 95% significance threshold line. The intersections of the significance threshold line and profile likelihood are the likelihood-based confidence intervals of the optimized parameter.

