# Supplementary material for: Cell-Free Gene Expression Dynamics in Synthetic Cell Populations
Source: ACS Synth Biol. 2022 Jan 4;11(1):205–15. doi: 10.1021/acssynbio.1c00376 (PMC8787815; doi:10.1021/acssynbio.1c00376)
Supplement: Supplementary file 1 — sb1c00376_si_001.pdf [file sb1c00376_si_001.pdf]

Supplementary material

Cell-free gene expression dynamics in synthetic cell populations

David T. Gonzales[a,b], Naresh Yandrapalli[c], Tom Robinson[c], Christoph Zechner[a,b,d,*], T-Y Dora Tang[a,b,d,*]

[a] Max Planck Institute of Molecular Cell Biology and Genetics, 01307 Dresden, Germany
[b] Center for Systems Biology Dresden, 01307 Dresden, Germany
[c] Max Planck Institute of Colloids and Interfaces, 14476 Potsdam, Germany
[d] Physics of Life, Cluster of Excellence, TU Dresden, 01603 Dresden, Germany
* Correspondence to: zechner@mpi-cbg.de and tang@mpi-cbg.de

Contents

1. Chemicals, materials, and equipment
2. Plasmid construction
3. mRNA expression and purification
4. Protein expression and purification
5. Protein and mRNA calibration
6. Bulk cell-free expression experiments
7. mCherry maturation
8. Resource-limited gene expression models
9. Parameter estimation, model selection, and profile likelihoods
10. Microfluidic chip fabrication and pretreatment
11. Encapsulation of CFES in liposomes using microfluidics
12. Inverse emulsion phase transfer method
13. Image acquisition and analysis
14. DNA titration series in synthetic cell populations
15. Two plasmids in a synthetic cell population
16. Dilution of CFES outer solution in synthetic cell populations

All data and code used for analysis can be accessed at https://dx.doi.org/10.17617/3.77.

1. Chemicals, materials, and equipment

Table S1. Chemical list.

Name	MW (g/mole)	Supplier	Catalog No.
Amino acids			
L-Alanine (A)	89.09	Sigma, USA	A7627
L-Arginine (R)	174.20	Sigma, USA	A5006
L-Asparagine (N)	132.12	Sigma, USA	A0884
L-Aspartic acid (D)	133.10	Sigma, USA	A9256
L-Cysteine (C)	121.16	Sigma, USA	W326305
L-Glutamic acid (E)	147.13	Sigma, USA	G1251
L-Glutamine (Q)	146.14	Sigma, USA	G3126
Glycine (G)	75.07	Sigma, USA	G7126
L-Histidine (H)	155.15	Sigma, USA	H8000
L-Isoleucine (I)	131.17	Sigma, USA	I2752
L-Leucine (L)	131.17	Sigma, USA	L8000
L-Lysine (K)	146.19	Sigma, USA	L5501
L-Methionine (M)	149.21	Sigma, USA	M9625
L-Phenylalanine (F)	165.19	Sigma, USA	P2126
L-Proline (P)	115.13	Sigma, USA	P0380
L-Serine (S)	105.09	Sigma, USA	S4500
L-Threonine (T)	119.12	Sigma, USA	T8625
L-Tryptophan (W)	204.23	Sigma, USA	T0254
L-Tyrosine (Y)	181.19	Sigma, USA	T3754
L-Valine (V)	117.15	Sigma, USA	V0500
NTPs			
ATP (Adenosine 5′-triphosphate disodium salt hydrate)	551.14	Sigma, USA	A26209
CTP (Cytidine 5′-triphosphate disodium salt hydrate)	527.12	Sigma, USA	30320
GTP (Guanosine 5′-triphosphate sodium salt hydrate)	523.18	Roche, Switzerland	10106399001
UTP (Uridine 5′-triphosphate trisodium salt dihydrate)	586.12	Sigma, USA	94370
Enzymes			
Benzonase*		PEPC, MPI-CBG	-
DNAse I		NEB, USA	M0303
DpnI		NEB, USA	R0176
Phusion HF		NEB, USA	M0531
RNAseA		Carl Roth, Germany	7156.1
Ladders			
1kb DNA ladder		NEB, USA	N3232
PageRuler Plus protein ladder		Thermo, USA	26616
Riboruler High Range RNA ladder		Thermo, USA	SM1821
Dyes			
DFHBI ((Z)-4-(3,5-difluoro-4-hydroxybenzylidene)-1,2-dimethyl-1H-imidazol-5(4H)-one)	252.22	Sigma, USA	SML1627
DiD (1,1'-Dioctadecyl-3,3,3',3'-Tetramethylindodicarbocyanine, 4-Chlorobenzenesulfonate Salt)	1052.08	Invitrogen, USA	D7757
Gel Loading Dye		NEB, USA	B7024
GelRed		Biotium, USA	41003
Laemmli buffer		Bio-rad, USA	1610737
SYBRgold		Thermo, USA	S11494

Texas Red DHPE (N-(Texas Red sulfonyl)-1,2-dihexadecanoyl-snglycero-3-phosphoethanolamine, triethylammonium salt)	1380.77	Biotium, USA	60027
Others			
1H,1H,2H,2H-perfluorodecyltrichlorosilane	681.57	Sigma, USA	729965
1-Octanol	130.23	Sigma, USA	297887
40% Polyacrylamide solution	-	Bio-rad, USA	1610144
Agarose	-	Invitrogen, USA	16500
Ammonium peroxydisulphate	228.20	Carl Roth, Germany	9592.3
Ampicillin sodium salt	371.39	Sigma, USA	A9518
Protease Inhibitor Cocktail	-	Roche, Switzerland	COEDTAF-RO
Dithiothreitol	154.253	Thermo, USA	R0862
DMSO	78.13	Sigma, USA	D8418
Egg PC (L-α-phosphatidylcholine)	770.123	Avanti, USA	840051
Folinic acid calcium salt hydrate	511.50	Sigma, USA	F7878
D-(+)-Glucose	180.16	Sigma, USA	G8270
Glycerol	92.09	VWR, USA	24388.295
Gonotec calibration std. (300 mOsmol/kg)	-	Gonotec, Germany	30.9.0020
Gonotec calibration std. (2000 mOsmol/kg)	-	Gonotec, Germany	30.9.2000
HEPES (N-2-Hydroxyethylpiperazine-N'-2-ethane sulphonic acid)	238.31	Carl Roth, Germany	9105
Hydrochloric acid fuming 37%	36.46	Merck, USA	100317
Hydrogen peroxide	34.01	Sigma, USA	16911
Imidazole	68.08	AppliChem	A1073
Magnesium chloride hexahydrate	203.30	Sigma, USA	105833
L-Glutamic acid hemimagnesium salt tetrahydrate	388.61	Sigma, USA	49605
Mineral oil	(0.84 g/mL) (1.467 n20/D)	Sigma, USA	M5904
Nuclease-free water	-	Thermo, USA	AM9937
Pluronic acid F-68	-	Gibco, USA	24040032
Poly(4-styrenesulfonic acid)	~75000	Sigma, USA	561223
Poly(diallyldimethylammonium chloride)	(1.04 g/mL) (1.375 n20/D)	Sigma, USA	409014
POPC (1-palmitoyl-2-oleoyl-glycero-3-phosphocholine)	760.076	Avanti, USA	850457C
L-Glutamic acid potassium salt monohydrate	203.23	Sigma, USA	G1149
Potassium hydroxide	56.11	Sigma, USA	221473
Potassium phosphate dibasic solution	174.18	Sigma, USA	P8584
Potassium phosphate monobasic solution	136.086	Sigma, USA	P8709
Sodium chloride	58.44	Merck, USA	1.06404
Spermidine	145.25	Sigma, USA	S2626
Sucrose	342.30	Sigma, USA	S9378
SU-8 2025	-	Microchem, USA	-
TEMED (N,N,N',N'-Tetramethylethylenediamine)	116.21	Carl Roth, Germany	2367.3
Trizma base	121.14	Sigma, USA	T1503
Tris-Glycine-SDS buffer (10X)	-	Bio-rad, USA	1610772
Twinsil Speed silicone	-	Picodent, Germany	1300 1002
Urea	60.06	Merck, USA	108487
β-mercaptoethanol	78.13	Sigma, USA	M6250

*Benzonase was prepared in-house by the Protein Expression, Purification, and Characterization (PEPC) facility, Max Planck Institute of Molecular Cell Biology and Genetics (MPI-CBG).

Table S2. Kit list.

Name	Supplier	Catalog No.
HiScribe T7 High Yield RNA Synthesis Kit	NEB, USA	E2040
NEBuilder HiFi DNA Assembly Kit	NEB, USA	E2621
PURExpress In Vitro Protein Synthesis Kit	NEB, USA	E6800
QIAGEN Plasmid Maxi Kit	QIAGEN, Germany	12162
QIAGEN RNeasy Mini kit	QIAGEN, Germany	74104
QIAquick Gel Extraction Kit	QIAGEN, Germany	28704
QIAquick PCR Purification Kit	QIAGEN, Germany	28104
Quick Start Bradford Assay	Bio-Rad, USA	5000201

Table S3. Consumables list.

Name	Supplier	Catalog No.
24x60mm coverslip	Menzel-Gläser, Germany	-
384-well plates, Lobase, Black	Greiner Bio-One, Austria	788096
76x26x1mm microscope slides	Marienfeld, Germany	1000000
HisTrap FF column (5 ml)	Cytiva, USA	GE17-5255-01
Millex syringe filter (PVDF)	Millipore, USA	SLGV033RS
Adhesive PCR Plate Seals	Thermo, USA	AB0558
Spectra/Por 2 Dialysis Membrane (12-14 kD)	Repligen, USA	132680T
TGX Stain-Free PAGE Gel, 15-well, 4-15%	Bio-Rad, USA	4568086

Table S4. Equipment list.

Name	Supplier	Catalog No.
100x/1.3 Oil Plan-Neofluar Ph3 M27 objective	Zeiss, Germany	420491-9910-000
10X/0.45 Plan-Apochromat M27 objective	Zeiss, Germany	420640-9900-000
40X/1.2 C-Apochromat W autocorr M27 objective	Zeiss, Germany	421767-9971-790
5x/0.15 Plan-Neofluar Ph1 M27 objective	Zeiss, Germany	420331-9911-000
Andor Axiovert 200M	Zeiss, Germany	-
Avanti Centrifuge J26-XP	Beckman Coulter, USA	-
Biovision Gel Doc system	Biovision, USA	-
Dimax S4 Monochrome sCMOS high-speed camera	PCO, Germany	-
JLA-8.1000 rotor	Beckman Coulter, USA	363688
LSM 880 with Airyscan	Zeiss, Germany	-
Mitos P-Pump	Dolomite, UK	3200016
NanoDrop 2000	Thermo, USA	ND-2000
Osmomat 3000	Gonotec, Germany	-
pE-4000 illumination system	CoolLED, USA	-
Sonorex sonicator bath	Bandelin, Germany	-
Spark 20M plate reader	TECAN, Switzerland	-
Spin Coater WS-650MZ-23NPPB	Laurell Tech. Corp., USA	-
Typhoon 9500 Fluo and Phospho Imager	GE, USA	-

2. Plasmid construction

The strains and plasmids used in this study are listed in Table S5-6. Plasmids pEXP5-NT/6xHis eGFP [1] and pEXP5-NT/6xHis mCherry [2] were provided by JLR Anderson, University of Bristol. The pEXP5-NT/6xHis mCherry F30-2xdBroccoli was made by inserting the F30-2xdBroccoli [3,4] downstream of the mCherry stop codon and upstream of the terminator of the mCherry gene. The F30-2xdBroccoli indicates two dimeric Broccoli RNA aptamers supported by an F30 stem loop. All plasmids have a high-copy number origin of replication. To create the insert for pEXP5-NT/6xHis mCherry F30-2xdBroccoli, Broccoli RNA aptamer inserts were synthesized as ultramers with overlapping regions to the pEXP5-NT/6xHis mCherry vector plasmid. F30-2xdBroccoli ultramers 1 and 2 were annealed, PCR amplified, and column purified (QIAquick PCR Purification Kit) to obtain the dsDNA insert. The pEXP5-NT/6xHis mCherry plasmid vector was divided into two parts by PCR amplifying the template plasmid pEXP5-NT/6xHis mCherry using primers NFB ptetO gBlock Fwd and mCherry Rev (vector part 1) and Tphi Fwd and DUR Vector 2 (vector part 2). The PCR products were then digested with DpnI to remove the plasmid template then gel purified (QIAquick Gel Extraction Kit) to obtain the vector fragments only. Plasmid assembly of the purified DNA parts for pEXP5-NT/6xHis mCherry F30-2xdBroccoli plasmid was done using the NEBuilder HiFi DNA Assembly Kit and transformed into E. coli DH5α. All plasmids were prepared and purified by ethanol precipitation from E. coli DH5α cultures using the QIAGEN Plasmid Maxi Kit and measured by NanoDrop. Plasmid maps, sequences, and annotations are provided in Figs. S1-3, TableS S7-9, and Seqs. S1-3 (*and in GenBank format in the supplementary files*). Primer and ultramer sequences are provided in Tables S10-11. PCR cycling protocols are shown in Table S12. AGE results are provided in Fig. S4. All PCR, purification, and assembly methods were performed using the manufacturer's standard protocols. All primers and ultramers were synthesized by Integrated DNA Technologies (IDT, www.idtdna.com). Plasmid assembly was confirmed by Sanger sequencing (GENEWIZ, www.genewiz.com) using the primer pEXP5NT insert Rev. The pEXP5-NT/6xHis mCherry F30-2xdBroccoli plasmid is deposited in Addgene (www.addgene.org, plasmid ID 169233). Additionally, a plasmid with only one Broccoli aptamer downstream mCherry was also designed and deposited in Addgene (pEXP5-NT/6xHis mCherry F30-Broccoli, Addgene plasmid ID 169234).

Table S5. Strains used in this study.

Strain name	Description	Source
E. coli DH5α	High-efficiency competent cells for transformation.	NEB (C2987)
E. coli BL21 (DE3)	Competent cells for T7 RNAP-mediated expression.	NEB (C2527)

Table S6. Plasmids used in this study.

Plasmid	Description	Source
pEXP5-NT/6xHis eGFP	Constitutive T7 RNAP-mediated expression of 6xHis-eGFP.	[1]
pEXP5-NT/6xHis mCherry	Constitutive T7 RNAP-mediated expression of 6xHis-mCherry.	[2]
pEXP5-NT/6xHis mCherry F30-2xdBroccoli	Constitutive T7 RNAP-mediated expression of 6xHis-mCherry with 2xdBroccoli RNA aptamer.	This study.

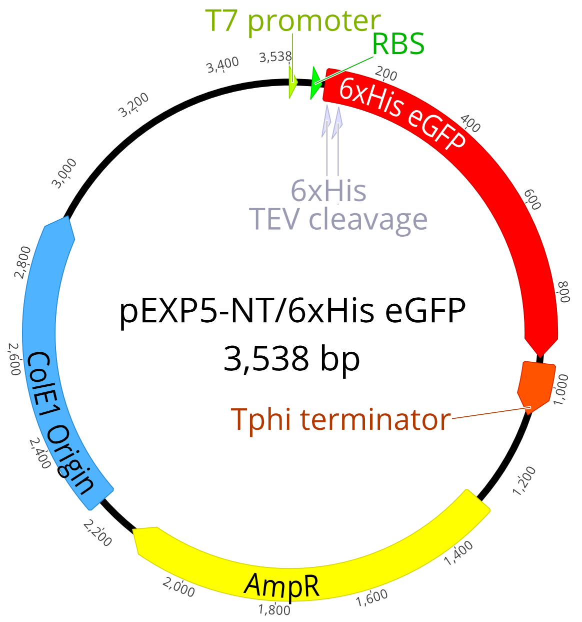

Figure S1. Plasmid map of pEXP5-NT/6xHis eGFP. The plasmid map illustration was created in Geneious 11.0.2 (www.geneious.com).

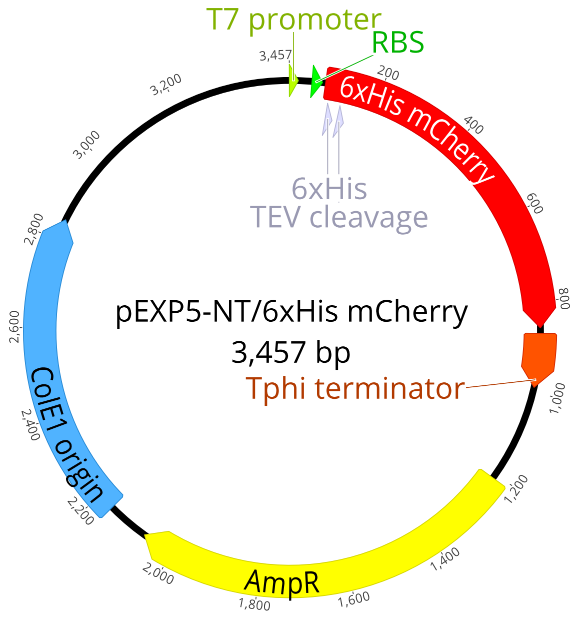

Figure S2. Plasmid map of pEXP5-NT/6xHis mCherry. The plasmid map illustration was created in Geneious 11.0.2 (www.geneious.com).

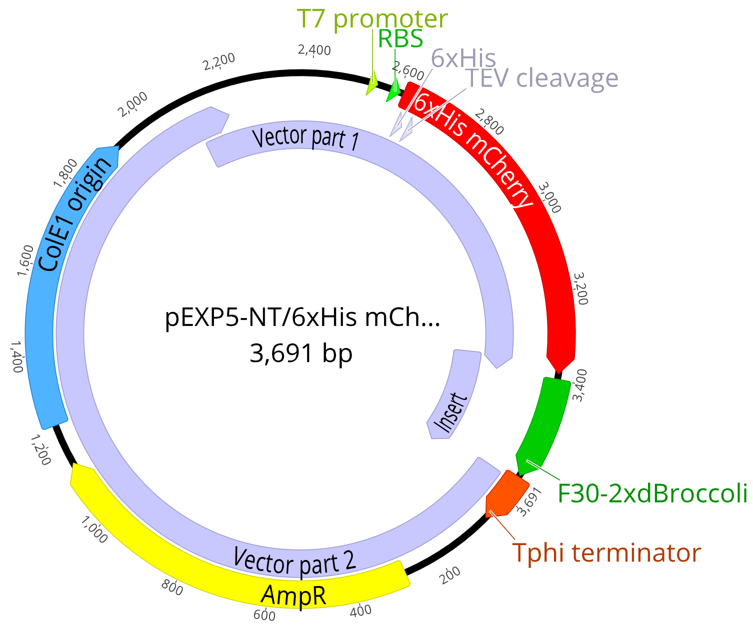

Figure S3. Plasmid map of pEXP5-NT/6xHis mCherry F30-2xdBroccoli. The DNA parts (vector parts 1-2 and insert) used to assemble the plasmid are annotated in light purple. The plasmid map illustration was created in Geneious 11.0.2 (www.geneious.com).

Table S7. Annotations of pEXP5-NT/6xHis eGFP plasmid.

Annotation	Start	End	Length (bp)
T7 promoter	3538	18	19
RBS	50	72	23
6xHis eGFP gene	79	939	861
6xHis	91	108	18
TEV cleavage	121	138	18
Tphi terminator	952	1070	119
AmpR gene	1291	2151	861
ColE1 Origin of replication	2246	2928	683

Table S8. Annotations of pEXP5-NT/6xHis mCherry plasmid.

Annotation	Start	End	Length (bp)
T7 promoter	3,457	18	19
RBS	50	72	23
6xHis mCherry gene	79	858	780
6xHis	91	108	18
TEV cleavage	121	138	18
Tphi terminator	871	989	119
AmpR gene	1210	2070	861
ColE1 Origin of replication	2165	2847	683

Table S9. Annotations of pEXP5-NT/6xHis mCherry F30-2xdBroccoli plasmid.

Annotation	Start	End	Length (bp)
T7 promoter	2531	2549	19
RBS	2581	2603	23
6xHis mCherry gene	2610	3386	777
6xHis	2622	2639	18
TEV cleavage	2652	2669	18
F30-2xdBroccoli aptamer	3399	3632	234
Tphi terminator	3636	63	119
AmpR gene	284	1144	861
ColE1 origin of replication	1239	1921	683
Vector part 1	2102	3397	1296
F30-2xdBroccoli Insert	3355	3691	337
Vector part 2	3650	2187	2229

Table S10. Primers used for cloning of the pEXP5-NT/6xHis mCherry F30-2xdBroccoli plasmid.

Primer	Sequence (5'-3')	Purpose
NFB ptetO gBlock Fwd	TTTCTCCTTACGCATCTGT	PCR of vector part 1
mCherry Rev	ATCACCCTTTAGCTGCC	
Tphi Fwd	CGAAAGGAAGCTGAGTTG	PCR of vector part 2
DUR vector 2	CTGGCTTAACTATGCGGC	
pEXP5NT insert Rev	GGTTATTGTCTCATGAGCG	Sanger sequencing

Table S11. Ultramers for inserts of the pEXP5-NT/6xHis mCherry F30-2xdBroccoli plasmid.

Primer	Sequence (5'-3')
F30-2xdBroccoli Ultramer 1	CCGGCGGCATGGACGAGCTGTACAAGGGCAGCTAAAGGGTGATC TTGCCATGTGTATGTGGGAGACGGTCGGGTCCATCTGAGACGGTC GGGTCCAGATATTCGTATCTGTCGAGTAGAGTGTGGGCTCAGATG TCGAGTAGAGTGTGGGCTCCCACATACTCTGATGATCCAGACGGT CGGGTCCATCTGA
F30-2xdBroccoli Ultramer 2	TTATTGCTCAGCGGTGGCAGCAGCCAACTCAGCTTCCTTTCGGGC TTTGTTAGCAGCCGTTGCCATGAATGATCCAGCCCACACTCTACT CGACATCTGAGCCCACACTCTACTCGACAGATACGAATATCTGGA CCCGACCGTCTCAGATGGACCCGACCGTCTGGATCATCA

Table S12. PCR protocol for amplification of plasmid parts.

PCR cycle	Temperature	Time
Initial denaturation	98 °C	40 secs
Denaturation	98 °C	10 secs
Annealing	53 °C	15 secs
Extension	72 °C	1.5/0.5 mins*
Final extension	72 °C	5 mins
Hold	4 °C	∞

*1.5 mins for vector parts 1-2 and 0.5 mins for insert.

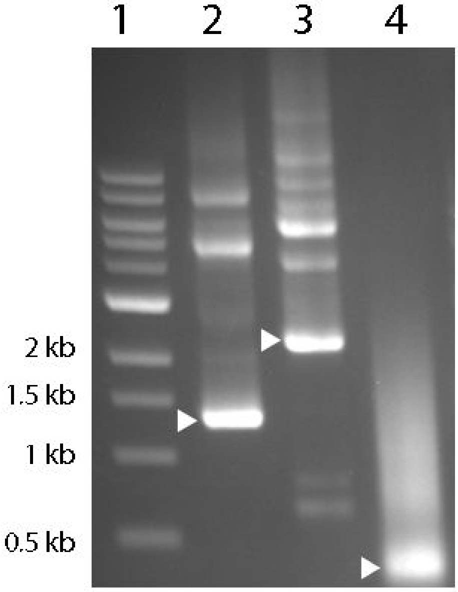

Figure S4. Agarose gel electrophoresis (1% agarose, 120 V, 30 mins, stained with GelRed) of DNA assembly parts for pEXP5-NT/6xHis mCherry F30-2xdBroccoli and pEXP5-NT/6xHis mCherry F30-Broccoli plasmids. Lane 1: 1 kb ladder, lane 2: PCR of vector part 1 (pT7-6xHis mCherry, 1296 bp), lane 3: PCR of vector part 2 (Tphi terminator-AmpR-ColE1 Ori, 2229 bp), and lane 4: PCR of insert (F30-2xdBroccoli, 337 bp. Target PCR products in lanes 2-4 are marked with a white arrow. Agarose gels were imaged using a Biovision Gel Doc system with 365 nm UV excitation.

Sequence S1. Plasmid sequence of pEXP5-NT/6xHis eGFP.
AATACGACTCACTATAGGGAGACCACAACGGTTTCCCTCTAGAAATAATTTTGTTTAACTTTAAGA
AGGAGATATACCATGTCTGGTTCTCATCATCATCATCATCATGGTAGCAGCGGCGAAAACCTGTAT
TTTCAGTCCCTTGTGGGTACCGGTTCGAATGATGACGACGACAAGAGTCCGGATCCCAATTGGATG
GTGAGCAAGGGCGAGGAGCTGTTCACCGGGGTGGTGCCCATCCTGGTCGAGCTGGACGGCGACGT
AAACGGCCACAAGTTCAGCGTGTCCGGCGAGGGCGAGGGCGATGCCACCTACGGCAAGCTGACCC
TGAAGTTCATCTGCACCACCGGCAAGCTGCCCGTGCCCTGGCCCACCCTCGTGACCACCCTGACCT
ACGGCGTGCAGTGCTTCAGCCGCTACCCCGACCACATGAAGCAGCACGACTTCTTCAAGTCCGCCA
TGCCCGAAGGCTACGTCCAGGAGCGCACCATCTTCTTCAAGGACGACGGCAACTACAAGACCCGC
GCCGAGGTGAAGTTCGAGGGCGACACCCTGGTGAACCGCATCGAGCTGAAGGGCATCGACTTCAA
GGAGGACGGCAACATCCTGGGGCACAAGCTGGAGTACAACTACAACAGCCACAACGTCTATATCA
TGGCCGACAAGCAGAAGAACGGCATCAAGGTGAACTTCAAGATCCGCCACAACATCGAGGACGGC
AGCGTGCAGCTCGCCGACCACTACCAGCAGAACACCCCCATCGGCGACGGCCCCGTGCTGCTGCCC
GACAACCACTACCTGAGCACCCAGTCCGCCCTGAGCAAAGACCCCAACGAGAAGCGCGATCACAT
GGTCCTGCTGGAGTTCGTGACCGCCGCCGGGATCACTCTCGGCATGGACGAGCTGTACAAGTCCGG
ACTCAGATCTCGAGTCTGGTAAAGGGTGATCCGGCTGCTAACAAAGCCCGAAAGGAAGCTGAGTT
GGCTGCTGCCACCGCTGAGCAATAACTAGCATAACCCCTTGGGGCCTCTAAACGGGTCTTGAGGGG
TTTTTTGCTGAAAGGAGGAACTATATCCGGATAATTCTTGAAGACGAAAGGGCCTCGTGATACGCC
TATTTTTATAGGTTAATGTCATGATAATAATGGTTTCTTAGACGTCAGGTGGCACTTTTCGGGGAAA
TGTGCGCGGAACCCCTATTTGTTTATTTTTCTAAATACATTCAAATATGTATCCGCTCATGAGACAA
TAACCCTGATAAATGCTTCAATAATATTGAAAAAGGAAGAGTATGAGTATTCAACATTTCCGTGTC
GCCCTTATTCCCTTTTTTGCGGCATTTTGCCTTCCTGTTTTTGCTCACCCAGAAACGCTGGTGAAAGT
AAAAGATGCTGAAGATCAGTTGGGTGCACGAGTGGGTTACATCGAACTGGATCTCAACAGCGGTA
AGATCCTTGAGAGTTTTCGCCCCGAAGAACGTTTTCCAATGATGAGCACTTTTAAAGTTCTGCTATG
TGGCGCGGTATTATCCCGTGTTGACGCCGGGCAAGAGCAACTCGGTCGCCGCATACACTATTCTCA
GAATGACTTGGTTGAGTACTCACCAGTCACAGAAAAGCATCTTACGGATGGCATGACAGTAAGAG
AATTATGCAGTGCTGCCATAACCATGAGTGATAACACTGCGGCCAACTTACTTCTGACAACGATCG
GAGGACCGAAGGAGCTAACCGCTTTTTTGCACAACATGGGGGATCATGTAACTCGCCTTGATCGTT
GGGAACCGGAGCTGAATGAAGCCATACCAAACGACGAGCGTGACACCACGATGCCTGTAGCAATG
GCAACAACGTTGCGCAAACTATTAACTGGCGAACTACTTACTCTAGCTTCCCGGCAACAATTAATA
GACTGGATGGAGGCGGATAAAGTTGCAGGACCACTTCTGCGCTCGGCCCTTCCGGCTGGCTGGTTT
ATTGCTGATAAATCTGGAGCCGGTGAGCGTGGGTCTCGCGGTATCATTGCAGCACTGGGGCCAGAT
GGTAAGCCCTCCCGTATCGTAGTTATCTACACGACGGGGAGTCAGGCAACTATGGATGAACGAAAT
AGACAGATCGCTGAGATAGGTGCCTCACTGATTAAGCATTGGTAACTGTCAGACCAAGTTTACTCA
TATATACTTTAGATTGATTTAAAACTTCATTTTTAATTTAAAAGGATCTAGGTGAAGATCCTTTTTG
ATAATCTCATGACCAAAATCCCTTAACGTGAGTTTTCGTTCCACTGAGCGTCAGACCCCGTAGAAA
AGATCAAAGGATCTTCTTGAGATCCTTTTTTTCTGCGCGTAATCTGCTGCTTGCAAACAAAAAAACC
ACCGCTACCAGCGGTGGTTTGTTTGCCGGATCAAGAGCTACCAACTCTTTTTCCGAAGGTAACTGG
CTTCAGCAGAGCGCAGATACCAAATACTGTTCTTCTAGTGTAGCCGTAGTTAGGCCACCACTTCAA
GAACTCTGTAGCACCGCCTACATACCTCGCTCTGCTAATCCTGTTACCAGTGGCTGCTGCCAGTGGC
GATAAGTCGTGTCTTACCGGGTTGGACTCAAGACGATAGTTACCGGATAAGGCGCAGCGGTCGGG
CTGAACGGGGGGTTCGTGCACACAGCCCAGCTTGGAGCGAACGACCTACACCGAACTGAGATACC
TACAGCGTGAGCTATGAGAAAGCGCCACGCTTCCCGAAGGGAGAAAGGCGGACAGGTATCCGGTA
AGCGGCAGGGTCGGAACAGGAGAGCGCACGAGGGAGCTTCCAGGGGGAAACGCCTGGTATCTTTA
TAGTCCTGTCGGGTTTCGCCACCTCTGACTTGAGCGTCGATTTTTGTGATGCTCGTCAGGGGGGCGG
AGCCTATGGAAAAACGCCAGCAACGCGGCCTTTTTACGGTTCCTGGCCTTTTGCTGGCCTTTTGCTC
ACATGTTCTTTCCTGCGTTATCCCCTGATTCTGTGGATAACCGTATTACCGCCTTTGAGTGAGCTGA
TACCGCTCGCCGCAGCCGAACGACCGAGCGCAGCGAGTCAGTGAGCGAGGAAGCGGAAGAGCGC
CTGATGCGGTATTTTCTCCTTACGCATCTGTGCGGTATTTCACACCGCAATGGTGCACTCTCAGTAC
AATCTGCTCTGATGCCGCATAGTTAAGCCAGTATACACTCCGCTATCGCTACGCTTCGTTAATACAG
ATGTAGGTGTTCCACAGGGTAGCCAGCAGCATCCTGCGATGCAGATCCGGAACATAATGGTGCAG
GGCGCTGACTTCCGCGTTTCCAGACTTTACGAAACACGGAAACCGAAGACCATTCATGTTGTTGCT
CAGGTCGCAGACGTTTTGCAGCAGCAGTCGCTTCACGTTCGCTCGCGTATCGGTGATTCATTCTGCT
AACCAGTAAGGCAACCCCGCCAGCCTAGCCGGGTCCTCAACGACAGGAGCACGATCATGCGCACC
CGTGGCCAGGACCCAACGCTGCCCGAGATCTCGATCCCGCGAAAT

Sequence S2. Plasmid sequence of pEXP5-NT/6xHis mCherry.
AATACGACTCACTATAGGGAGACCACAACGGTTTCCCTCTAGAAATAATTTTGTTTAACTTTAAGA
AGGAGATATACCATGTCTGGTTCTCATCATCATCATCATCATGGTAGCAGCGGCGAAAACCTGTAT
TTTCAGTCCATGGTGAGCAAGGGCGAGGAGGATAACATGGCCATCATCAAGGAGTTCATGCGCTTC
AAGGTGCACATGGAGGGCTCCGTGAACGGCCACGAGTTCGAGATCGAGGGCGAGGGCGAGGGCC
GCCCCTACGAGGGCACCCAGACCGCCAAGCTGAAGGTGACCAAGGGTGGCCCCCTGCCCTTCGCCT

GGGACATCCTGTCCCCTCAGTTCATGTACGGCTCCAAGGCCTACGTGAAGCACCCCGCCGACATCC
CCGACTACTTGAAGCTGTCCTTCCCCGAGGGCTTCAAGTGGGAGCGCGTGATGAACTTCGAGGACG
GCGGCGTGGTGACCGTGACCCAGGACTCCTCCCTGCAGGACGGCGAGTTCATCTACAAGGTGAAG
CTGCGCGGCACCAACTTCCCCTCCGACGGCCCCGTAATGCAGAAGAAGACCATGGGCTGGGAGGC
CTCCTCCGAGCGGATGTACCCCGAGGACGGCGCCCTGAAGGGCGAGATCAAGCAGAGGCTGAAGC
TGAAGGACGGCGGCCACTACGACGCTGAGGTCAAGACCACCTACAAGGCCAAGAAGCCCGTGCAG
CTGCCCGGCGCCTACAACGTCAACATCAAGTTGGACATCACCTCCCACAACGAGGACTACACCATC
GTGGAACAGTACGAACGCGCCGAGGGCCGCCACTCCACCGGCGGCATGGACGAGCTGTACAAGGG
CAGCTAAAGGGTGATCCGGCTGCTAACAAAGCCCGAAAGGAAGCTGAGTTGGCTGCTGCCACCGC
TGAGCAATAACTAGCATAACCCCTTGGGGCCTCTAAACGGGTCTTGAGGGGTTTTTTGCTGAAAGG
AGGAACTATATCCGGATAATTCTTGAAGACGAAAGGGCCTCGTGATACGCCTATTTTTATAGGTTA
ATGTCATGATAATAATGGTTTCTTAGACGTCAGGTGGCACTTTTCGGGGAAATGTGCGCGGAACCC
CTATTTGTTTATTTTTCTAAATACATTCAAATATGTATCCGCTCATGAGACAATAACCCTGATAAAT
GCTTCAATAATATTGAAAAAGGAAGAGTATGAGTATTCAACATTTCCGTGTCGCCCTTATTCCCTTT
TTTGCGGCATTTTGCCTTCCTGTTTTTGCTCACCCAGAAACGCTGGTGAAAGTAAAAGATGCTGAAG
ATCAGTTGGGTGCACGAGTGGGTTACATCGAACTGGATCTCAACAGCGGTAAGATCCTTGAGAGTT
TTCGCCCCGAAGAACGTTTTCCAATGATGAGCACTTTTAAAGTTCTGCTATGTGGCGCGGTATTATC
CCGTGTTGACGCCGGGCAAGAGCAACTCGGTCGCCGCATACACTATTCTCAGAATGACTTGGTTGA
GTACTCACCAGTCACAGAAAAGCATCTTACGGATGGCATGACAGTAAGAGAATTATGCAGTGCTG
CCATAACCATGAGTGATAACACTGCGGCCAACTTACTTCTGACAACGATCGGAGGACCGAAGGAG
CTAACCGCTTTTTTGCACAACATGGGGGATCATGTAACTCGCCTTGATCGTTGGGAACCGGAGCTG
AATGAAGCCATACCAAACGACGAGCGTGACACCACGATGCCTGTAGCAATGGCAACAACGTTGCG
CAAACTATTAACTGGCGAACTACTTACTCTAGCTTCCCGGCAACAATTAATAGACTGGATGGAGGC
GGATAAAGTTGCAGGACCACTTCTGCGCTCGGCCCTTCCGGCTGGCTGGTTTATTGCTGATAAATCT
GGAGCCGGTGAGCGTGGGTCTCGCGGTATCATTGCAGCACTGGGGCCAGATGGTAAGCCCTCCCGT
ATCGTAGTTATCTACACGACGGGGAGTCAGGCAACTATGGATGAACGAAATAGACAGATCGCTGA
GATAGGTGCCTCACTGATTAAGCATTGGTAACTGTCAGACCAAGTTTACTCATATATACTTTAGATT
GATTTAAAACTTCATTTTTAATTTAAAAGGATCTAGGTGAAGATCCTTTTTGATAATCTCATGACCA
AAATCCCTTAACGTGAGTTTTCGTTCCACTGAGCGTCAGACCCCGTAGAAAAGATCAAAGGATCTT
CTTGAGATCCTTTTTTTCTGCGCGTAATCTGCTGCTTGCAAACAAAAAAACCACCGCTACCAGCGGT
GGTTTGTTTGCCGGATCAAGAGCTACCAACTCTTTTTCCGAAGGTAACTGGCTTCAGCAGAGCGCA
GATACCAAATACTGTTCTTCTAGTGTAGCCGTAGTTAGGCCACCACTTCAAGAACTCTGTAGCACC
GCCTACATACCTCGCTCTGCTAATCCTGTTACCAGTGGCTGCTGCCAGTGGCGATAAGTCGTGTCTT
ACCGGGTTGGACTCAAGACGATAGTTACCGGATAAGGCGCAGCGGTCGGGCTGAACGGGGGGTTC
GTGCACACAGCCCAGCTTGGAGCGAACGACCTACACCGAACTGAGATACCTACAGCGTGAGCTAT
GAGAAAGCGCCACGCTTCCCGAAGGGAGAAAGGCGGACAGGTATCCGGTAAGCGGCAGGGTCGG
AACAGGAGAGCGCACGAGGGAGCTTCCAGGGGGAAACGCCTGGTATCTTTATAGTCCTGTCGGGT
TTCGCCACCTCTGACTTGAGCGTCGATTTTTGTGATGCTCGTCAGGGGGGCGGAGCCTATGGAAAA
ACGCCAGCAACGCGGCCTTTTTACGGTTCCTGGCCTTTTGCTGGCCTTTTGCTCACATGTTCTTTCCT
GCGTTATCCCCTGATTCTGTGGATAACCGTATTACCGCCTTTGAGTGAGCTGATACCGCTCGCCGCA
GCCGAACGACCGAGCGCAGCGAGTCAGTGAGCGAGGAAGCGGAAGAGCGCCTGATGCGGTATTTT
CTCCTTACGCATCTGTGCGGTATTTCACACCGCAATGGTGCACTCTCAGTACAATCTGCTCTGATGC
CGCATAGTTAAGCCAGTATACACTCCGCTATCGCTACGCTTCGTTAATACAGATGTAGGTGTTCCAC
AGGGTAGCCAGCAGCATCCTGCGATGCAGATCCGGAACATAATGGTGCAGGGCGCTGACTTCCGC
GTTTCCAGACTTTACGAAACACGGAAACCGAAGACCATTCATGTTGTTGCTCAGGTCGCAGACGTT
TTGCAGCAGCAGTCGCTTCACGTTCGCTCGCGTATCGGTGATTCATTCTGCTAACCAGTAAGGCAA
CCCCGCCAGCCTAGCCGGGTCCTCAACGACAGGAGCACGATCATGCGCACCCGTGGCCAGGACCC
AACGCTGCCCGAGATCTCGATCCCGCGAAAT

Sequence S3. Plasmid sequence of pEXP5-NT/6xHis mCherry F30-2xdBroccoli.
CTAGCATAACCCCTTGGGGCCTCTAAACGGGTCTTGAGGGGTTTTTTGCTGAAAGGAGGAACTATA
TCCGGATAATTCTTGAAGACGAAAGGGCCTCGTGATACGCCTATTTTTATAGGTTAATGTCATGATA
ATAATGGTTTCTTAGACGTCAGGTGGCACTTTTCGGGGAAATGTGCGCGGAACCCCTATTTGTTTAT
TTTTCTAAATACATTCAAATATGTATCCGCTCATGAGACAATAACCCTGATAAATGCTTCAATAATA
TTGAAAAAGGAAGAGTATGAGTATTCAACATTTCCGTGTCGCCCTTATTCCCTTTTTTGCGGCATTT
TGCCTTCCTGTTTTTGCTCACCCAGAAACGCTGGTGAAAGTAAAAGATGCTGAAGATCAGTTGGGT
GCACGAGTGGGTTACATCGAACTGGATCTCAACAGCGGTAAGATCCTTGAGAGTTTTCGCCCCGAA
GAACGTTTTCCAATGATGAGCACTTTTAAAGTTCTGCTATGTGGCGCGGTATTATCCCGTGTTGACG
CCGGGCAAGAGCAACTCGGTCGCCGCATACACTATTCTCAGAATGACTTGGTTGAGTACTCACCAG
TCACAGAAAAGCATCTTACGGATGGCATGACAGTAAGAGAATTATGCAGTGCTGCCATAACCATG
AGTGATAACACTGCGGCCAACTTACTTCTGACAACGATCGGAGGACCGAAGGAGCTAACCGCTTTT
TTGCACAACATGGGGGATCATGTAACTCGCCTTGATCGTTGGGAACCGGAGCTGAATGAAGCCATA

CCAAACGACGAGCGTGACACCACGATGCCTGTAGCAATGGCAACAACGTTGCGCAAACTATTAAC
TGGCGAACTACTTACTCTAGCTTCCCGGCAACAATTAATAGACTGGATGGAGGCGGATAAAGTTGC
AGGACCACTTCTGCGCTCGGCCCTTCCGGCTGGCTGGTTTATTGCTGATAAATCTGGAGCCGGTGA
GCGTGGGTCTCGCGGTATCATTGCAGCACTGGGGCCAGATGGTAAGCCCTCCCGTATCGTAGTTAT
CTACACGACGGGGAGTCAGGCAACTATGGATGAACGAAATAGACAGATCGCTGAGATAGGTGCCT
CACTGATTAAGCATTGGTAACTGTCAGACCAAGTTTACTCATATATACTTTAGATTGATTTAAAACT
TCATTTTTAATTTAAAAGGATCTAGGTGAAGATCCTTTTTGATAATCTCATGACCAAAATCCCTTAA
CGTGAGTTTTCGTTCCACTGAGCGTCAGACCCCGTAGAAAAGATCAAAGGATCTTCTTGAGATCCT
TTTTTTCTGCGCGTAATCTGCTGCTTGCAAACAAAAAAACCACCGCTACCAGCGGTGGTTTGTTTGC
CGGATCAAGAGCTACCAACTCTTTTTCCGAAGGTAACTGGCTTCAGCAGAGCGCAGATACCAAATA
CTGTTCTTCTAGTGTAGCCGTAGTTAGGCCACCACTTCAAGAACTCTGTAGCACCGCCTACATACCT
CGCTCTGCTAATCCTGTTACCAGTGGCTGCTGCCAGTGGCGATAAGTCGTGTCTTACCGGGTTGGAC
TCAAGACGATAGTTACCGGATAAGGCGCAGCGGTCGGGCTGAACGGGGGGTTCGTGCACACAGCC
CAGCTTGGAGCGAACGACCTACACCGAACTGAGATACCTACAGCGTGAGCTATGAGAAAGCGCCA
CGCTTCCCGAAGGGAGAAAGGCGGACAGGTATCCGGTAAGCGGCAGGGTCGGAACAGGAGAGCG
CACGAGGGAGCTTCCAGGGGGAAACGCCTGGTATCTTTATAGTCCTGTCGGGTTTCGCCACCTCTG
ACTTGAGCGTCGATTTTTGTGATGCTCGTCAGGGGGGCGGAGCCTATGGAAAAACGCCAGCAACGC
GGCCTTTTTACGGTTCCTGGCCTTTTGCTGGCCTTTTGCTCACATGTTCTTTCCTGCGTTATCCCCTG
ATTCTGTGGATAACCGTATTACCGCCTTTGAGTGAGCTGATACCGCTCGCCGCAGCCGAACGACCG
AGCGCAGCGAGTCAGTGAGCGAGGAAGCGGAAGAGCGCCTGATGCGGTATTTTCTCCTTACGCAT
CTGTGCGGTATTTCACACCGCAATGGTGCACTCTCAGTACAATCTGCTCTGATGCCGCATAGTTAAG
CCAGTATACACTCCGCTATCGCTACGCTTCGTTAATACAGATGTAGGTGTTCCACAGGGTAGCCAG
CAGCATCCTGCGATGCAGATCCGGAACATAATGGTGCAGGGCGCTGACTTCCGCGTTTCCAGACTT
TACGAAACACGGAAACCGAAGACCATTCATGTTGTTGCTCAGGTCGCAGACGTTTTGCAGCAGCAG
TCGCTTCACGTTCGCTCGCGTATCGGTGATTCATTCTGCTAACCAGTAAGGCAACCCCGCCAGCCTA
GCCGGGTCCTCAACGACAGGAGCACGATCATGCGCACCCGTGGCCAGGACCCAACGCTGCCCGAG
ATCTCGATCCCGCGAAATAATACGACTCACTATAGGGAGACCACAACGGTTTCCCTCTAGAAATAA
TTTTGTTTAACTTTAAGAAGGAGATATACCATGTCTGGTTCTCATCATCATCATCATCATGGTAGCA
GCGGCGAAAACCTGTATTTTCAGTCCATGGTGAGCAAGGGCGAGGAGGATAACATGGCCATCATC
AAGGAGTTCATGCGCTTCAAGGTGCACATGGAGGGCTCCGTGAACGGCCACGAGTTCGAGATCGA
GGGCGAGGGCGAGGGCCGCCCCTACGAGGGCACCCAGACCGCCAAGCTGAAGGTGACCAAGGGT
GGCCCCCTGCCCTTCGCCTGGGACATCCTGTCCCCTCAGTTCATGTACGGCTCCAAGGCCTACGTGA
AGCACCCCGCCGACATCCCCGACTACTTGAAGCTGTCCTTCCCCGAGGGCTTCAAGTGGGAGCGCG
TGATGAACTTCGAGGACGGCGGCGTGGTGACCGTGACCCAGGACTCCTCCCTGCAGGACGGCGAG
TTCATCTACAAGGTGAAGCTGCGCGGCACCAACTTCCCCTCCGACGGCCCCGTAATGCAGAAGAAG
ACCATGGGCTGGGAGGCCTCCTCCGAGCGGATGTACCCCGAGGACGGCGCCCTGAAGGGCGAGAT
CAAGCAGAGGCTGAAGCTGAAGGACGGCGGCCACTACGACGCTGAGGTCAAGACCACCTACAAGG
CCAAGAAGCCCGTGCAGCTGCCCGGCGCCTACAACGTCAACATCAAGTTGGACATCACCTCCCACA
ACGAGGACTACACCATCGTGGAACAGTACGAACGCGCCGAGGGCCGCCACTCCACCGGCGGCATG
GACGAGCTGTACAAGGGCAGCTAAAGGGTGATCTTGCCATGTGTATGTGGGAGACGGTCGGGTCC
ATCTGAGACGGTCGGGTCCAGATATTCGTATCTGTCGAGTAGAGTGTGGGCTCAGATGTCGAGTAG
AGTGTGGGCTCCCACATACTCTGATGATCCAGACGGTCGGGTCCATCTGAGACGGTCGGGTCCAGA
TATTCGTATCTGTCGAGTAGAGTGTGGGCTCAGATGTCGAGTAGAGTGTGGGCTGGATCATTCATG
GCAACGGCTGCTAACAAAGCCCGAAAGGAAGCTGAGTTGGCTGCTGCCACCGCTGAGCAATAA

3. mRNA expression and purification

The mRNA transcript of the pEXP5-NT/6xHis mCherry F30-2xdBroccoli plasmid containing the mCherry gene and Broccoli RNA aptamer was transcribed *in vitro* and purified for RNA calibration and RNA titration experiments. To prepare the DNA template for *in vitro* transcription (IVT), the region containing the T7 promoter up to the terminator of the pEXP5-NT/6xHis mCherry F30-2xdBroccoli plasmid was PCR amplified (Phusion HF) using primers pEXP5-NT Insert Fwd and Rev (Tables S13-14), digested with DpnI (NEB, 20 units/50ul PCR reaction volume) to remove the plasmid template, and purified (QIAquick PCR Purification Kit) (Fig. S5A). The purified PCR product was used as the template for IVT using the HiScribe T7 High Yield RNA Synthesis Kit which was used according to manufacturer's instructions with 5 hours incubation at 37 °C and DNAse I treatment. The IVT reaction product is then purified using the QIAGEN RNeasy kit as described by the manufacturer. The purified mRNA was confirmed by Nanodrop 260/280 ratios of ~2.0, native agarose gel, and denaturing urea-PAGE (Fig. S5B-C). We observed two separate bands in the denaturing urea polyacrylamide gel for the 6xHis mCherry F30-2xdBroccoli RNA transcript. To check if these two bands still contain the Broccoli aptamer, an in-gel DFHBI staining was performed according to [4], which showed that both bands fluoresce with DFHBI (Fig S5D). Therefore, both fractions had the complete RNA aptamer sequences. The two bands in the denaturing RNA gel could be a result of transcription read-through over the terminator of the PCR amplicon DNA template. However, we assumed that both species contained the F30-2xdBroccoli RNA aptamer because the DNA template is from a pure single band PCR product, both RNA bands are only slightly longer than the expected size (~1224 bp), and both bands are fluorescent with the DFHBI from the in-gel staining. Therefore, using this purified mRNA for our calibration curves is consistent on a molar concentration basis. Purified mRNA was stored at -20 °C until further use.

Table S13. Primers used to amplify IVT DNA template.

Primer	Sequence (5'-3')
pEXP5-NT Insert Fwd	TCGGTGATTCATTCTGCT
pEXP5-NT Insert Rev	GGTTATTGTCTCATGAGCG

Table S14. PCR protocol for amplification of IVT DNA template.

PCR cycle	Temperature	Time
Initial denaturation	98 °C	40 secs
Denaturation	98 °C	10 secs
Annealing	53 °C	15 secs
Extension	72 °C	45 secs
Final extension	72 °C	5 mins
Hold	10 °C	∞

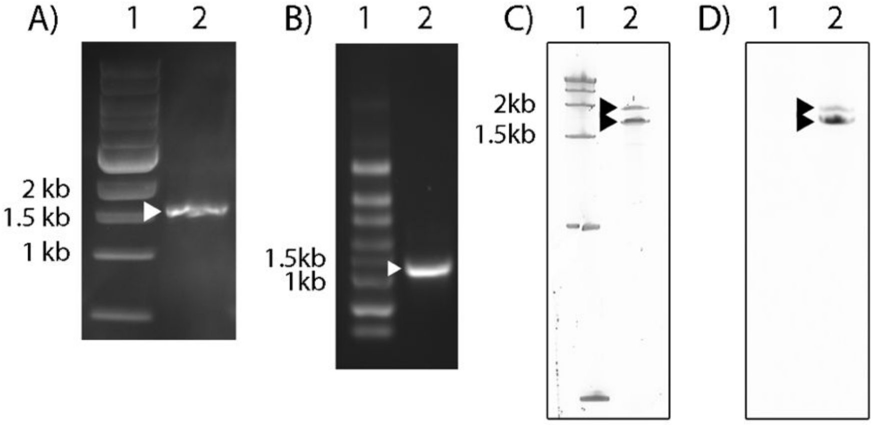

Figure S5. *In vitro* transcription and purification of mRNA. (A) AGE of DNA template for IVT reaction amplified from pEXP5-NT/6xHis mCherry F30-2xdBroccoli plasmid using primers pEXP5NT Insert Fwd and Rev (2% agarose, 150 V, 30 mins, stained with GelRed). Lane 1: 1 kb ladder, lane 2: PCR product (1534 bp). (B) Purified RNA from IVT reaction in a native agarose gel (1 % agarose, 120 V, 40 mins, stained with GelRed). Lane 1: Riboruler High Range RNA ladder, lanes 2-4: dilutions of purified RNA transcript (expected size ~1224bp). (C) Purified RNA from IVT reaction in a denaturing urea polyacrylamide gel (3% acrylamide, 8 M urea, 200 V, 45 mins, stained with SYBRgold). Lane 1: Riboruler High Range RNA ladder, lane 2: purified RNA transcript showing two separate bands (black arrows). (D) In-gel staining of purified RNA from IVT reaction in a denaturing urea polyacrylamide gel. After running the gel (3% acrylamide, 8 M urea, 200 V, 45 mins), the gel was washed

3x with water for 5 minutes and then stained with 10 μM DFHBI, 40 mM HEPES (pH 7.4), 100 mM KCl, and 1 mM $MgCl_2$ for 30 mins before imaging. Lane 1: Riboruler High Range RNA ladder, lane 2: purified RNA transcript showing two DFHBI stained bands (black arrows). Agarose gels were imaged using a Biovision Gel Doc system with 365 nm UV excitation. Polyacrylamide gels were imaged using a Typhoon 9500 Fluo and Phospho Imager (BPB1-530DF20 filter).

4. Protein expression and purification

pEXP5-NT/6xHis eGFP and pEXP5-NT/6xHis mCherry plasmids were transformed into *E. coli* BL21 (DE3) strains and used for protein expression and purification of eGFP and mCherry 6xHis-tagged proteins for quantitative calibration. All culture media was composed of LB with 100 µg/ml Ampicillin. For both mCherry and eGFP protein expression, overnight cultures were used to inoculate 500 ml of production cultures with 40µM IPTG to a starting OD_{600} of 0.015. After incubation at 37 °C and stirring at 180 rpm for approximately 4 hours (OD_{600}~5), the cultures were centrifuged at 5000 rpm (Avanti Centrifuge J26-XP with JLA-8.1000 rotor, Beckman Coulter) for 5 minutes at 4 °C to pellet the cells. The media was removed and the cells resuspended in lysis buffer (50mL lysis buffer/L culture) (Table S15). The resuspended cells were lysed by high-pressure homogenization by running the cell dispersion through the Emulsiflex C5-Avestin at 1500 bar two times. The lysed extracts were then clarified by centrifugation at 16000 rpm for 30 minutes and filtered through a 0.45 µm membrane (Millex-HV Low Protein Binding Durapore Membrane (PVDF), 33 mm diameter filter unit, 0.45 µm pore size) to collect only the soluble protein fraction. Ni-Sepharose resin HisTrap columns (HisTrap FF 5 ml, Cytiva) were washed with water and pre-equilibriated with 25 ml of the same lysis buffer. The clarified extracts containing the His-tagged proteins were loaded on the column, washed with 25 ml lysis buffer, 25 ml high salt buffer, and 25 ml lysis buffer at a rate of 5 ml/min. The proteins were eluted from the column with elution buffer (Table S15) at 2 ml/min. The protein was collected at 18 fractions at 2 ml each. A Bradford assay (Bio-Rad) was run on each of the 18 fractions to determine which fraction from the column contained the His-tagged protein to collect. The selected fractions were placed in a 12-14 kDa cut-off dialysis membrane (Spectra/Por 2 Dialysis Membrane Standard RC Tubing MWCO: 12-14 kDa) and dialyzed at 4 °C overnight in storage buffer (Table S15). After dialysis, aliquots of the purified proteins were flash frozen in liquid nitrogen and stored at -80 °C until use. All media and buffer recipes are listed in Table S15. Purified proteins were confirmed by size using SDS or Native PAGE (Bio-Rad TGX Stain-Free PAGE Gel, 15-well, 4-15%) and absorbance spectra using UV-vis scanning (Nanodrop) (Fig. S6). To load the gels, 5 µl of sample was mixed with 5 µl of 2X Laemmli buffer, incubated at 95 °C for 5 mins, and then 7 µl was loaded onto the gel (Bio-Rad TGX Stain-Free PAGE Gel, 15-well, 4-15%). Samples were run at 200 V for 30 mins in 1X Tris-Glycine-sodium dodecyl sulfate (SDS) or 1X Tris-Glycine buffer.

Table S15. Media and buffer recipes for protein expression and purification.

Buffer	Components	Concentration
LB media	NaCl	10 g/L
	Tryptone	10 g/L
	Yeast extract	5 g/L
	Ampicillin	100 µg/ml
Lysis buffer	PBS	2X
	Imidazole	20 mM
	DTT	1 mM
	Benzonase	0.025 mg/L
	Protease inhibitor	1 tablet/L
	$MgCl_2$	1 mM
	pH (+HCl/NaOH)	7.5
HiSalt buffer	PBS	6X
	pH (+HCl/NaOH)	7.5
Elution buffer	PBS	2X
	DTT	0.3 mM
	Imidazole	250 mM
Storage buffer	TrisHCl	25 mM
	NaCl	150 mM
	Glycerol	5%
	pH (+HCl/NaOH)	8
1X PBS buffer	NaCl	137 mM
	KCl	2.7 mM
	Na_2HPO_4	10 mM
	KH_2PO_4	2 mM
	pH (+HCl/NaOH)	7.5

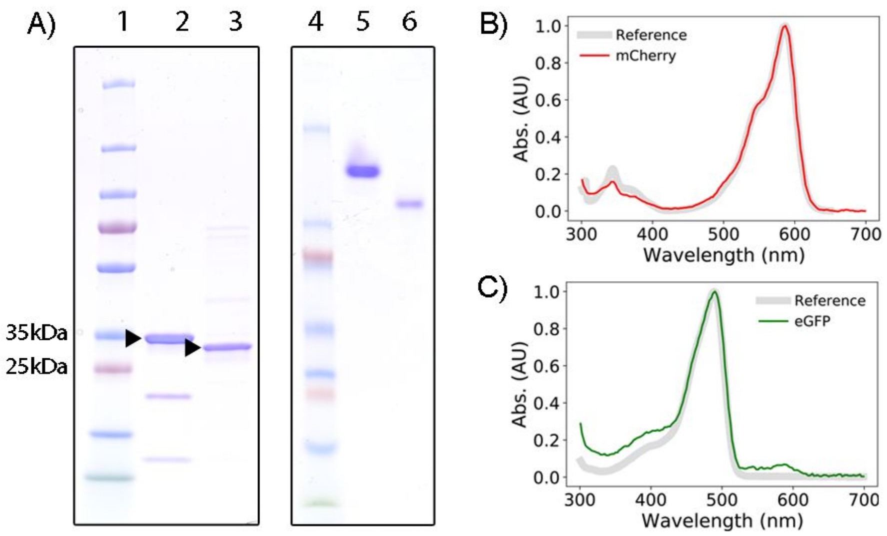

Figure S6. Purified mCherry and eGFP proteins. (A) SDS PAGE of purified proteins. Lane 1 - PageRuler Plus protein ladder (Thermo, USA), lane 2 - purified mCherry (29.222 kDa), and lane 3 - purified eGFP (32.153 kDa). Low molecular weight bands (~20 kDa and ~12 kDa) visible in the mCherry lanes are self-cleaved products of mCherry as a result of sample boiling prior to SDS PAGE [5]. Target protein products in lanes 2 and 3 are marked with a black arrow. Lanes 4-6 are native non-denaturing gels of the same samples that show single bands of the proteins. Lane 1 - PageRuler Plus protein ladder (Thermo, USA), lane 2 - purified mCherry (29.222 kDa), and lane 3 - purified eGFP (32.153 kDa). Purified 6xHis mCherry (B) and 6xHis eGFP (C) absorbance spectra compared to its reference spectra (www.fpbase.org/protein/egfp/ and www.fpbase.org/protein/mCherry/).

5. Protein and mRNA calibration

To obtain calibration curved for eGFP and mCherry, purified stock solutions of eGFP and mCherry proteins were quantified by Bradford assay (Quick Start Bradford Assay, Bio-Rad) with a BSA protein standard and NanoDrop A280 readings. The purified 6xHis mCherry F30-2xdBroccoli mRNA stock solution was quantified using NanoDrop RNA-40 readings. Mean values obtained from the Bradford assay were used for downstream calculations for protein concentrations and calibration (Table S16). Serial dilutions of protein and mRNA stock solutions were prepared with a PURExpress master mix solution (Table S18) without plasmid DNA or mRNA, with and without DFHBI to ensure equivalent conditions between the gene expression reaction mixture and the calibration samples required for correct calibrations. These calibration standards were then loaded in a 384-well plate (12.5 µl/well) or loaded on a glass slide (2 µl/spot) (Fig. S7). Glass slides comprised of a parafilm border sandwiched by a glass slide (76x26x1 mm microscope slides, Marienfeld) and coverslip (24x60 mm, Menzel-Gläser). Linear calibration curves to convert relative fluorescence units (RFU) to concentration units (nM) were obtained for eGFP, mCherry, and mRNA F30-2xdBroccoli aptamer in both the plate reader (Figs. S8-9) and confocal microscopy (Figs. S10-11) at the same acquisition settings for bulk and liposome-encapsulated experiments. Histograms of RFU per pixel in the ROIs of the calibration standards (Figs. S8B and S9B) show increase of fluorescence distributions as a function of increasing standard concentration. Fluorescence sampling uses the mean of the circular ROIs in Fig. S36 across the microscopy images and calculates standard deviations from these mean ROI values (Figs. S8A and S9A). Higher laser intensities were avoided to prevent bleaching effects of the CFES. Our methodologies provide an accurate method for quantification of mRNA and protein concentrations within the synthetic cells from confocal imaging. It should be noted that we used an 8-bit format for our image acquisition settings, which provides RFU values ranging from 0-256. In this instance, this imaging format was sufficient to provide enough differentiation for calibration of different standard concentrations. If better differentiation was required between signal values then a 16-bit imaging format can be used to provide finer range RFU values from 0 to 65535. Excitation/emission wavelengths in the TECAN Spark 20M plate reader were 485/535 nm (Gain 50), 570/620 nm (Gain 70), and 450/510 nm (Gain 60) with a bandwidth of ±20 nm each, for eGFP, mCherry, and Broccoli RNA, respectively. For confocal microscopy (LSM 880 with Airyscan, 10X/0.45 Plan-Apochromat M27 objective), excitation/detection wavelengths are 488 nm/499-561 nm for RNA Broccoli and eGFP protein, and 561 nm/579-64 1nm for mCherry protein. As the 488 nm laser power gradually decreased between experiments, the mRNA F30-2xdBroccoli aptamer calibration curve used for DNA titration in synthetic cell populations experiment was interpolated between two calibration curves based on the measured laser power (Fig. S10A). Image analysis and collection of the RFU values from confocal images for calibration are further discussed in Section 13.

Table S16. eGFP protein, mCherry protein, and 6xHis mCherry F30-2xdBroccoli mRNA stock concentrations. Protein concentrations were measured by Bradford assay and A280 measurements on the Nanodrop (in parentheses). mRNA concentration was measured by NanoDrop RNA-40 readings.

Stock	µM	A260/280	A260/230
eGFP protein	79.34 ± 9.47 (79.93)	-	-
mCherry protein	226.74 ± 10.63 (284.72)	-	-
6xHis mCherry F30-2xdBroccoli mRNA	26.42	2	2.34

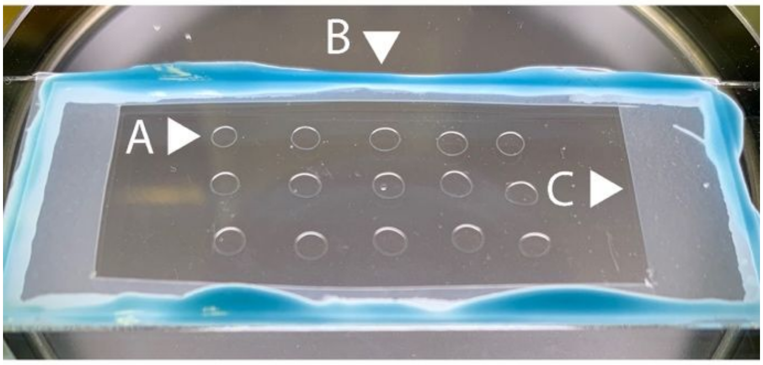

Figure S7. Glass slide setup for confocal microscopy calibration standards. (A) 2 µl spots of standard solutions for calibration. (B) Twinsil Speed silicone sealing the glass slide and cover slip setup. (C) Parafilm layer between glass slide and coverslip.

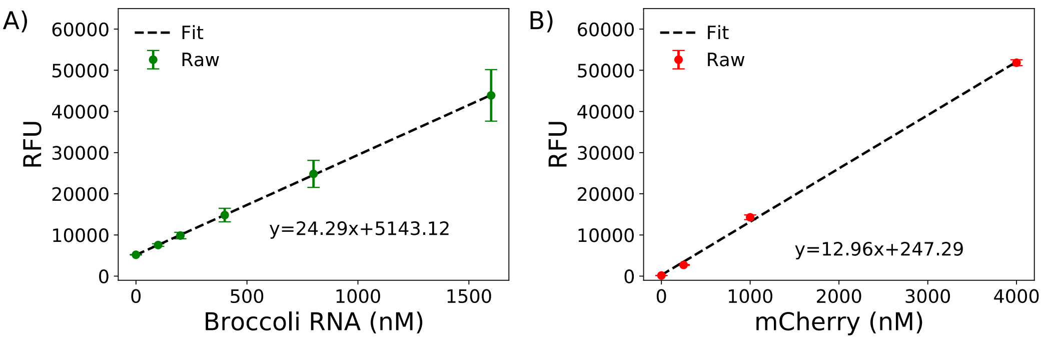

Figure S8. Linear calibration curve of (A) F30-2xdBroccoli RNA aptamer and (B) mCherry protein in PURExpress master mix with 10 µM DFHBI in a bulk plate reader format. Mean and standard deviation bars are calculated from triplicate samples.

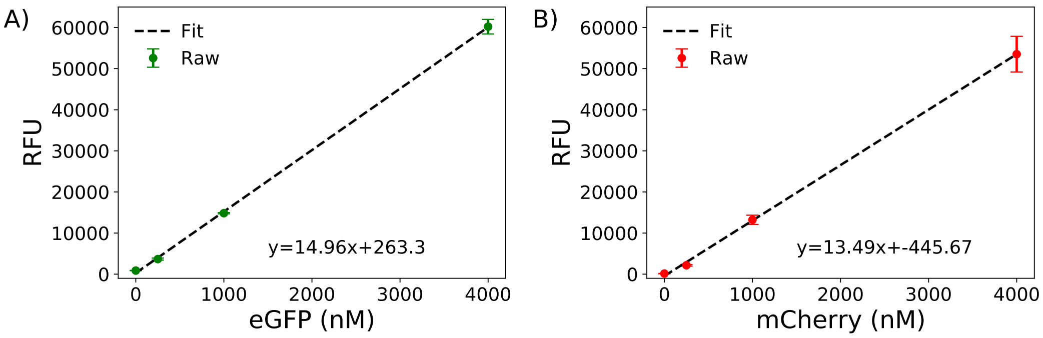

Figure S9. Linear calibration curve of (A) eGFP protein and (B) mCherry protein in PURExpress mastermix without DFHBI in a bulk plate reader format. Mean and standard deviation bars are calculated from triplicate samples.

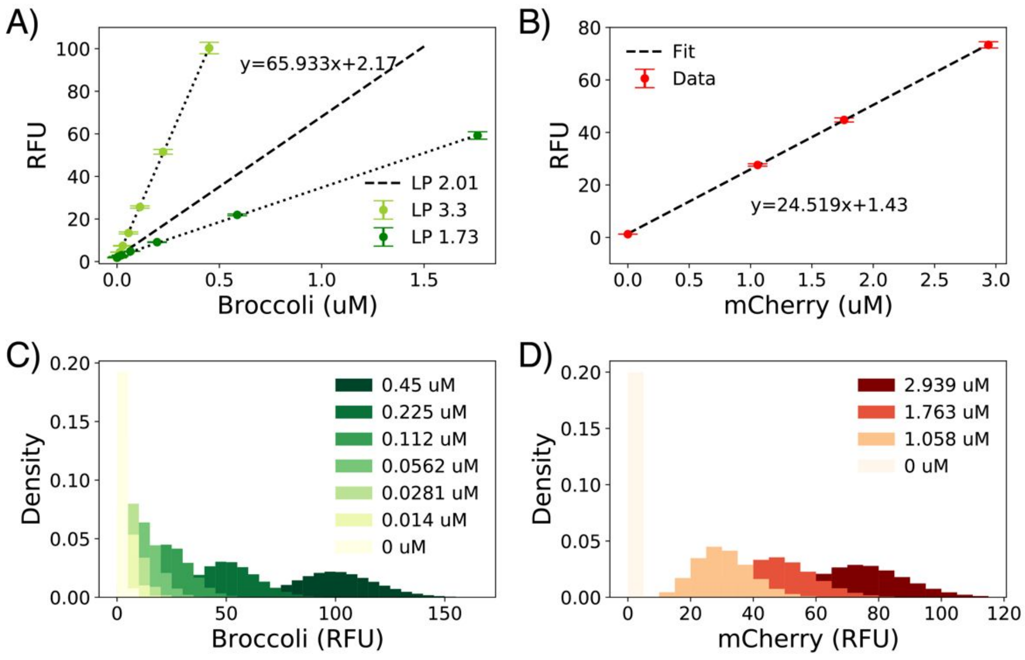

Figure S10. Calibration curves of (A) F30-2xdBroccoli RNA aptamer and (B) mCherry protein in PURExpress master mix with 10 uM DFHBI obtained from confocal images. Dotted lines in (A) are F30-2xdBroccoli RNA aptamer calibration curves at laser powers 3.3 mW (light green) and 1.73 mW (green). The dashed line is the linear interpolated calibration curve at laser power 2.01 mW used for experiments described in Section 14. The calibration curve at 1.73 mW was used for experiments described in Section 15. Mean and standard deviation bars are calculated from the mean values of ROIs (Fig. S37). RFU distributions per pixel in the ROIs for (C) F30-2xdBroccoli RNA aptamer (LP 3.3) and (D) mCherry protein calibration standards.

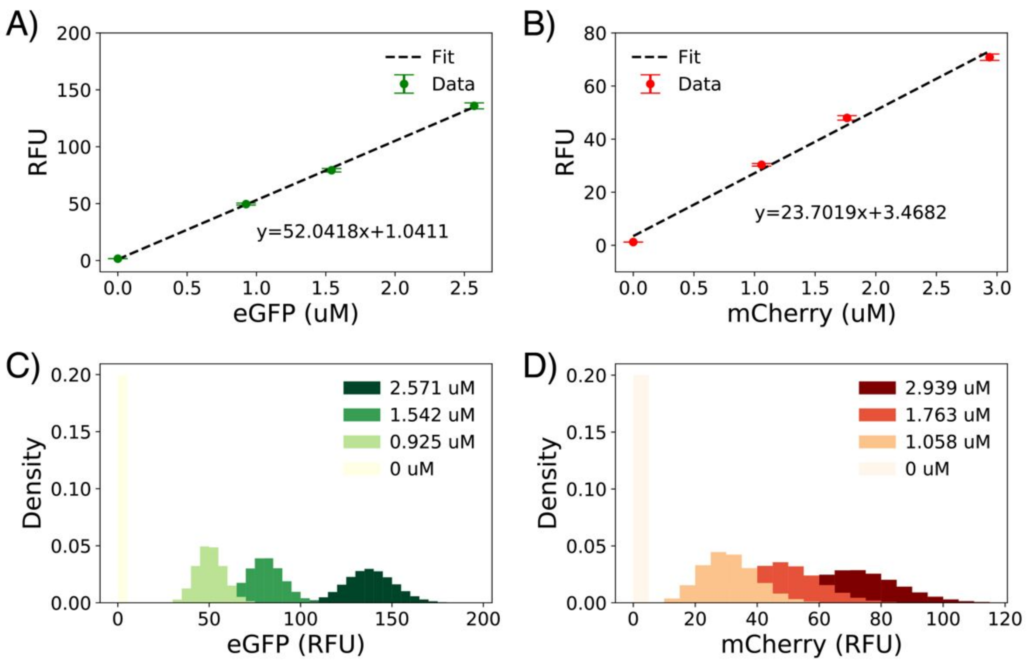

Figure S11. Calibration curve of (A) eGFP protein and (B) mCherry protein in PURExpress mastermix without DFHBI obtained from optical confocal images. Mean and standard deviations are calculated from the mean values of ROIs (Fig. S37). RFU distributions per pixel obtained from the ROIs for (C) eGFP protein and (D) mCherry protein calibration standards show differentiation between the fluorescence intensities of the standard proteins.

Table S17. Laser powers for quantified experiments.

Experiment	Laser (nm)	Power (mW)	Section
RNA Broccoli calibration 1	488	3.3	S6
	561	3.96	
	633	1.61	
eGFP and mCherry protein calibration	488	1.73	S6
	561	3.22	
	633	1.26	
RNA Broccoli calibration 2	488	1.73	S6
	561	3.22	
	633	1.26	
DNA titration in synthetic cell populations	488	2.01	S14
	561	3.16	
	633	1.12	
Two plasmids in a synthetic population	488	1.73	S15
	561	3.22	
	633	1.26	

6. Bulk cell-free expression experiments

Bulk cell free expression experiments were undertaken using standard half-volume (12.5 µL) reactions of the standard PURExpress *In Vitro* Protein Synthesis Kit. Reactions were prepared according to Table S18 for each sample, loaded into 384-well plates, sealed with an adhesive PCR plate clear film (Thermo, USA), and incubated in the TECAN Spark 20M plate reader at 30 °C for 8 hours. Fluorescence readings for the Broccoli RNA aptamer and mCherry protein, or eGFP protein and mCherry protein, were taken every 10 minutes with linear shaking of 5 s, 1 mm amplitude, and 1440 rpm frequency at each cycle. Excitation/emission wavelengths were 485/535 nm (Gain 50), 570/620 nm (Gain 70), and 450/510 nm (Gain 60) with a bandwidth of ±20 nm for eGFP, mCherry, and Broccoli RNA, respectively. DNA plasmid and mRNA templates were prepared by serial dilution with nuclease-free water before adding into the CFES reaction for the DNA (Fig. S12) and RNA titration experiments (Fig. S13). Transcription and translation rates from timeseries data are calculated using rate averages from a rolling window of 1 hour and plotted in Figs. S14-15.

Table S18. PURExpress master mix for 1X reaction

Component	Volume (µl)
Solution A	5
Solution B	3.75
RNAse Inhibitor	0.25
Sucrose (1.5 M)	0.67
DFHBI (500 µM)*	0.25
Plasmid DNA or mRNA	x
Water	fill to 12.5

*Omitted if using plasmids without F30-2xdBroccoli RNA aptamer.

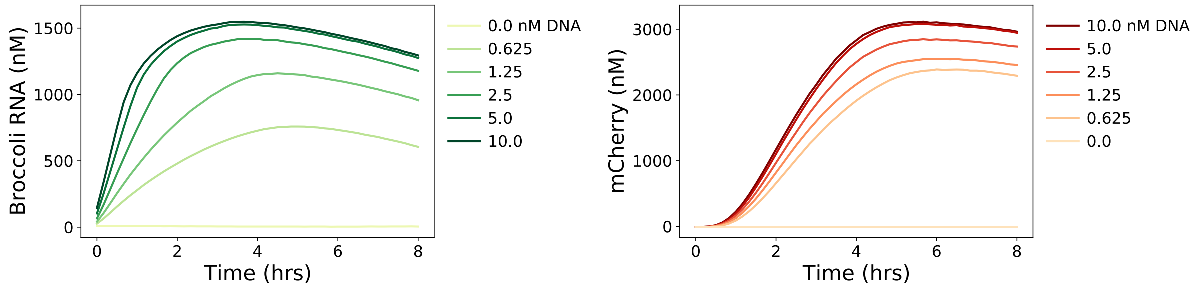

Figure S12. Timelapse of RNA Broccoli and mCherry protein expression in bulk PURExpress CFES at different pEXP5-NT/6xHis mCherry F30-2xdBroccoli plasmid DNA concentrations.

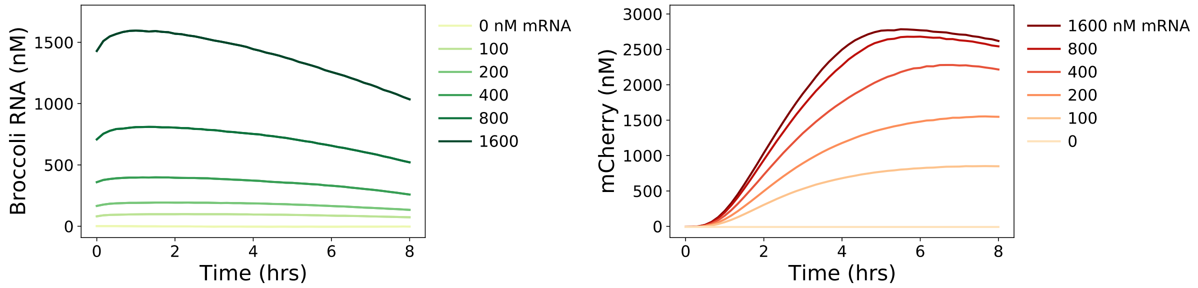

Figure S13. Timelapse of RNA Broccoli and mCherry protein expression in bulk PURExpress CFES at different 6xHis mCherry F30-2xdBroccoli transcript mRNA concentrations.

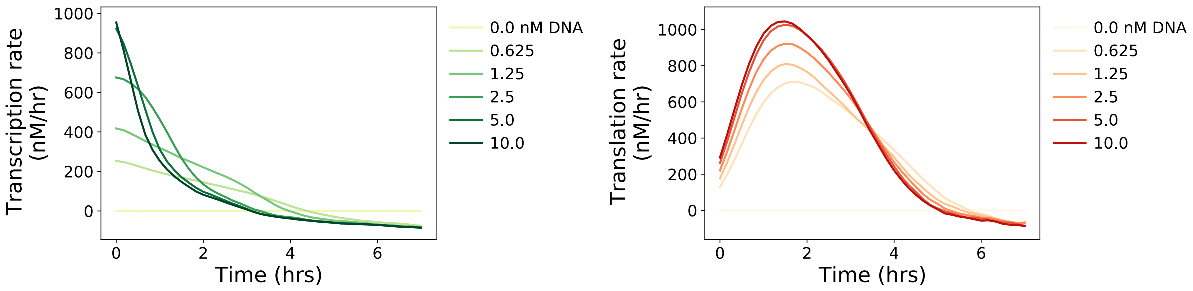

Figure S14. Transcription and translation rates from bulk PURExpress CFES at different pEXP5-NT/6xHis mCherry F30-2xdBroccoli plasmid DNA concentrations. Rates are calculated from results in Fig. S12 using rate averages with a rolling window of 1 hour.

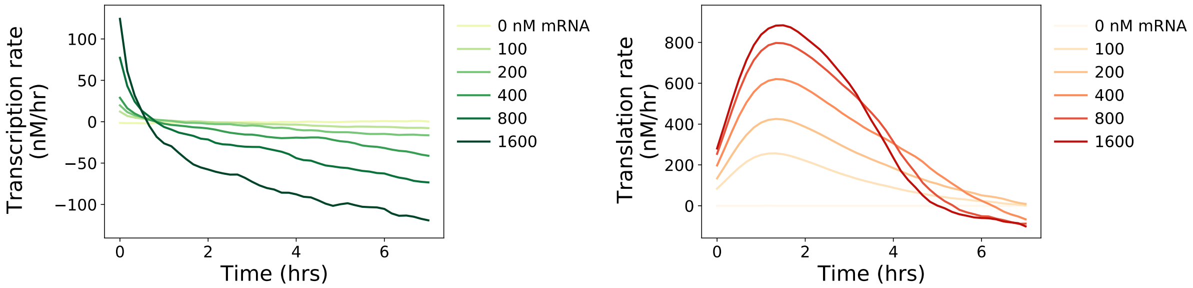

Figure S15. Transcription and translation rates from bulk PURExpress CFES at different 6xHis mCherry F30-2xdBroccoli transcript mRNA concentrations. Rates are calculated from results in Fig. S13 using rate averages with a rolling window of 1 hour.

7. mCherry maturation

Maturation rate of the mCherry in PURExpress was measured using a modified RNAseA assay previously described in [6]. A PURExpress reaction was prepared with DFHBI (see Table S18) and 300 nM 6xHis mCherry F30-2xdBroccoli mRNA template. The reaction was incubated at 30 °C for 30 mins to allow the CFES to produce mCherry protein. After 30 mins, 300 nM of RNAseA was added into the CFES reaction to degrade all mRNA and halt translation. mRNA Broccoli aptamer and mCherry protein fluorescence values were monitored every 10 minutes in a TECAN Spark 20M plate reader while incubated at 30°C with linear shaking of 5 s, 1 mm amplitude, and 1440 rpm frequency at each cycle (Fig. S16). Excitation/emission wavelengths used were 570/620 nm (Gain 70) and 450/510 nm (Gain 60) with a bandwidth of ±20 nm each, for mCherry and Broccoli RNA, respectively. The maturation rate of mCherry was measured from the increase of mCherry signal after the RNA signal was depleted (~2 hours after the addition of RNAseA). The increase of mCherry signal is attributed to the maturation of the protein and fit to a first order kinetic model (Eqns. S1-2, Fig. S17). The mCherry maturation rate parameter obtained from the RNAseA assay is $k_{mat} = 2.15 \pm 0.12\ \mathrm{hr}^{-1}$.

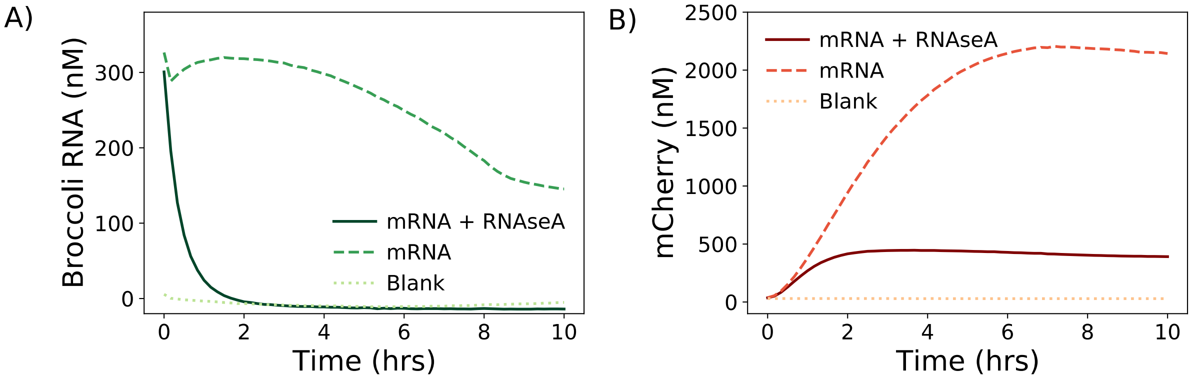

Figure S16. RNAseA assay for mCherry maturation rate. After 30 mins incubation of PURExpress with 6xHis mCherry F30-2xdBroccoli mRNA template, 300 nM RNAseA was added to deplete mRNA and halt translation. Broccoli RNA aptamer (A) and mCherry protein (B) levels were monitored over time. Treatments without mRNA template and without RNAseA addition were included as controls. All samples were done in triplicate. Solid lines indicate mean values and shaded regions are within standard deviations. Time measured in the x-axis is upon addition of RNAseA.

$$\frac{d[Protein^*]}{dt} = k_{mat}[Protein] \qquad \text{(S1)}$$

$$[Protein^*] = [Protein^*]_0 + [Protein]_0(1 - e^{-k_{mat}t}) \qquad \text{(S2)}$$

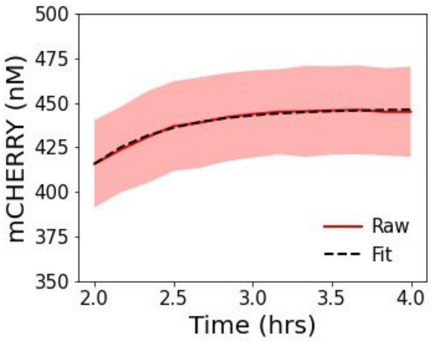

Figure S17. Fit of the mCherry signal to a first order kinetic model (Eqn. S2) for protein maturation from 2-4 hours after addition of RNAseA. Fit parameters are $k_{mat} = 2.15 \pm 0.12\ \mathrm{hr}^{-1}$ and $[Protein]_0 = 30.72 \pm 0.47$ nM

.

8. Resource-limited gene expression models

Seven different candidate models for cell-free gene expression (summarized in Table S19), based on and including the resource-limited gene expression model in [7], were used to fit our bulk and encapsulated RNA Broccoli and mCherry protein expression timeseries data. The general structure of the models consists of either mass-action, Michaelis-Menten, or Hill kinetics to describe the different steps of transcription and translation to provide a coarse-grained model of gene expression. In addition, gene expression was modeled as a resource-limited process. Transcription and translation processes consume TsR and TlR species, which are transcription and translation resources, respectively. To illustrate, the resource-limited gene expression model can be described by the reaction network with their respective reaction rate constants in Eqns. S3-10.

$$DNA + RNAP \overset{k_{f1}/k_{b1}}{\longleftrightarrow} DNA{:}RNAP \tag{S3}$$

$$DNA{:}RNAP + TsR \overset{k_{cat1}}{\longrightarrow} DNA + RNAP + RNA \tag{S4}$$

$$RNA + Rb \overset{k_{f2}/k_{b2}}{\longleftrightarrow} RNA{:}Rb \tag{S5}$$

$$RNA{:}Rb + TlR \overset{k_{cat2}}{\longrightarrow} RNA + Rb + Protein \tag{S6}$$

$$Protein \overset{k_{mat}}{\longrightarrow} Protein^* \tag{S7}$$

$$RNA \overset{\delta_{RNA}}{\longrightarrow} \tag{S8}$$

$$TsR \overset{\delta_{TsR}}{\longrightarrow} \tag{S9}$$

$$TlR \overset{\delta_{TlR}}{\longrightarrow} \tag{S10}$$

For transcription, DNA is first reversibly bound to RNA polymerase ($RNAP$) to form a $DNA{:}RNAP$ complex (Eqn. S3). This complex can consume transcription resources, TsR, to produce RNA (Eqn. S4). In the process, DNA and $RNAP$ dissociate from each other. Following mass-action kinetics, the rate equation for RNA production can be written as

$$\frac{d[RNA]}{dt} = k_{cat1}[DNA{:}RNAP][TsR]. \tag{S11}$$

Assuming that the binding/unbinding of DNA and $RNAP$ is fast and in quasi-equilibrium

$$k_{f1}[DNA][RNAP] = k_{b1}[DNA{:}RNAP]. \tag{S12}$$

Additionally, assuming that total $RNAP$ concentration is conserved,

$$k_{f1}[DNA]\big([RNAP]_0 - [DNA{:}RNAP]\big) = k_{b1}[DNA{:}RNAP] \tag{S13}$$

$$[DNA{:}RNAP] = \frac{k_{f1}[DNA][RNAP]_0}{k_{b1} + k_{f1}[DNA]} \tag{S14}$$

where $[RNAP]_0$ is the initial amount of RNA polymerase in the reaction. Replacing $[DNA{:}RNAP]$ in the rate equation of RNA production and grouping constants together results in

$$\frac{d[RNA]}{dt} = \frac{k_{cat1}k_{f1}[DNA][RNAP]_0[TsR]}{k_{b1} + k_{f1}[DNA]} \tag{S15}$$

$$\frac{d[RNA]}{dt} = \frac{k_r[DNA][TsR]}{K_r + [DNA]} \tag{S16}$$

where $k_r = k_{cat1}[RNAP]_0$ is an effective rate constant for transcription and $K_r = {}^{k_{b1}}/_{k_{f1}}$ is the dissociation constant between DNA and $RNAP$. A similar derivation can be performed for translation of protein (Eqns. S5-6) to obtain

$$\frac{d[Protein]}{dt} = \frac{k_p[Protein][TlR]}{K_p + [Protein]} \tag{S17}$$

where k_p is an effective rate constant for translation and K_p is the dissociation constant between RNA and ribosome, Rb. The remaining steps for protein maturation, RNA degradation (Eqn. S8), TsR degradation (Eqn.

S9), and TlR degradation (Eqn. S10) in the reaction network are modeled as first order reactions. Altogether, the reaction network of Eqns. S3-10 can be written as a system of ordinary differential equations (Eqns. S18-23)

$$\frac{d[DNA]}{dt} = 0 \tag{S18}$$

$$\frac{d[RNA]}{dt} = \frac{k_r TsR[DNA]}{K_r + [DNA]} - \delta_r[RNA] \tag{S19}$$

$$\frac{d[Protein]}{dt} = \frac{k_p TlR[RNA]}{K_p + [RNA]} - k_{mat}[Protein] \tag{S20}$$

$$\frac{d[Protein^*]}{dt} = k_{mat}[Protein] \tag{S21}$$

$$\frac{dTsR}{dt} = -\frac{ak_r TsR[DNA]}{K_r + [DNA]} - \delta_{TsR}TsR \tag{S22}$$

$$\frac{dTlR}{dt} = -\frac{bk_p TlR[RNA]}{K_p + [RNA]} - \delta_{TlR}TlR \tag{S23}$$

where TsR and TlR are assigned unitless quantities starting from 1 and are consumed during transcription and translation, scaled by the factors a and b, respectively. Delay differential equations can be used to describe translation to account for the ~ 0.5 hour delay of protein expression observed in mRNA titration bulk experiments. For example, Eqn. 20 can be rewritten to include a time-delay, τ_d, as

$$\frac{d[Protein]}{dt} = \frac{k_p TlR[RNA](t-\tau_d)}{K_p + [RNA](t-\tau_d)} - k_{mat}[Protein] \tag{S24}$$

where $[RNA](t-\tau_d)$ is the RNA abundance at time $(t-\tau_d)$.

Different combinations of mass-action or Michaelis-Menten kinetics are used for the degradation of TsR and TlR in the candidate models. However, unlike the Michaelis-Menten kinetics of transcription and translation, we do not derive TsR and TlR degradation kinetics from a particular reaction network because TsR and TlR are abstracted species of transcription and translation resources. We chose to represent their degradation dynamics as either first order or Michaelis-Menten kinetics for simplicity. For example, model 1 is shown in Eqns. S25-30, which includes Michaelis-Menten type kinetics for resource degradation (2nd terms on Eqns. S29 and S30). Model 7 is the model provided by Stögbauer et al. (2012)[7] without a delay differential equation for translation, while model 5 includes a delay differential equation for the translation step. Each parameter is described in Table S20.

$$\frac{d[DNA]}{dt} = 0 \tag{S25}$$

$$\frac{d[RNA]}{dt} = \frac{k_r TsR[DNA]}{K_r + [DNA]} - \delta_r[RNA] \tag{S26}$$

$$\frac{d[Protein]}{dt} = \frac{k_p TlR[RNA](t-\tau_d)}{K_p + [RNA](t-\tau_d)} - k_{mat}[Protein] \tag{S27}$$

$$\frac{d[Protein^*]}{dt} = k_{mat}[Protein] \tag{S28}$$

$$\frac{dTsR}{dt} = -\frac{ak_r TsR[DNA]}{K_r + [DNA]} - \frac{\delta_{TsR}TsR}{K_s + TsR} \tag{S29}$$

$$\frac{dTlR}{dt} = -\frac{bk_p TlR[RNA](t-\tau_d)}{K_p + [RNA](t-\tau_d)} - \frac{\delta_{TlR}TlR}{K_l + TlR} \tag{S30}$$

Table S19. Summary of seven candidate CFES models. Each model is composed of the different reaction steps modeled by mass-action kinetics (MA), Michaelis-Menten kinetics (MM), or Michaelis-Menten kinetics with delay (DM). Model 7 is the model provided in Stögbauer et al. (2012) [7].

**Step**	**Model 1**	**Model 2**	**Model 3**	**Model 4**	**Model 5**	**Model 6**	**Model 7**
Transcription	MM	MM	MM	MM	MM	MM	MM
Translation	DM	DM	DM	DM	DM	DM	MM
RNA degradation	MA	MA	MA	MA	MA	MA	MA
Protein maturation	MA	MA	MA	MA	MA	MA	MA
TsR consumption	MM	MM	MM	MM	MM	MM	MM
TlR consumption	DM	DM	DM	-	-	DM	-
TsR degradation	MM	MA	MM	MM	-	-	-
TlR degradation	MM	MM	MA	-	MM	-	MM

Table S20. Rate parameters of resource-limited gene expression models.

**Parameter**	**Units**	**Description**
k_r	nM/hr	RNA transcription rate.
K_r	nM	Dissociation constant between $RNAP$ and DNA.
δ_r	1/hr	Degradation rate of RNA.
k_p	nM/hr	Protein translation rate.
K_p	nM	Dissociation constant between ribosome and RNA.
k_{mat}	1/hr	Protein maturation rate.
δ_{TsR}	1/hr	TsR degradation rate.
K_s	-	Michaelis-Menten constant of TsR degradation.
δ_{TlR}	1/hr	TlR degradation rate.
K_l	-	Michaelis-Menten constant of TlR degradation.
a	-	Scaling factor for consumption of TsR with transcription.
b	-	Scaling factor for consumption of TlR with translation.
τ_d	hr	Time-delay of translation.

9. Parameter estimation, model selection, and profile likelihoods

All methods for parameter estimation, model selection, and profile likelihood analysis were written in Julia (v1.5.3) [8] using the packages DifferentialEquations, Distributions, Optim, Plots, DataFrames, DelimitedFiles, and CSV.

Parameter estimation

We obtained parameter estimates of each model by maximizing the agreement between the experimental data and model. This agreement can be quantified by the likelihood $p(D \mid \theta)$, defined as the probability of observing the experimental data D given the model parameters $\theta = \{\theta_1,\cdots,\theta_k\}$. The specific form of the likelihood $p(D \mid \theta)$ results from the considered measurement noise model. Throughout this work, we consider both Broccoli RNA and mCherry measurements to be corrupted by independent Gaussian measurement noise with standard deviation σ_r and σ_p, respectively. The rate parameters of a model were estimated by minimizing the negative logarithm of the likelihood

$$\hat{\theta} = \mathrm{argmin}_{\theta}[-\ln p(D \mid \theta)] \tag{S31}$$

where $\hat{\theta}$ is referred to as the maximum-likelihood estimator of the model parameters θ. All rate parameters in each model were estimated simultaneously in the log domain to avoid negative parameter values. In addition to the rate parameters, the unknown standard deviations for Broccoli RNA and mCherry protein mean fluorescence signal (σ_r and σ_p) and the time lag between reaction start and data collection (τ_d) were also estimated from the data. The rate parameter for mCherry maturation (k_{mat}) was determined from independent experiments described in Section 8. The negative logarithm of the likelihood was minimized using a Nelder-Mead algorithm where ODE or DDE models were solved using a Bogacki-Shampine Runge-Kutta method. Initial parameters used are reported in the supplementary files and were taken from pre-search runs to facilitate better convergence of the optimization procedure.

AIC model selection

To rank the seven candidate models, we calculated the Akaike information criterion (AIC) defined as

$$\mathrm{AIC} = 2k - 2\ln p(D \mid \hat{\theta}) \tag{S32}$$

where k is the number of estimated parameters and $\ln p(D \mid \hat{\theta})$ is the log-likelihood evaluated at the optimal set of parameters $\hat{\theta}$. The AIC values of each model are shown in Table S21. Fits of the models with bulk experiments are shown in Figs. S18-24. Model 7 is the same model provided by Stögbauer et al. (2012) [7], but resulted in the highest AIC (and lowest log-likelihood). This was due to the initial delay of mCherry signal in both DNA and RNA titration experiments and endpoints of mRNA in the DNA titration experiments not fitting well. We then modified this model to include a delay in translation (models 1-6) and degradation of transcription resources (models 1-4) to account for the observed deviations from experimental data. These model modifications improved the AIC (Table S21) and qualitative fit (Figs. S18-24). Overall, model 2 was selected to be presented in the main text because it had the lowest AIC score, which accounts for both goodness-of-fit through the log-likelihood score, as well as model complexity by a penalty term that increases with the number of unknown model parameters.

Profile likelihoods and confidence intervals

Profile likelihoods for models were calculated according to [9,10] to determine identifiability and confidence intervals of each parameter. The profile likelihood of a parameter is calculated by

$$\mathrm{PL}(\theta_i) = \min_{\theta_{j \neq i}} [-\ln p(D \mid \theta)] \tag{S33}$$

which is the minimum of the negative log-likelihood with respect to all parameters $\theta_{j \neq i}$ while holding the parameter θ_i fixed [9]. Likelihood-based confidence intervals were estimated as the regions with

$$\{\theta_i | \mathrm{PL}(\theta_i) + \ln p(D \mid \hat{\theta}) < \chi^2(\alpha,df)\} \tag{S34}$$

where $\chi^2(\alpha,df)$ is the chi-squared distribution with $\alpha = 0.95$ confidence level and df degrees of freedom, which here is the number of parameters. Graphically, this means that the confidence intervals are found at the intersections of the profile likelihood and the significance threshold horizontal line at $-\ln p(D \mid \hat{\theta}) + \chi^2(\alpha,df)$. Profile likelihoods of each parameter between a range greater and less than $\hat{\theta}$ by a factor of 10 at 11 equally spaced increments in of models 1-7 are shown in Figs. S25-30. The 95% likelihood-based confidence intervals of the

parameters from the profile likelihoods (*Table 1 in the main text)* are calculated from the upper and lower intersection of the profile likelihood and the significance threshold line. Structural non-identifiable parameters are indicated by flat profile likelihoods below the significance threshold in both -∞/+∞ directions. Practically non-identifiable parameters are indicated by profile likelihoods below the significance threshold in both or either -∞/+∞ directions, but still have a unique minimum [9].

Table S21. Log-likelihood and AIC of the different CFES models based on data from bulk experiments.

Model	Log-likelihood	Parameters	AIC
**1**	-6822.78	15	13675.56
**2**	-6822.87	14	13673.74
**3**	-6887.60	14	13803.21
**4**	-6922.83	14	13873.65
**5**	-7255.11	12	14534.21
**6**	-7208.64	11	14439.28
**7**	-7354.29	11	14730.58

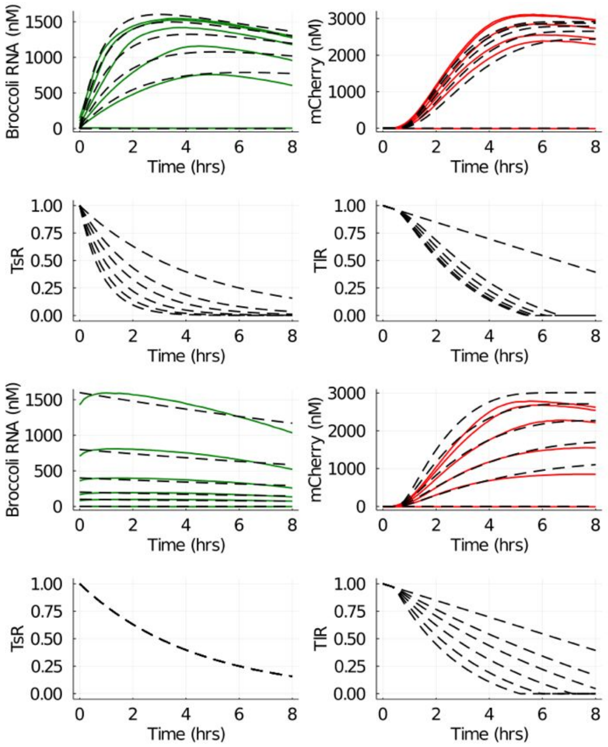

Figure S18. Model 1 fit with DNA titration and RNA titration bulk experiments. Colored lines are mean timeseries experimental data for the different species RNA, $Protein$, TsR, and TlR. Dashed lines are the model simulation from the parameter estimates.

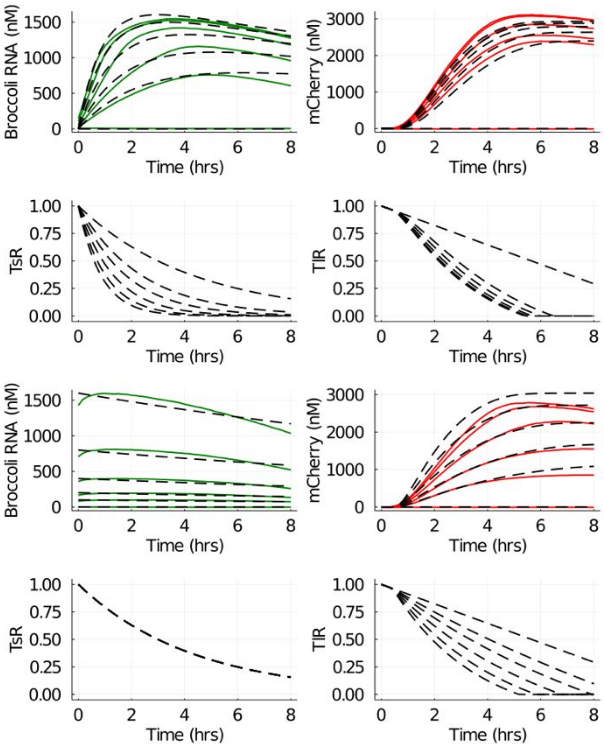

Figure S19. Model 2 fit with DNA titration and RNA titration bulk experiments. Colored lines are mean timeseries experimental data for the different species RNA, $Protein$, TsR, and TlR. Dashed lines are the model simulation from the parameter estimates.

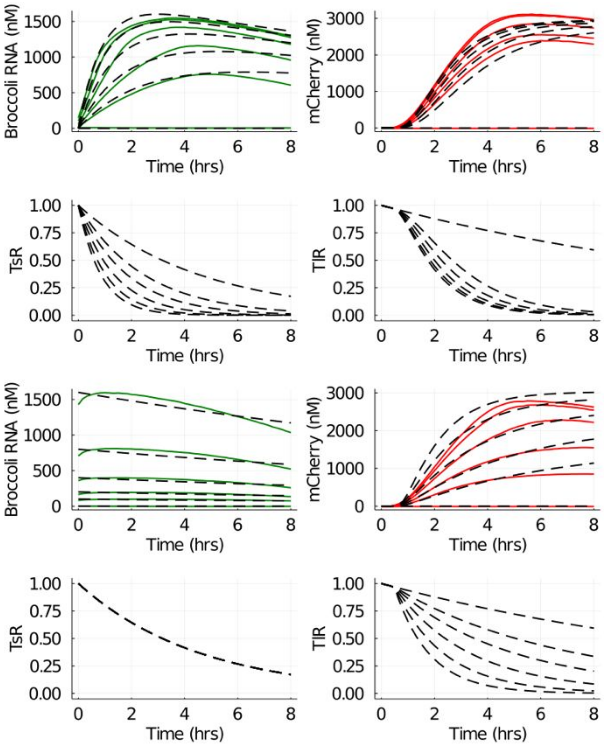

Figure S20. Model 3 fit with DNA titration and RNA titration bulk experiments. Colored lines are mean timeseries experimental data for the different species RNA, $Protein$, TsR, and TlR. Dashed lines are the model simulation from the parameter estimates.

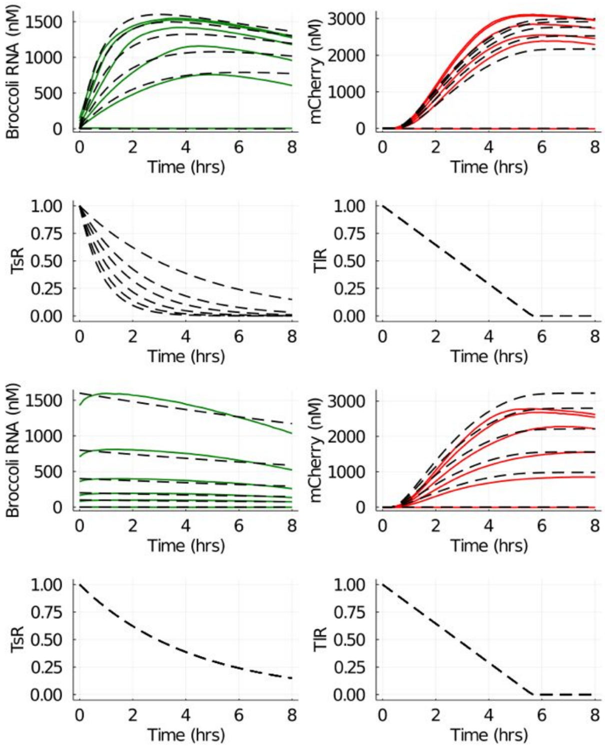

Figure S21. Model 4 fit with DNA titration and RNA titration bulk experiments. Colored lines are mean timeseries experimental data for the different species RNA, $Protein$, TsR, and TlR. Dashed lines are the model simulation from the parameter estimates.

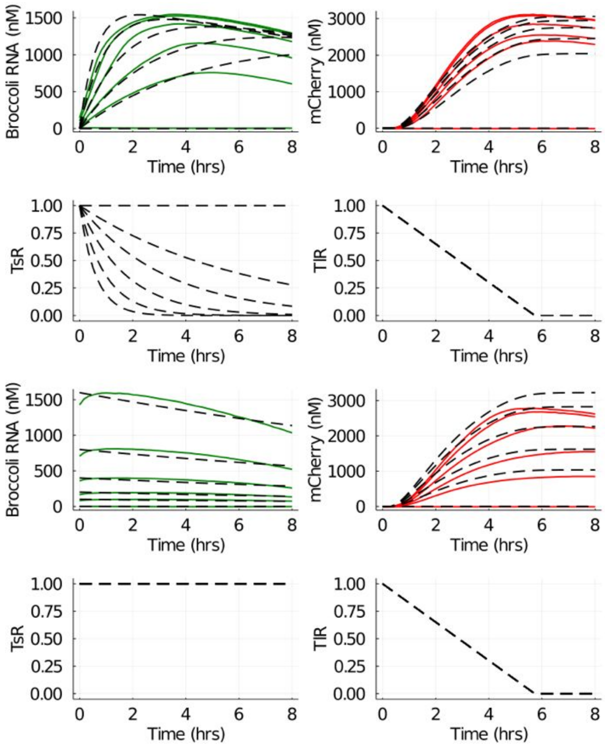

Figure S22. Model 5 fit with DNA titration and RNA titration bulk experiments. Colored lines are mean timeseries experimental data for the different species RNA, $Protein$, TsR, and TlR. Dashed lines are the model simulation from the parameter estimates.

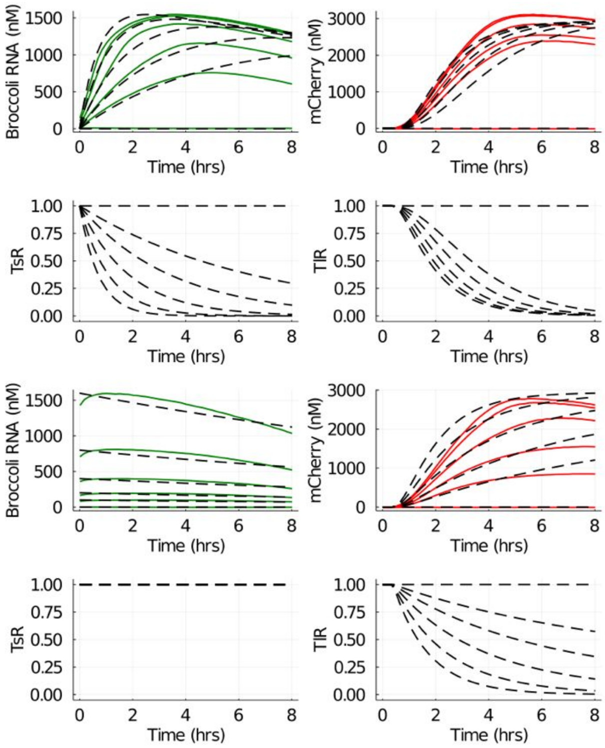

Figure S23. Model 6 fit with DNA titration and RNA titration bulk experiments. Colored lines are mean timeseries experimental data for the different species RNA, $Protein$, TsR, and TlR. Dashed lines are the model simulation from the parameter estimates.

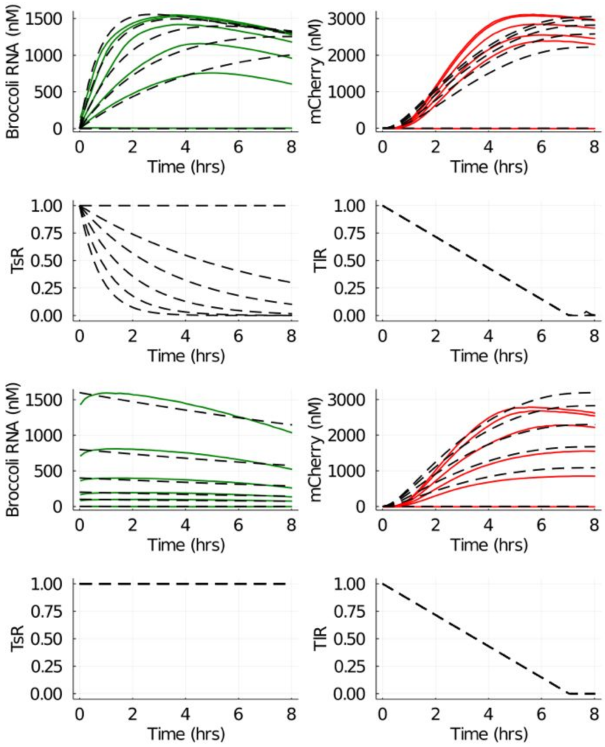

Figure S24. Model 7 fit with DNA titration and RNA titration bulk experiments. Colored lines are mean timeseries experimental data for the different species RNA, $Protein$, TsR, and TlR. Dashed lines are the model simulation from the parameter estimates.

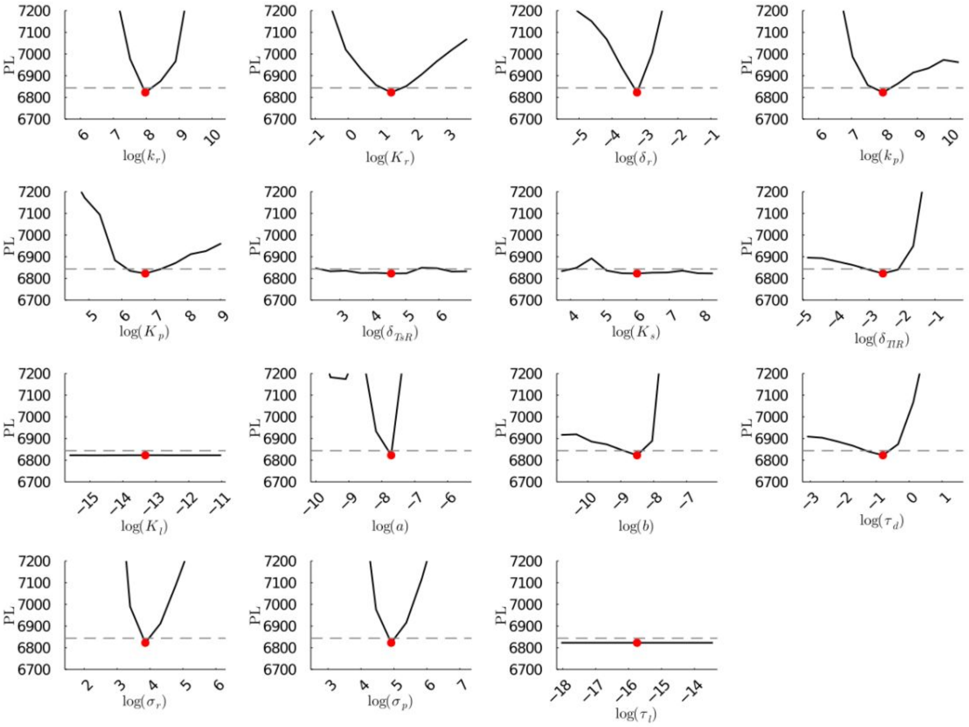

Figure S25. Profile likelihoods of parameters from model 1. Each plot corresponds to a parameter in the model and additional fitting parameters (σ_r, σ_p, and τ_d). The y-axis of each plot shows the minimized negative log-likelihood of the model with respect to all parameters except for the parameter given its value in the x-axis. The red dot shows the optimized parameter set with the negative MLE. The dashed grey line is the 95% significance threshold line. All logarithms are in natural log.

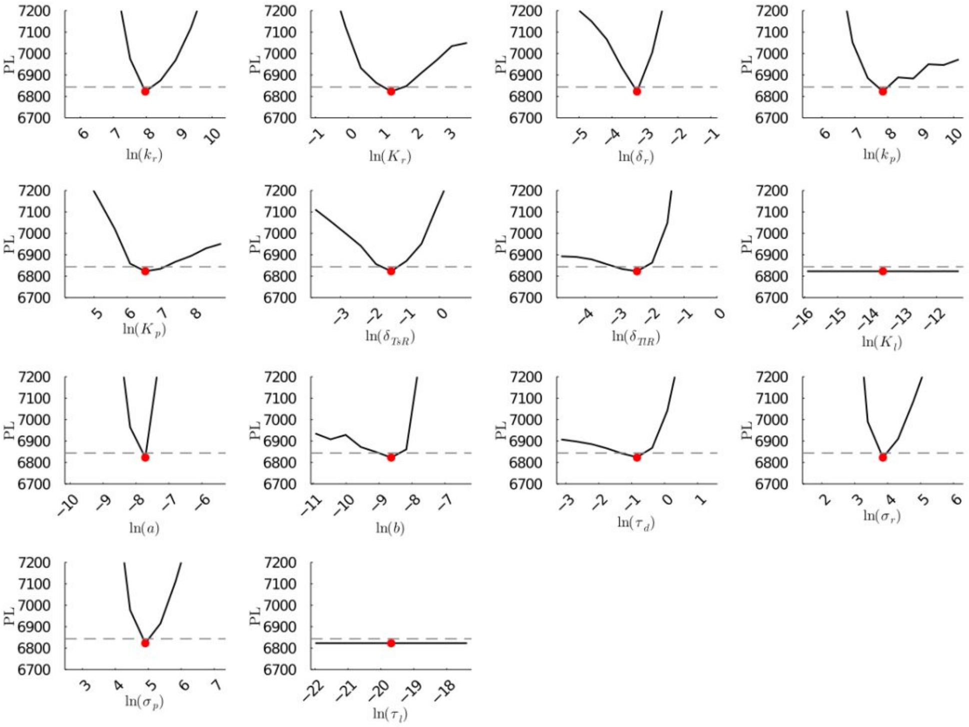

Figure S26. Profile likelihoods of parameters from model 2. Each plot corresponds to a parameter in the model and additional fitting parameters (σ_r, σ_p, and τ_d). The y-axis of each plot shows the minimized negative log-likelihood of the model with respect to all parameters except for the parameter given its value in the x-axis. The red dot shows the optimized parameter set with the negative MLE. The dashed grey line is the 95% significance threshold line. All logarithms are in natural log.

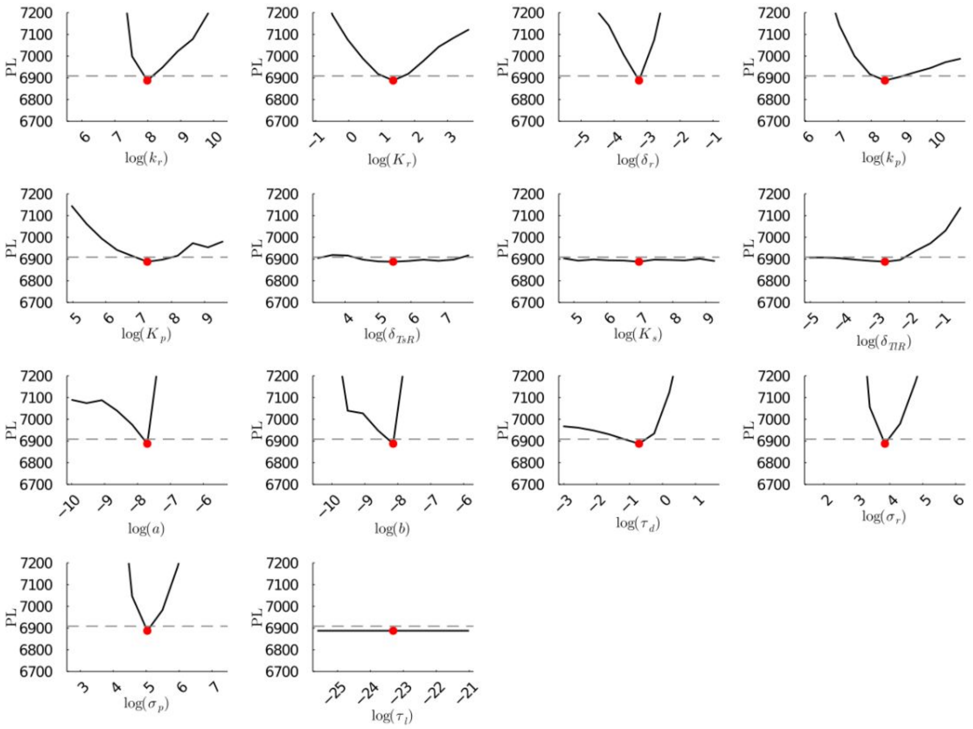

Figure S27. Profile likelihoods of parameters from model 3. Each plot corresponds to a parameter in the model and additional fitting parameters (σ_r and σ_p). The y-axis of each plot shows the minimized negative log-likelihood of the model with respect to all parameters except for the parameter given its value in the x-axis. The red dot shows the optimized parameter set with the negative MLE. The dashed grey line is the 95% significance threshold line. All logarithms are in natural log.

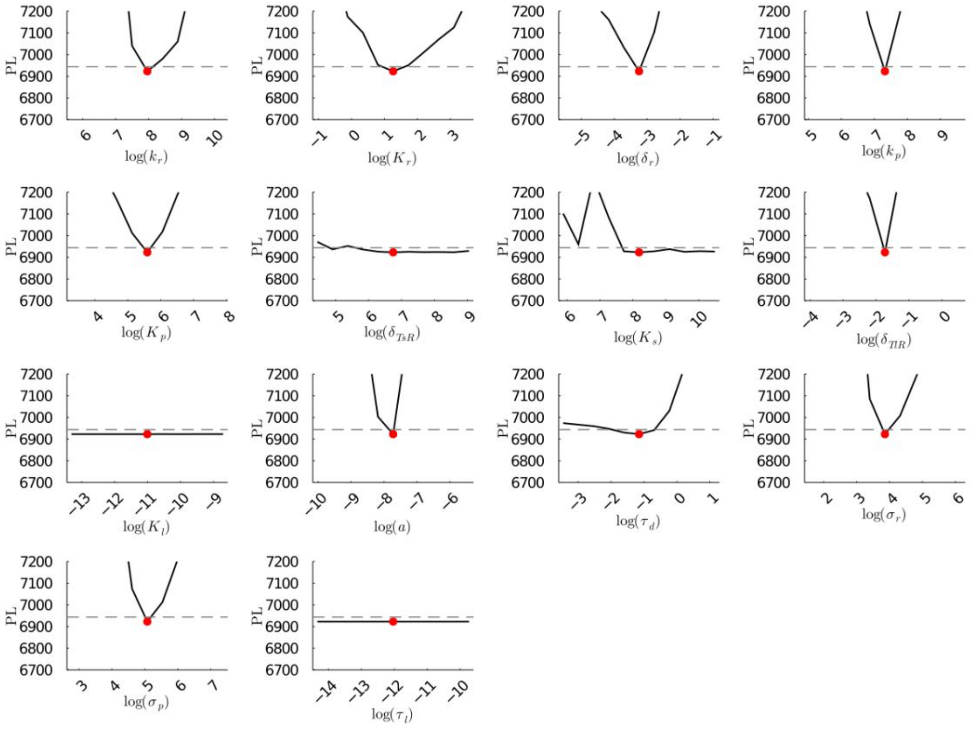

Figure S28. Profile likelihoods of parameters from model 4. Each plot corresponds to a parameter in the model and additional fitting parameters (σ_r and σ_p). The y-axis of each plot shows the minimized negative log-likelihood of the model with respect to all parameters except for the parameter given its value in the x-axis. The red dot shows the optimized parameter set with the negative MLE. The dashed grey line is the 95% significance threshold line. All logarithms are in natural log.

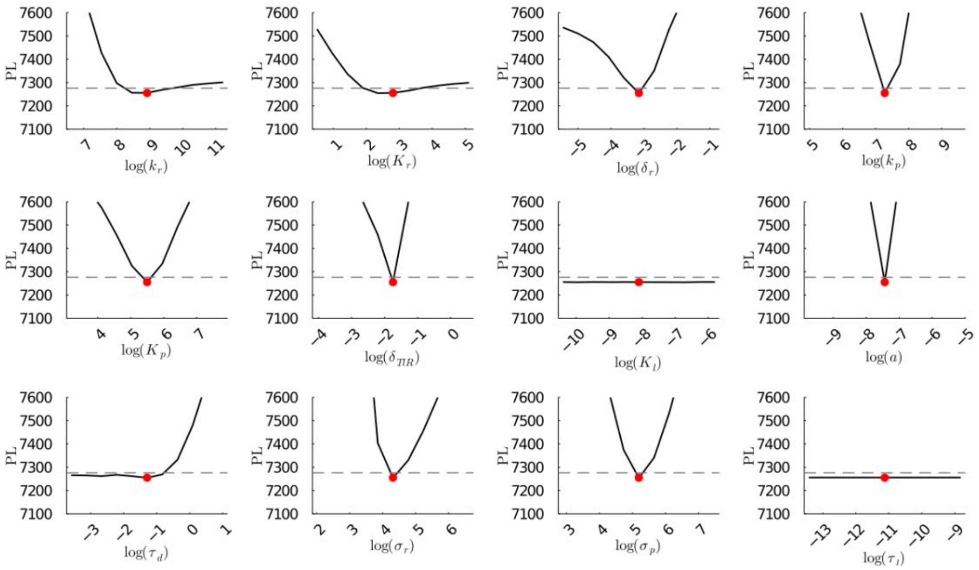

Figure S29. Profile likelihoods of parameters from model 5. Each plot corresponds to a parameter in the model and additional fitting parameters (σ_r and σ_p). The y-axis of each plot shows the minimized negative log-likelihood of the model with respect to all parameters except for the parameter given its value in the x-axis. The red dot shows the optimized parameter set with the negative MLE. The dashed grey line is the 95% significance threshold line. All logarithms are in natural log.

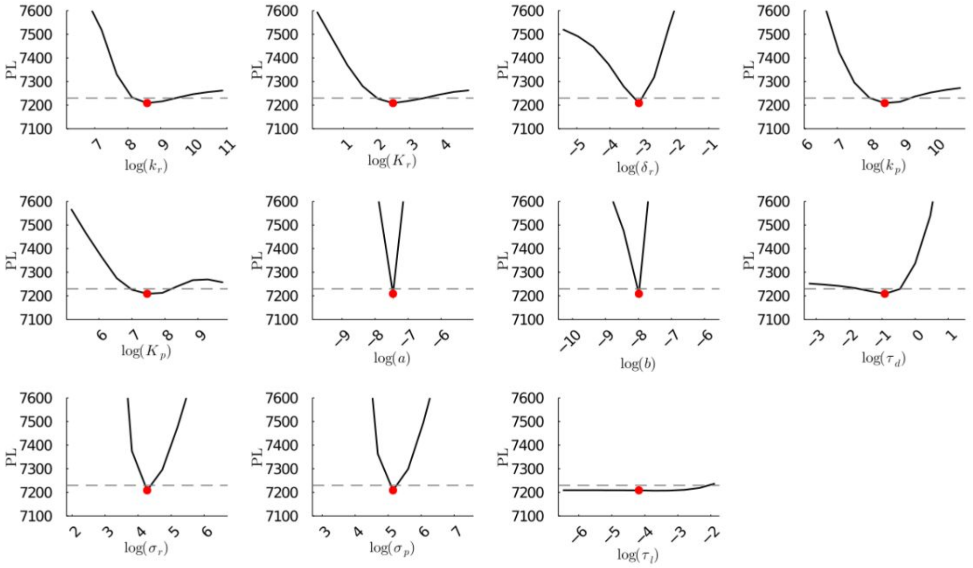

Figure S30. Profile likelihoods of parameters from model 6. Each plot corresponds to a parameter in the model and additional fitting parameters (σ_r and σ_p). The y-axis of each plot shows the minimized negative log-likelihood of the model with respect to all parameters except for the parameter given its value in the x-axis. The red dot shows the optimized parameter set with the negative MLE. The dashed grey line is the 95% significance threshold line. All logarithms are in natural log.

10. Microfluidic chip fabrication and pretreatment

PDMS microfluidic chips were designed and fabricated as presented in [11] using soft-photolithography methods. Briefly, 4" Si-wafer master molds were prepared by spin coating (model no. WS-650MZ- 23NPPB, Laurell Tech. Corp.) SU8 2025 (Microchem Inc.) to a height of 50 µm. The SU8-coated Si-wafers were pre-baked, exposed with UV-light through a film mask with the requisite design (*see supplementary CAD file in* [11]) for 7 s (MicroLithography Services), and then post-baked. SU8 development was then done by gently washing the wafer in developer solution (Microchem Inc.) for 3 mins before hard-baking the Si-wafers for 30 min at 200 °C. Lastly, the prepared master molds were silanized overnight (50 µl of 1H,1H,2H,2H-perfluorodecyltrichlorosilane) in a desiccator. PDMS with curing agent (10:1 mix) was degassed for 30 mins and poured over the prepared master molds. The PDMS was cured for 3 hours at 90 °C. After curing, the PDMS was peeled from the mold and inlet holes were punched using a 1mm biopsy puncher (Kai Europe GmbH) and then bonded onto glass cover slips by air plasma treatment (Plasma Cleaner PDC-002-CE, Harrick Plasma) at 600 mbar for 1 minute. After plasma treatment, the microfluidic chips were kept on a hotplate at 60 °C for 2 hours. Prior to encapsulation, the microfluidic device was pretreated with a polyelectrolyte solution to provide a hydrophilic coating in the channel between the second junction and the outlet (Fig. S31). First, a 1:3 (v/v) mixture of 37% (w/v) HCl and 50% (w/v) H_2O_2 was flushed through from the outlet towards the outer solution inlet by vacuum for 5 minutes to oxidize the channel walls. After washing with water, a 5% (w/v) solution of positive polyelectrolyte poly(diallyldimethylammonium chloride) (PDADMAC, 1.04g/ml, 1.375 n20/D) was flushed through for 10 minutes. After another water wash, a 2% (w/v) solution of negative polyelectrolyte poly(4-styrenesulfonic acid) (PSS, MW ~75000) was flushed for 5 minutes to form a hydrophilic coating. This was followed by a final water wash. Pretreated microfluidic chips were used on the same day of treatment.

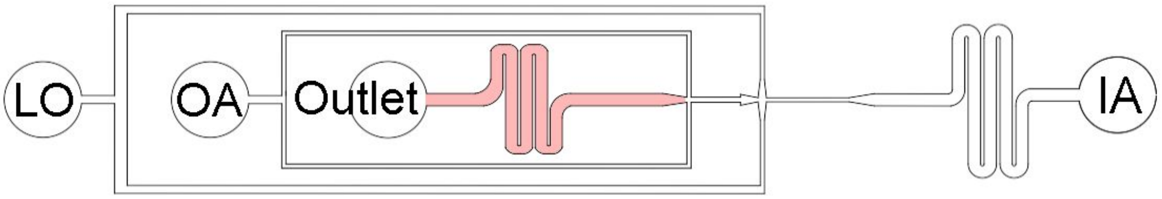

Figure S31. Pretreatment of the microfluidic chip was undertaken by flushing cleaning and coating solutions from the outlet towards the outer aqueous solution inlet (OA) (marked by red arrows) by vacuum. This provides a hydrophilic polyelectrolyte coating in the outlet channel (pink shaded area) to prevent the double-emulsions wetting on the channel walls. IA: inner aqueous solution inlet. LO: lipid oil phase inlet.

11. Encapsulation of CFES in liposomes using microfluidics

Before encapsulation, the osmolarity of the inner PURExpress CFES solution was measured using a freezing point osmometer (Osmomat 3000, Gonotec) calibrated with water, 300 mOsmol/kg, and 2000 mOsmol/kg NaCl solution standards. The outer aqueous solution was prepared according to Table S22 and then balanced to the same osmolarity of the inner CFES solution using water or a solution of 1.5 M glucose. DFHBI at a final concentration of 10 μM was also added into the outer solution for synthetic cells expressing the RNA Broccoli aptamer. The inner CFES solution was prepared according to Table S18. The lipid oil phase was composed of 1-Octanol with 6.5 mM of L-α-phosphatidylcholine (Egg PC) phospholipids, and 53.3 μM of 1,1'-Dioctadecyl-3,3,3',3'-Tetramethylindodicarbocyanine, 4-Chlorobenzenesulfonate Salt (DiD) fluorescent dye. To prepare the lipid oil phase, Egg PC (300 μL of 32.5 μM in chloroform) and DiD dye (80 μL of 1 mM in chloroform) were mixed together in a glass test tube, dried under flowing nitrogen gas for 5 minutes and dried under vacuum for 30 minutes. The dry lipid film was resuspended in 1.5 ml 1-octanol, and incubated at 37 °C in a sonication bath (Sonorex sonicator bath) for 1 hour. To generate synthetic cells, the inner CFES, lipid oil phase, and outer buffer solutions were dispensed through the pretreated microfluidic device (Section 10) using three pressure-regulated pumps (Dolomite Mitos P-Pump) at approximate pressures of 70:80:80 mbar, respectively. This resulted in a flow regime where the inner CFES solution was encapsulated into double-emulsions to form liposomes. Flow regimes and double-emulsion formation in the microfluidic device were monitored under brightfield using a Zeiss Andor Axiovert 200M with a 5x/0.15 Plan-Neofluar Ph1 M27 objective and PCO Dimax S4 Monochrome sCMOS high-speed camera. The cells were collected into a microcentrifuge tube, and then placed in channels made from parafilm channels sandwiched between a microscope slide (76x26x1 mm) and cover slip (24x60 mm). The ends of the channels were sealed with Picodent Twinsil Speed silicone to avoid evaporation (Fig. S32). Imaging was done on either a widefield or confocal microscope (Fig. S33).

Table S22. CFES feeding buffer (outer solution) recipe.

Component	Concentration
ATP *	6 mM
CTP *	4 mM
UTP *	4 mM
GTP *	6 mM
Amino acids**	0.5 mM
Spermidine	1.5 mM
DTT	1.5 mM
Folinic Acid	0.02 mM
K-Glu	280 mM
Mg-Glu	20 mM
HEPES	100 mM
Pluronic Acid	2%
KOH	17.65 mM
Glucose	480 mM
pH	7.6
Osmolarity	1441mOsmol/kg

*Stock NTP mix was prepared by combining NTPs, dissolving in water, and titrating with 15% KOH to pH 7.5.
**Stock amino acid mix was prepared with *each* amino acid at 0.5 mM at a final pH of 7.92.

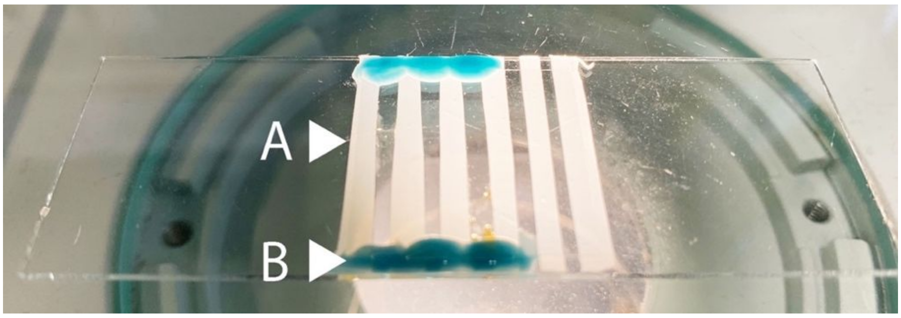

Figure S32. Glass slide setup for confocal microscopy synthetic cell populations. (A) Channels between parafilm strips where synthetic cells are loaded. (B) Twinsil Speed silicone sealing the ends of each channel.

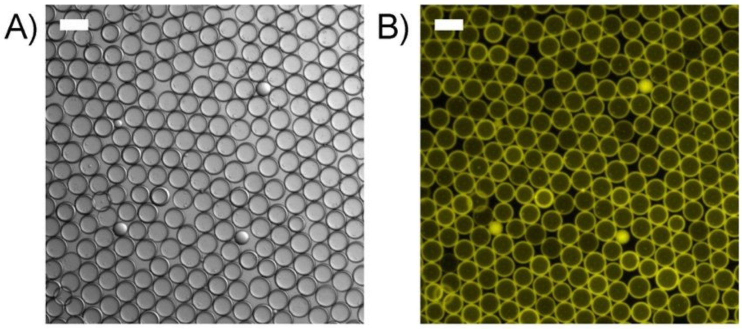

Figure S33. Example of a microfluidic-generated liposome population encapsulating PURExpress CFES. Lipids were composed of Egg PC with DiD dye. (A) Brightfield image and (B) fluorescence image of the same population using a Zeiss Andor Axiovert 200M widefield microscope with a 5x/0.15 Plan-Neofluar Ph1 M27 objective. Fluorescence excitation was at 550nm through a ROX filter set (excitation bandpass 575±15 nm, beam splitter HC BS 596 nm, emission BP 641±75 nm). White scale bars are 100 µm.

12. Inverse emulsion phase transfer method

Liposomes generated using the bulk inverse emulsion phase transfer method was prepared using a modified protocol as described in [12]. Briefly, 7.5 ul of PURExpress CFES with pEXP5-NT/eGFP plasmid DNA (Table S23) was added into a 1.5 ml microcentrifuge tube with 375ul of lipid oil phase consisting of 0.39 mM Egg PC and 1.25 µM Texas Red DHPE in mineral oil. To prepare the lipid oil phase, the POPC (36 µL of 32.5 µM in chloroform) and Texas Red DHPE (1µL of 1mM in chloroform) were mixed together in a glass test tube, dried under flowing nitrogen gas for 5 minutes and in vacuum for 30 minutes, resuspended in 2 mL mineral oil, and incubated at 37 °C in a sonication bath (Sonorex sonicator bath) for 1 hour. The mixture was then emulsified by running the tube across a microcentrifuge tube rack ten times. Then, 100 µl of the emulsion was gently layered on top of a prepared 100 µl outer feeding buffer solution (Table S24) + 40 µl lipid oil phase solution in BSA-coated wells of 96-well plate. The plate was centrifuged at 3000 xg for 10 minutes to produce liposomes that settle at the bottom of the plate. The plate was then incubated at 30 °C overnight (approx. 15 hours) and then imaged using confocal microscopy (LSM 880 with Airyscan, 40X/1.2 C-Apochromat objective). Excitation/detection wavelengths are 488 nm/505-515 nm for eGFP protein and 594 nm/605-615 nm for Texas Red DHPE (Fig. S34). Liposomes were segmented manually using Fiji 1.53c [13] and the size and expressed eGFP fluorescence distributions were collected using Python (v3.6) (*see Jupyter Notebooks in supplementary files*).

Table S23. PURExpress master mix for inverse emulsion

Component	Volume (µl)
Solution A	5
Solution B	3.75
RNAse Inhibitor	0.25
Sucrose (400 mM)	1
pEXP5-NT/eGFP (64 ng/ul)	0.5
Water	2
**TOTAL**	**12.5**

Table S24. CFES feeding buffer outer solution recipe for inverse emulsion phase transfer method.

Component	Concentration
ATP*	5 mM
CTP*	1.5 mM
UTP*	1.5 mM
GTP*	3 mM
Amino acids**	0.5 mM
Spermidine	1.5 mM
DTT	1.5 mM
Folinic Acid	0.02 mM
K-Glu	280 mM
Mg-Glu	20 mM
HEPES	150 mM
Glucose	200 mM
pH	7.35
Osmolarity	1339 mOsmol/kg

*Stock NTP mix was prepared by combining NTPs, dissolving in water, and titrating with 15% KOH to pH 7.5.
**Stock amino acid mix was prepared according to Table S21. The concentration of *each* amino acid is 0.5 mM.

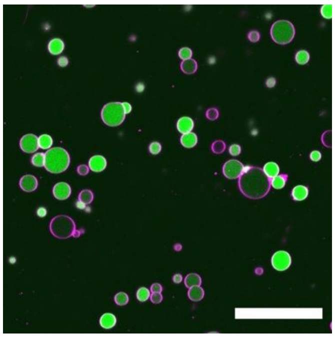

Figure S34. Confocal image composite of inverse emulsion phase transfer generated liposomes encapsulating PURExpress CFES. Liposomes are labeled with Texas Red DHPE in the lipid channel (magenta) and expressed eGFP protein (green) after ~15 hours incubation at 30 °C. Scale bar is at 100 µm.

13. Image acquisition and analysis

Here, we briefly describe the steps for image acquisition and analysis of calibration, timeseries, and z-stack confocal microscopy data of microfluidic-generated synthetic cell populations.

Image acquisition
Confocal images of cell free expression encapsulated within lipid vesicles were acquired using an LSM 880 Airyscan inverted laser scanning confocal microscope with a 10X/0.45 Plan-Apochromat M27 objective. The same acquisition settings were used for all microfluidic-generated synthetic cell population experiments. For timelapse imaging, the laser was focused at the equator of the synthetic cells and then imaged at the following excitation/emission wavelengths are 488nm/499-561 nm for RNA Broccoli and eGFP protein, 561 nm/579-641 nm for mCherry protein, and 633 nm/640-720 nm for DiD lipid dye every 5 minutes for a total of 12 hours. Z-stack images of the samples were taken at the endpoint focused at the top and bottom of the synthetic cells and then divided into 20 intervals.

Software and code
Image analysis was undertaken in Python (v3.6) using the following packages: Numpy (v1.19.1), Pandas (v1.1.3), Scipy (v1.5.2), Matplotlib (v3.3.1), Pystackreg (v0.2.2) [14], Scikit-image (v0.16.2) [15], and H5py (v3.1.0).

Calibration
Protein and mRNA standard solutions were prepared on glass slides and imaged under the same experiment confocal microscopy acquisition settings for calibration. A mask of 10 μm diameter circles across each image was used to sample each image and obtain fluorescence measurements for the confocal calibration curve (Fig. S35).

Image intensity correction (planar shading correction)
We observed an uneven and tilted illumination of the samples across the images. Low magnification objectives, such as the 20x objective which we used for our experiments, are sensitive to tilting errors because of their large field of views. Tilting was accounted for by using a planar shading correction [16]. This was done by fitting the single cell fluorescence data points or regions of interest (ROIs) in their respective coordinates to an equation of a plane and then tilting the plane back to a flat horizontal fixed at the origin. This method of correction was applied individually to all confocal images used in this study including the confocal images for calibration. To illustrate, Figs. S36 shows the uneven illumination in an image and the method of correction. Using the grid mask (Fig. S35) for bulk solution images or cell masks (e.g. Fig. S40) for synthetic cell population images, RFU values of the ROIs were plot along the x and y-axes and then corrected to have an average RFU values to be constant across the plane (Fig. S37).

Analysis of Timelapse images
Image stacks from timeseries confocal microscopy experiments were first registered to align the stack of each channel to its first image (Fig. S38). The last registered image on the lipid channel stack was then used as a reference image to segment cells of a synthetic cell population throughout the timeseries. A gaussian filter was used to smooth the reference image before thresholding and filling in small holes. The binary image was then labelled for each individual object and filtered by size and circularity to get only the synthetic cells. Cells with defects (*e.g.* oil drops or shadows from debris) were manually checked and removed. Cells near the edge of the image frame were also avoided (Fig. S39) to ensure only complete vesicles were analyzed. Fluorescence measurements of each labeled synthetic cell were collected from a 10 μm diameter circle in the center of each cell (Fig. S40). Single cell fluorescence timeseries measurements for each cell population were saved in csv files.

Z-stack images
A maximum projection of the lipid channel along the z-axis from z-stack endpoint images of the timeseries confocal microscopy experiments were used as the reference image for cell segmentation. The same workflow of gaussian filter > thresholding > filling > labelling > manual cleaning > filtering by circularity and size was then done on the reference image to segment the synthetic cells (Fig. S41). Cell sizes were obtained from the equivalent diameters of the segmented cells. Fluorescence measurements of each labeled synthetic cell was collected from the maximum value along the z-axis of a 10 μm diameter circle in the center of each cell. Single cell fluorescence measurements and sizes for each cell population were saved in csv files.

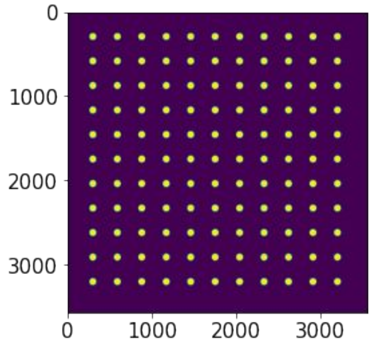

Figure S35. Example mask of 10 µm diameter circles in a grid used to collect fluorescence measurements in standard calibration images.

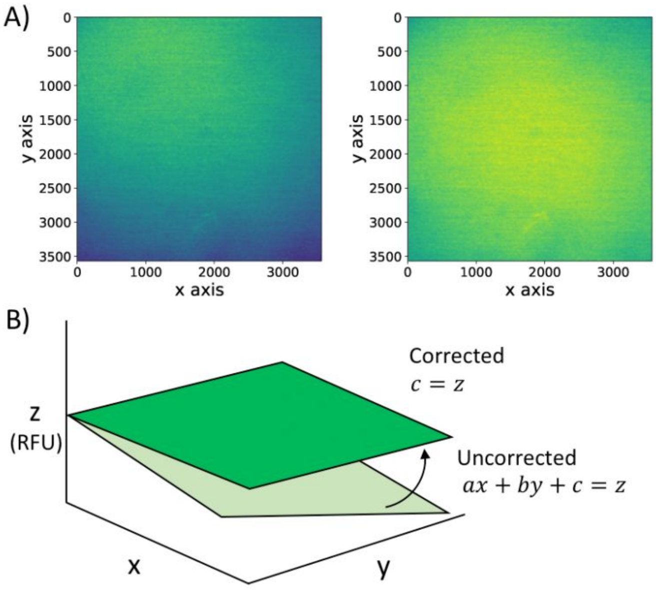

Figure S36. Illustration of image analysis correction for uneven and tilted illumination. (A) Example of confocal image of a bulk sample of eGFP protein with uneven and tilted illumination before (left) and after (right) planar shading correction. Images are gaussian filtered to more distinctly show gradients. (B) Fluorescence values of single cells across the plane follow a gradient due to uneven and tilted illumination. This is corrected by enforcing the average fluorescence signal to be constant across the plane. To obtain the corrected data, fluorescence values were subtracted by $(ax + by)$ from the plane fit of uncorrected data.

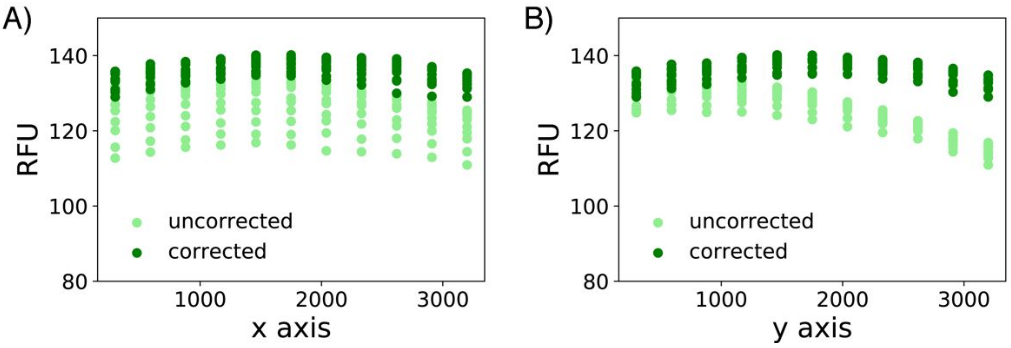

Figure S37. Example dataset from a confocal image with respect to the (A) x and (B) y-axes before and after correction from uneven illumination.

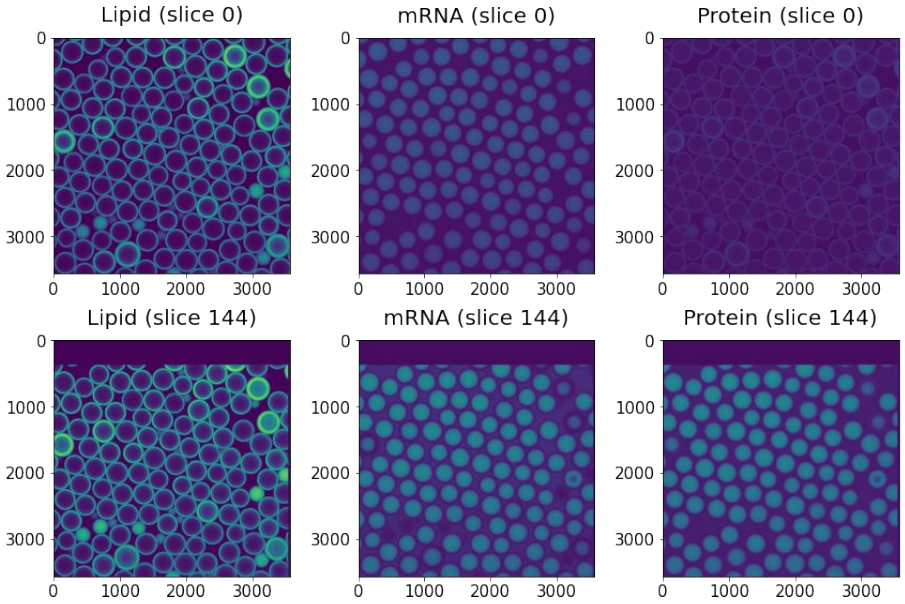

Figure S38. Example image registration of timeseries stacks. The three channels (lipid, mRNA, and protein) for the first (slice 0) and last (slice 144) image in the timeseries after registration using Pystackreg. X and Y axes are given in pixel units.

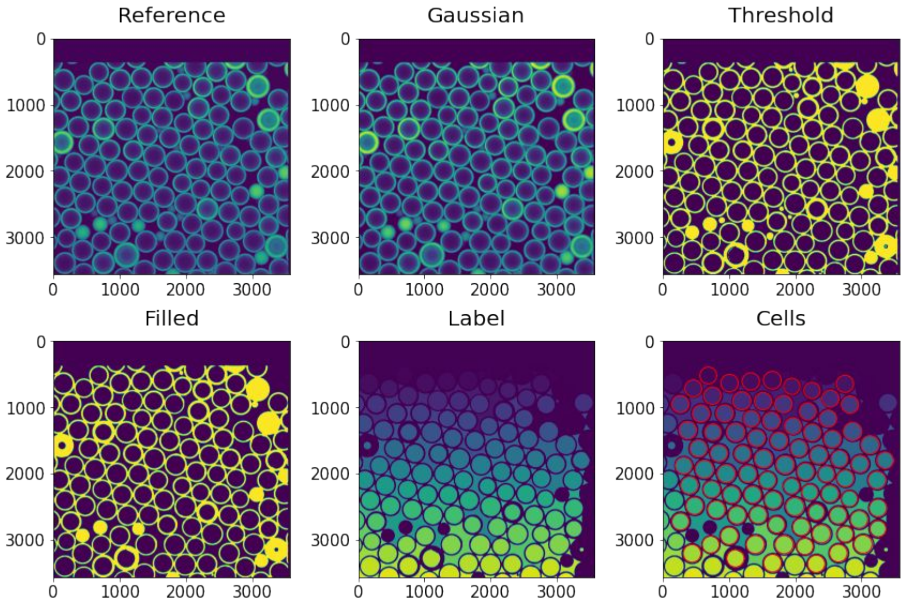

Figure S39. Example image segmentation of a reference image in the lipid channel. From left to right: reference lipid image, gaussian filter, convert to binary by thresholding, filling in small holes, labelling objects, and filtering cells by shape and size (labeled in red circles).

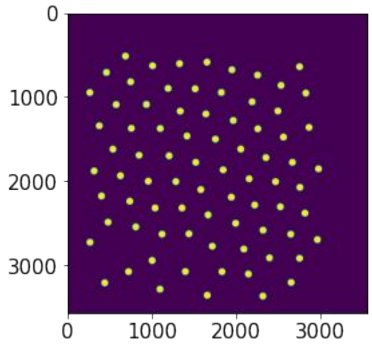

Figure S40. Example mask of 10 µm diameter circles from the cell segmentation result in Fig. S39.

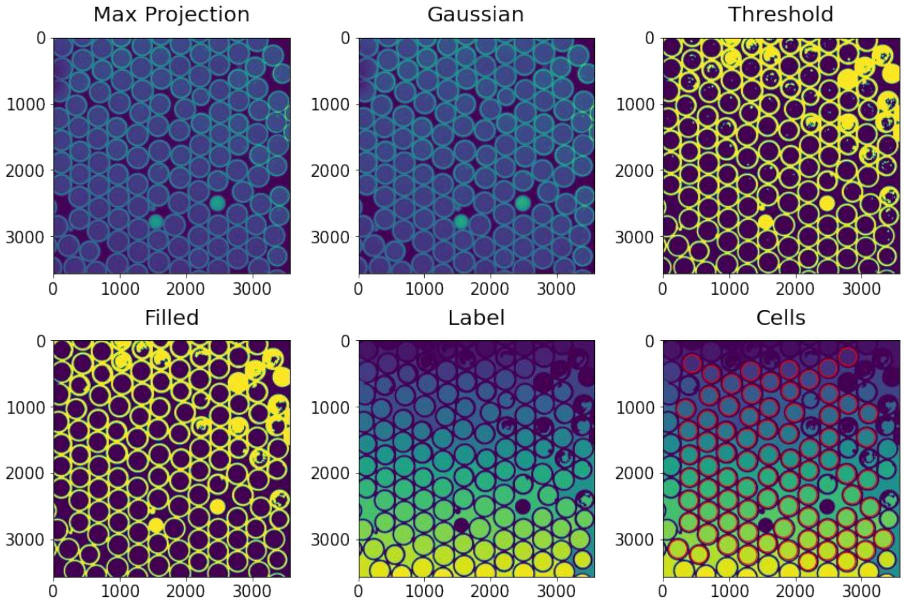

Figure S41. Example image segmentation of a z-stack. From left to right: maximum projection along z-axis, gaussian filter, convert to binary by thresholding, filling in small holes, labelling objects, and filtering cells by shape and size (labeled in red circles).

14. Gene expression dynamics in synthetic cell populations

Synthetic cell populations with varying DNA plasmid concentrations were prepared using a PURExpress CFES inner solution and feeding outer buffer solution. The lipid oil phase was composed of 1-Octanol with 6.5mM Egg PC, and 53.3 µM DiD dye. Inner solutions were prepared according to Table S18 with different pEXP5-NT/6xHis mCherry F30-2xdBroccoli plasmid DNA concentrations (1.75, 3.5, and 7.0 nM). Outer solution was prepared according to Table S25. Osmolarities of the inner and outer solutions were 1330 mOsmol/kg and 1334 mOsmol/kg, respectively. The inner solutions were encapsulated into liposomes using double-emulsion microfluidics (Section 11). The synthetic cell populations were then loaded into channels of the prepared microscope slides for confocal timelapse imaging at 30 °C incubation for 12 hours (Figs. S42-44). Sample preparation typically took approximately 0.5-1 hours after adding the plasmid DNA in the PURExpress CFES reaction mix. The CFES was kept on ice during sample preparation until microfluidic encapsulation. For this reason, the initial delay of protein signal is not as evident in the synthetic cell populations as compared to the bulk CFES reactions. Histograms of endpoint expression from z-stack confocal images of the synthetic cell populations are shown in Fig. S47. To demonstrate the reproducibility of the method, endpoint measurements of synthetic cell population experiments from different batches with different DNA concentrations are collated in Fig. S48A. In the case of synthetic cell populations at 3.5 nM pEXP5-NT/6xHis mCherry F30-2xdBroccoli plasmid DNA (boxed in red), a total of 4 different batches results in a batch mean and standard deviation of 1745.4 nM ± 183.1 nM (CV 0.10). Gene expression dynamics at this DNA concentration showed batch-wise reproducibility with maximum protein expression rates at 472.6 ± 85.3 nM/hr (CV 0.16) (Fig. S48B). Timeseries data of the population mean for each DNA concentration in the synthetic cell populations were used for parameter estimation, profile likelihood calculations (Fig. S49), and confidence intervals (Table S26).

Table S25. Feeding outer solution for DNA titration in synthetic cell populations.

Component	Volume (µl)
Stock feeding solution (Table S22)	1000
DFHBI (4mM)	2.5
Water	88
**TOTAL**	**1090.5**

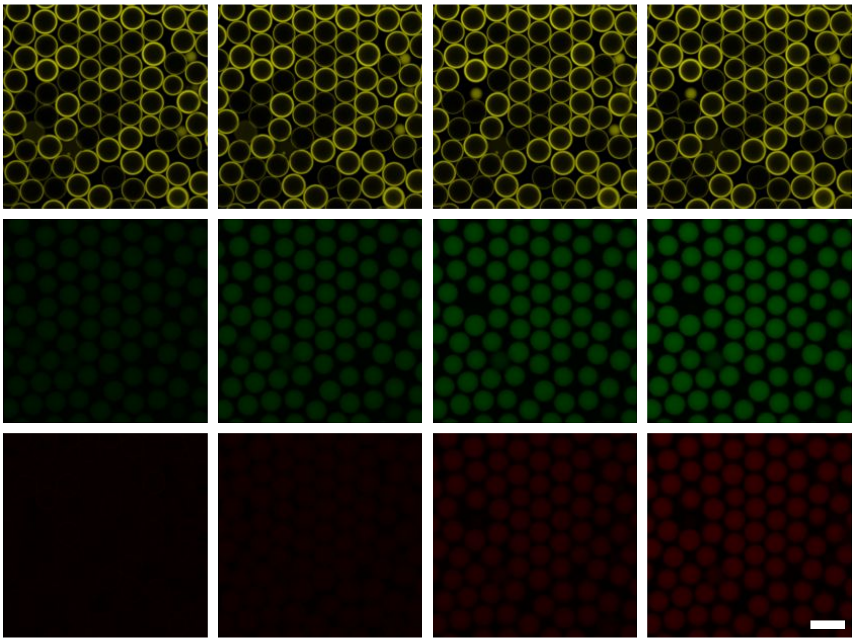

Figure S42. Timeseries images of synthetic cell population with 1.75 nM pEXP5-NT/6xHis mCherry F30 2xd-Broccoli plasmid DNA. Top row (yellow) shows the lipid channel, middle row (green) shows the RNA Broccoli channel, and bottom row (red) mCherry protein channel. Images are at 0, 1, 2, and 3-hour timepoints. Scale bar is 100 µm.

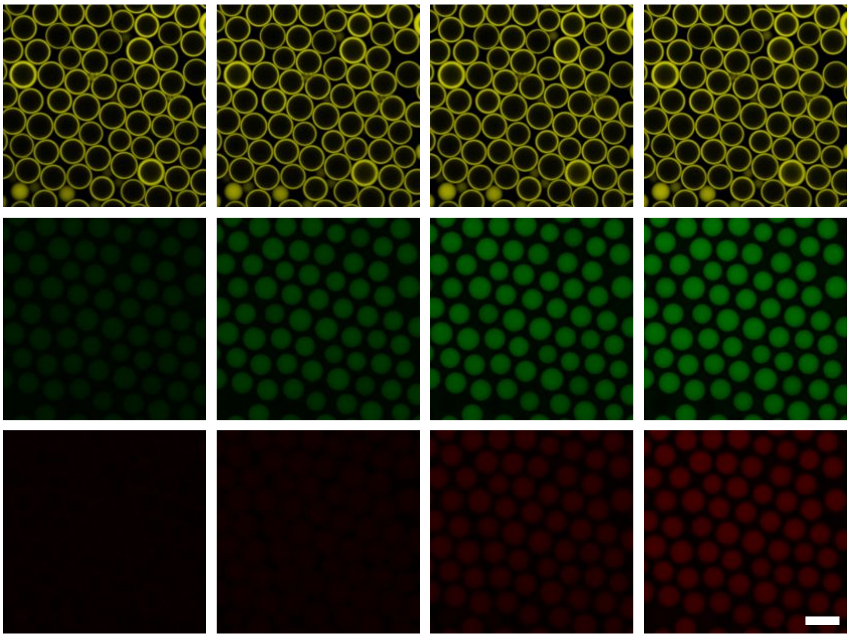

Figure S43. Timeseries images of synthetic cell population with 3.5 nM pEXP5-NT/6xHis mCherry F30 2xd-Broccoli plasmid DNA. Top row (yellow) shows the lipid channel, middle row (green) shows the RNA Broccoli channel, and bottom row (red) mCherry protein channel. Images are at 0, 1, 2, and 3-hour timepoints. Scale bar is 100 µm.

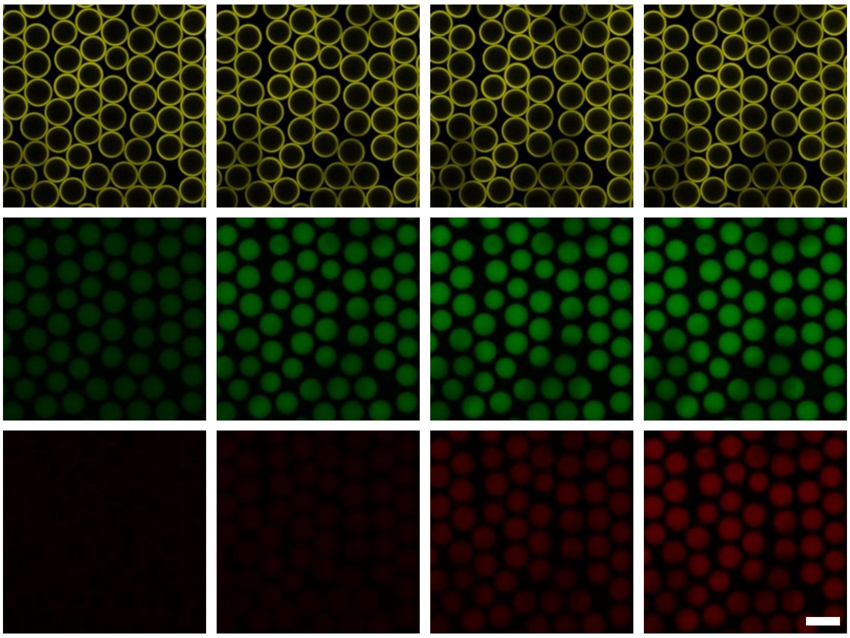

Figure S44. Timeseries images of synthetic cell population with 7 nM pEXP5-NT/6xHis mCherry F30 2xd-Broccoli plasmid DNA. Top row (yellow) shows the lipid channel, middle row (green) shows the RNA Broccoli channel, and bottom row (red) mCherry protein channel. Images are at 0, 1, 2, and 3-hour timepoints. Scale bar is at 100 µm.

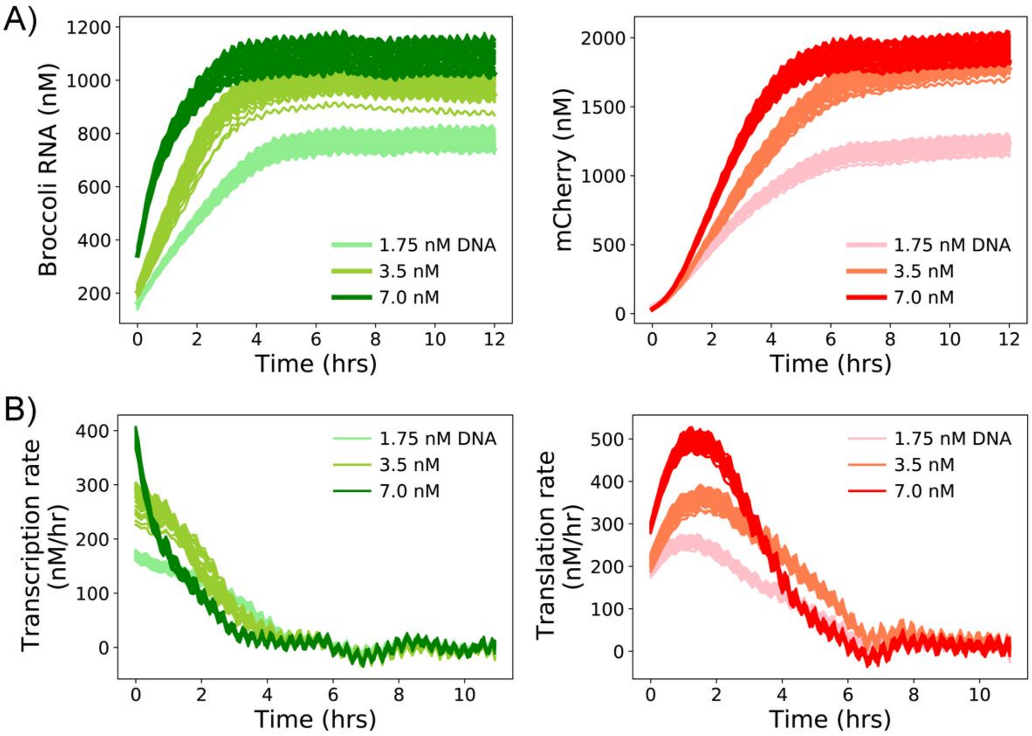

Figure S45. Timeseries of synthetic cell populations with varying DNA concentrations. (A) RNA Broccoli (green) and mCherry protein (red) concentrations over time for 1.75, 3, and 7 nM pEXP5-NT/6xHis mCherry F30 2xd-Broccoli plasmid DNA. (B) Rates of transcription and translation calculated from (A) using a rolling window average of 1 hour.

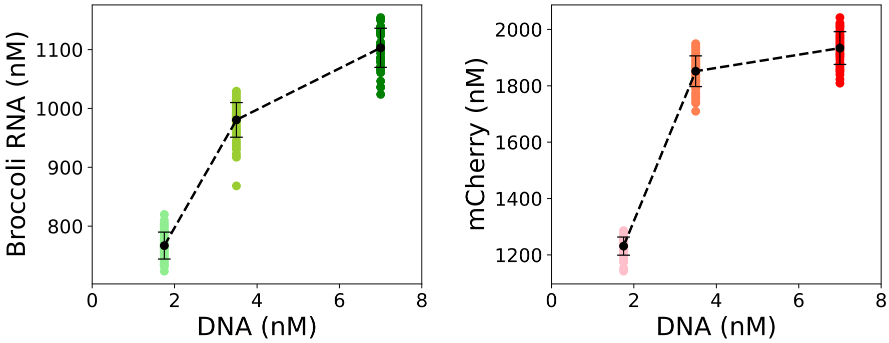

Figure S46. Endpoint RNA (green) and protein (red) concentrations of synthetic cell populations at varying DNA concentrations. Each dot is a single synthetic cell from endpoint. Black dots with error bars are mean and standard deviation of the populations.

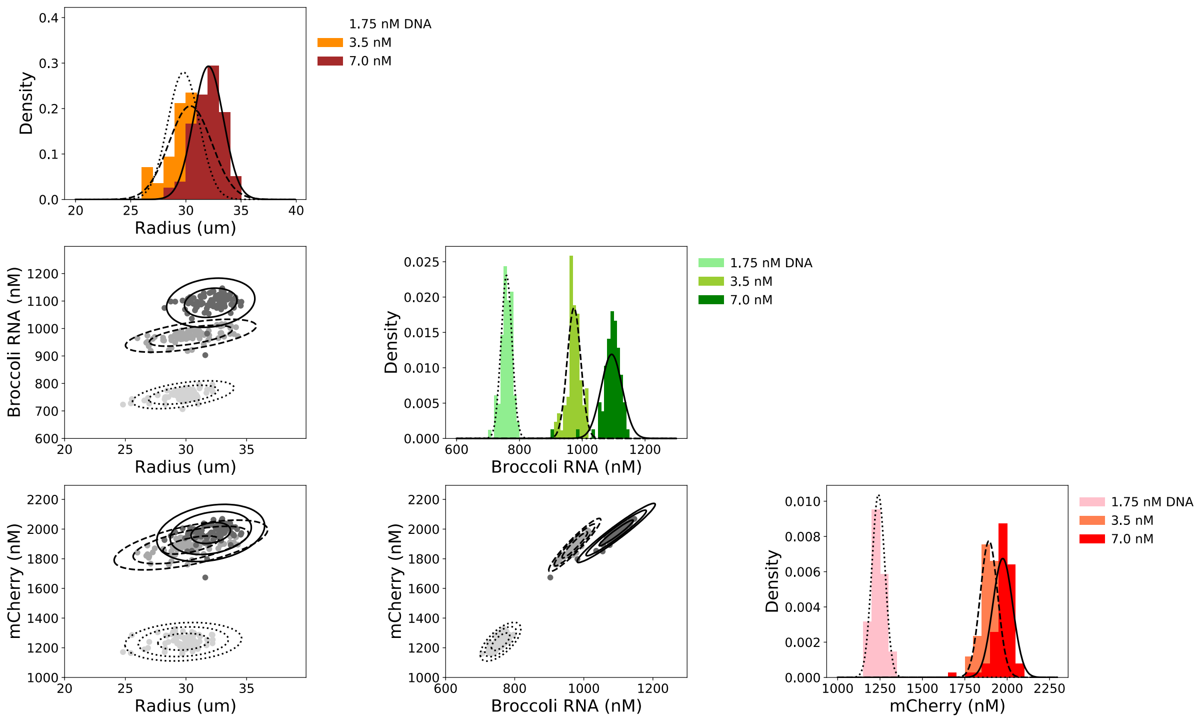

Figure S47. Distributions and correlations of radius, RNA, and protein concentrations in synthetic cell populations. Data is obtained from maximum values of z-stack images at endpoint after 30 °C incubation for 12 hours. Histograms are fit to univariate normal distributions and correlations are fit to bivariate normal distributions. Total number of cells analyzed are 82, 85, and 78 for populations 1, 2, and 3, respectively.

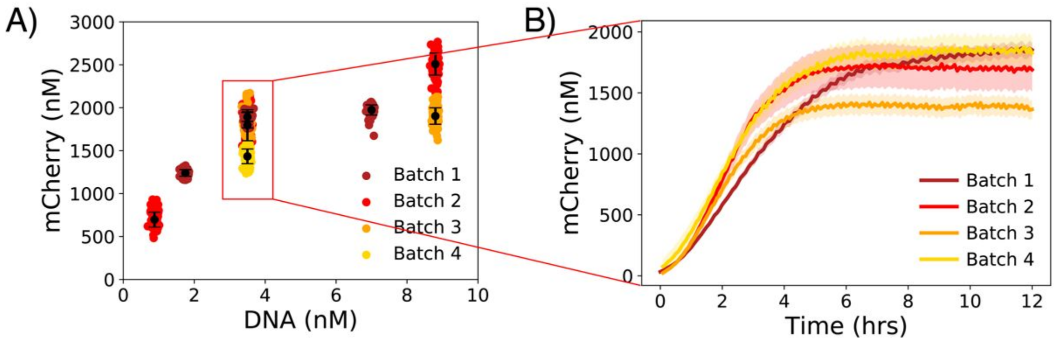

Figure S48. Batch variability of the synthetic cell populations. (A) Endpoint concentrations of synthetic cell populations at varying pEXP5-NT/6xHis mCherry F30 2xd-Broccoli plasmid DNA concentrations from different batches. Batches are plotted in different colors (each dot is a single synthetic cell) and means the experiment was performed on a different day with a new set of inner CFES, feeding outer buffer, and lipid phase solutions. Black dots with error bars are mean and standard deviation of the populations. (B) Dynamics of the different batches of synthetic cell populations with 3.5 nM pEXP5-NT/6xHis mCherry F30 2xd-Broccoli plasmid DNA.

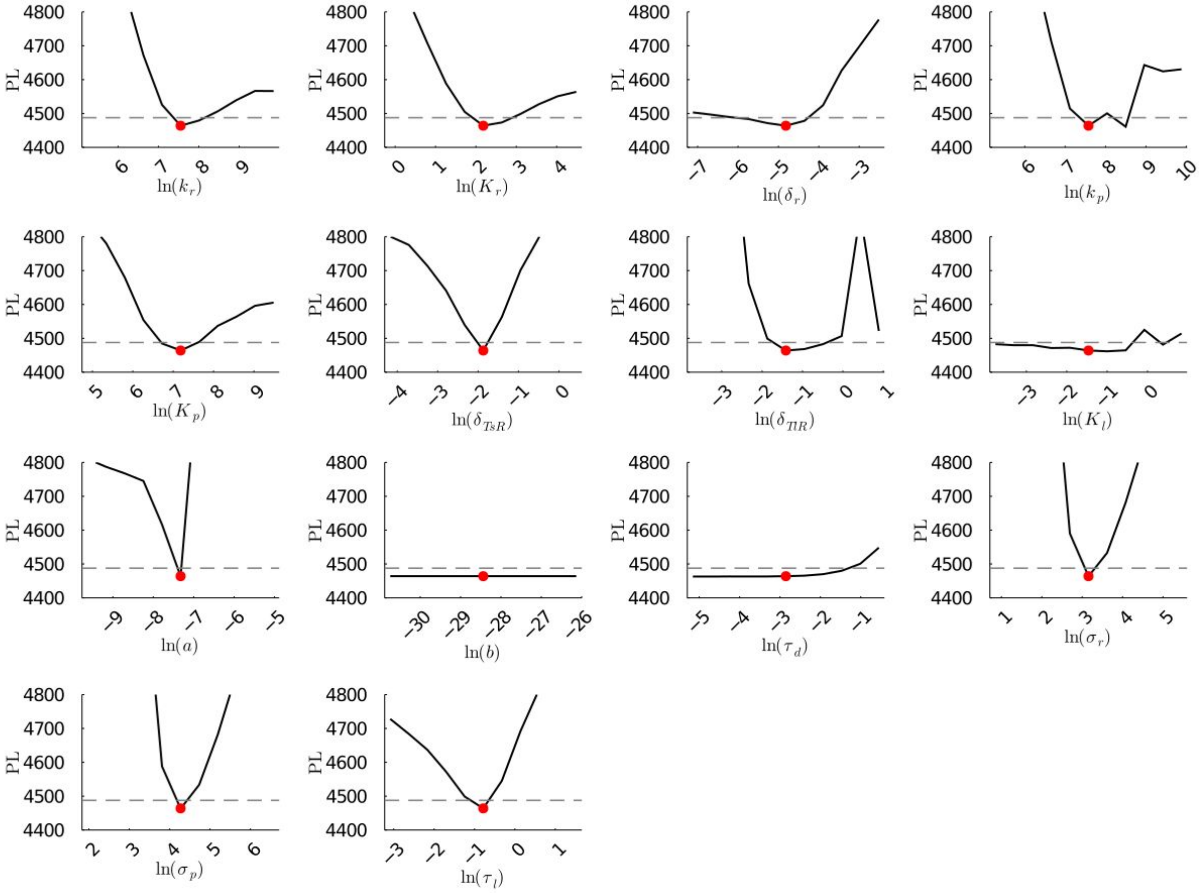

Figure S49. Profile likelihoods of parameters from model 2 using synthetic population experiment data. Each plot corresponds to a parameter in the model and additional fitting parameters (σ_r, σ_p, and τ_d). The y-axis of each plot shows the minimized negative log-likelihood of the model with respect to all parameters except for the parameter given its value in the x-axis. The red dot shows the optimized parameter set with the minimum negative log likelihood. The dashed grey line is the 95% significance threshold line. The intersections of the significance threshold line and profile likelihood are the likelihood-based confidence intervals of the optimized parameter.

Table S26. Parameter estimates and 95% likelihood-based confidence intervals of model 2 using synthetic cell population data.

Parameter	$\hat{\theta}$	95% CI	
k_r	1899	1631	3537
K_r	8.86	6.98	18.66
δ_r	0.0081	0.00239	0.0143
k_p	1954	1617	2696
K_p	1319	819	2038
k_{mat}	2.15	(±0.12)	
δ_{TsR}	0.154	0.136	0.175
δ_{TlR}	0.244	0.184	0.684
K_l	0.232	$-\infty$	0.713
a	6.60E-4	6.21E-4	6.74E-4
b	4.46E-13	$-\infty$	$+\infty$
τ_d	0.0576	$-\infty$	0.279
σ_r	23.48	21.83	28.25
σ_p	71.58	66.40	86.09
τ_l	0.457	0.342	0.535

15. Two plasmids in a synthetic cell population

By encapsulating two plasmids, pEXP5-NT/6xHis mCherry and pEXP5-NT/6xHis eGFP, a synthetic cell population can express both mCherry and eGFP protein. Inner and outer solutions were made according to Tables S27-28 and then encapsulated into liposomes using double-emulsion microfluidics. The lipid oil phase was composed of 1-Octanol with 6.5 mM Egg PC, and 53.3 µM DiD dye. The synthetic cell populations were then loaded into channels of the prepared microscope slides for confocal timelapse imaging at 30 °C incubation for 12 hours (Figs. S50-51). Z-stack confocal images of the synthetic cell populations were acquired at endpoint (Fig. S52).

Table S27. PURExpress inner solution for DNA titration in synthetic cell populations.

Component	eGFP & mCherry (µl)	eGFP only (µl)	mCherry only (µl)
Solution A	50	50	50
Solution B	37.5	37.5	37.5
RNAse Inhibitor	2.5	2.5	2.5
Sucrose (1.5 M)	6.67	6.67	6.67
pEXP5-NT/6xHis mCherry (533ng/µL)	2.35	0	2.35
pEXP5-NT/6xHis eGFP (467ng/µL)	2.68	2.68	0
Water	23.3	25.65	25.98
**TOTAL**	**125**	**125**	**125**
Osmolarity	1230 mOsmol/kg		

Table S28. Feeding outer solution for DNA titration in synthetic cell populations.

Component	Volume (µl)
Stock feeding solution (Table S22)	1000
Water	124
**TOTAL**	**1124**
Osmolarity	1230 mOsmol/kg

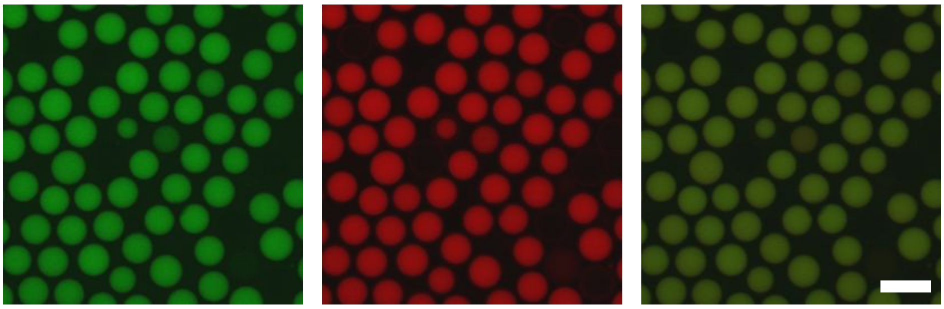

Figure S50. Endpoint confocal image of a cell population containing two plasmids pEXP5-NT/6xHis mCherry and pEXP5-NT/6xHis eGFP plasmids both at 10 ng/µL. Left panel (green) shows the eGFP protein channel, the middle panel mCherry protein channel, and the left panel is the merged image of eGFP and mCherry. Scale bar is at 100 µm.

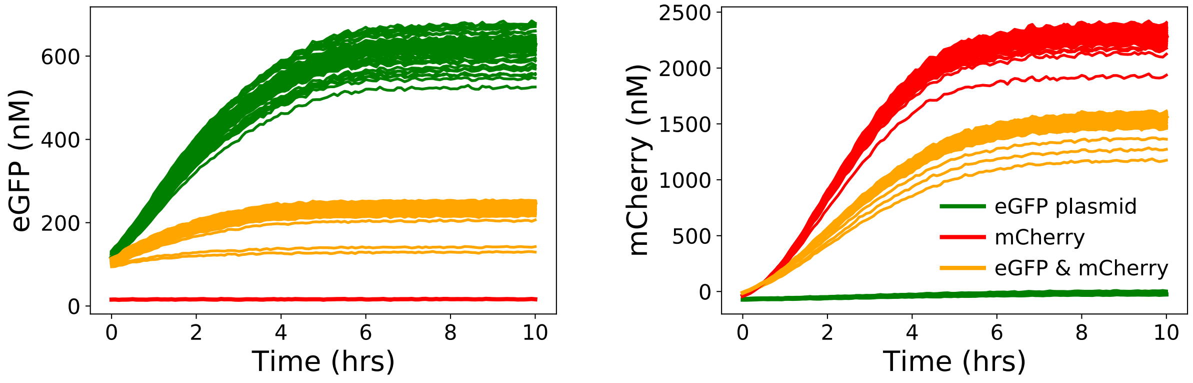

Figure S51. Timeseries of synthetic cell populations with pEXP5-NT/6xHis mCherry (red), pEXP5-NT/6xHis eGFP (green), or both pEXP5-NT/6xHis mCherry and pEXP5-NT/6xHis eGFP plasmids (yellow). Each plasmid is at a concentration of 10 ng/ul.

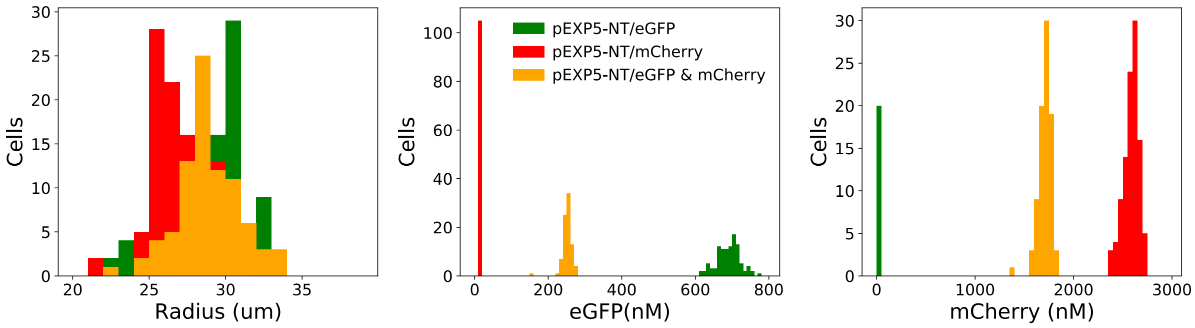

Figure S52. Histograms of endpoint radius, eGFP, and mCherry protein in the synthetic cell populations with pEXP5-NT/6xHis mCherry (red), pEXP5-NT/6xHis eGFP (green), or both pEXP5-NT/6xHis mCherry and pEXP5-NT/6xHis eGFP plasmids (yellow). Each plasmid is at a concentration of 10 ng/ul. Data is obtained from maximum values of z-stack images.

16. Dilution of CFES outer solution in synthetic cell populations

To determine if the outer solution conditions in microfluidic-generated synthetic cells significantly affect gene expression rates, synthetic cell populations were prepared with different dilutions of the outer solution composed of PURExpress CFES without DNA. An inner solution was prepared according to Table S18 with 3 nM of pEXP5-NT/6xHis mCherry F30-2xdBroccoli plasmid DNA (final osmolarity 1346 mOsmol/kg). The outer solution was also composed of PURExpress CFES, with glucose, pluronic acid, and no DNA (Table S29). To dilute the outer solution, a dilution mix composed of the same concentration of 10 µM DFHBI for the Broccoli aptamer, 2% pluronic acid to stabilize the synthetic cells, and glucose to maintain the same osmolarity of the outer solution (Table S30). Diluting the outer solution with the dilution mix then results in the dilution of all the buffer components except for DFHBI, pluronic acid, and the overall osmolarity. The lipid oil phase was composed of 1-Octanol with 6.5mM Egg PC, and 53.3µM DiD dye. The inner solution was encapsulated into liposomes by double-emulsion microfluidics. Aliquots of the collected liposomes were diluted with the dilution mix to result in 100%, 66%, 50%, 33%, 10% PURExpress CFES outer solutions. This was done by dropping a 10µL aliquot of the synthetic cells onto a microscope slide and then adding the appropriate volume of the dilution mix (0, 5, 10, 20, 100µL, respectively). The diluted liposomes were then gently mixed by pipetting and then loaded into channels of the prepared microscope slides for confocal timelapse imaging at 30 °C incubation for 12.5 hours. Despite the inner, outer, and dilution solutions being osmotically balanced, differences in composition between diluted outer solutions and the inner solutions resulted in material flux into the liposomes. This is because osmolarities are indirectly measured and balanced using freezing point depression measurements. As a result, comparing complex solutions of different compositions will deviate from the actual osmolarities [17]. This is observed in larger liposomes or bursting in the diluted samples. mRNA and protein expression were also negatively affected with more dilution of the PURExpress outer solution (Fig. S53-54).

Table S29. PURExpress outer solution.

Component	Volume (µl)
Solution A	100
Solution B	75
RNAse Inhibitor	5
DFHBI (500 µM)	5
10% Pluronic acid	50
Glucose (1.5 M)	13.3
Water	13.2
**TOTAL**	**261.5**
Osmolarity	1363 mOsmol/kg

Table S30. Dilution mix.

Component	Volume (µl)
Glucose (1.5 M)	815
DFHBI (4 mM)	3
10% Pluronic acid	240
Water	142
**TOTAL**	**1200**
Osmolarity	1351 mOsmol/kg

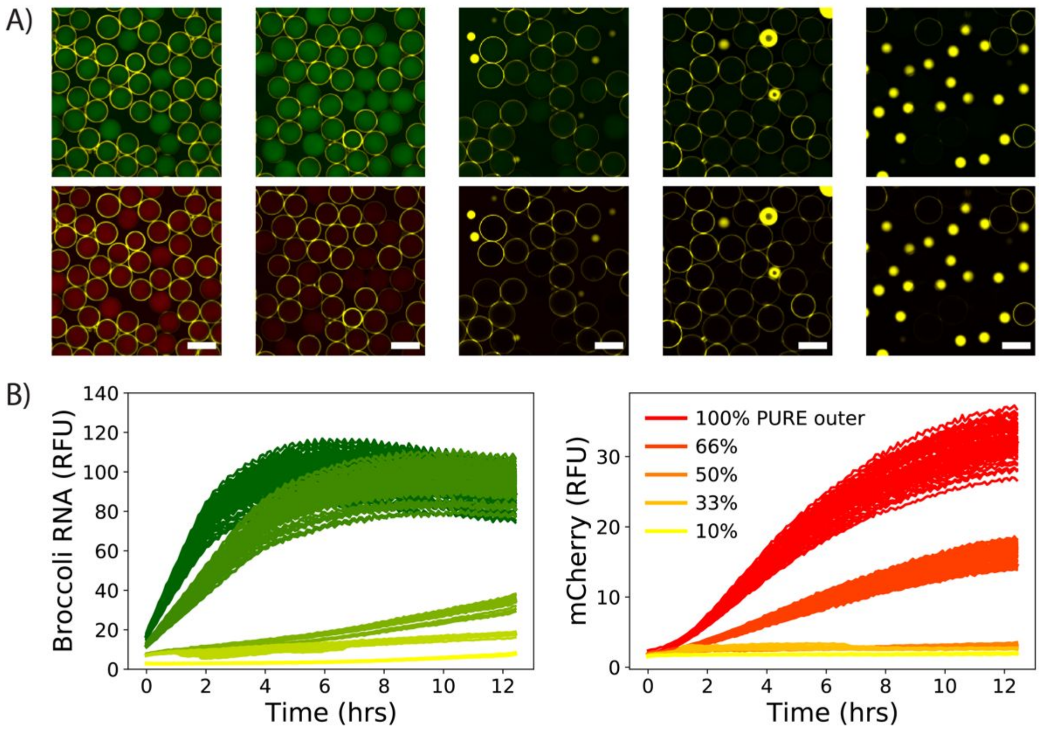

Figure S53. Dilutions of the outer solution in synthetic cell populations**.** (A) Confocal images of the synthetic cell populations with 3 nM of pEXP5-NT/6xHis mCherry F30-2xdBroccoli plasmid DNA in decreasing percentage of PURExpress CFES within the outer solution (100%, 66%, 50%, 33%, and 10% from left to right) at endpoint after 30°C incubation for 12.5 hours. Top row is for mRNA Broccoli channel (green) and bottom row is for mCherry protein (red) both overlaid with the lipid DiD dye channel (yellow). (B) Single-cell gene expression dynamics of expressed RNA and protein of the same synthetic cell populations. Total number of cells analyzed are 66, 69, 17, 16, and 17 for 100%, 66%, 50%, 33%, and 10% PURExpress outer solution populations, respectively. Scale bars are all 100 µm.

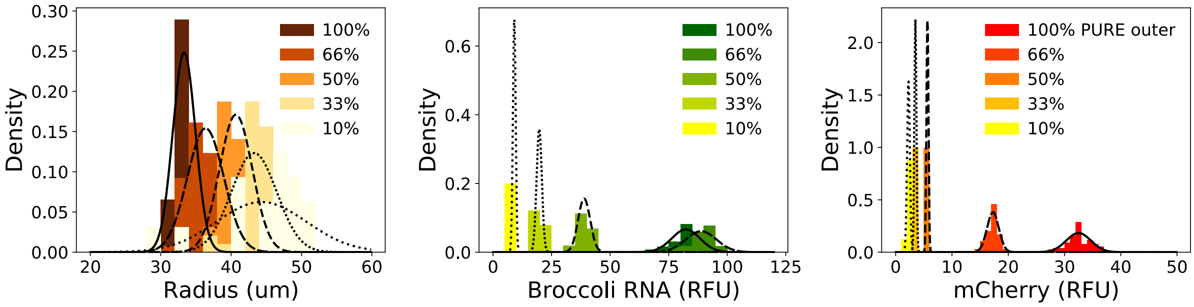

Figure S54. Endpoint histograms of radius, RNA Broccoli, and mCherry protein from confocal z-stack images of the synthetic cell populations at 100%, 66%, 50%, 33%, and 10% PURExpress outer solution after 12.5 hours of incubation at 30°C. Total number of cells analyzed are 76, 65, 32, 48, and 16 for 100%, 66%, 50%, 33%, and 10% PURExpress outer solution populations, respectively.

References

[1] Li M, Green DC, Anderson JLR, Binks BP, Mann S: **In vitro gene expression and enzyme catalysis in bio-inorganic protocells**. *Chem Sci* 2011, **2**:1739.

[2] Tang T-YD, van Swaay D, DeMello A, Ross Anderson JL, Mann S: **In vitro gene expression within membrane-free coacervate protocells**. *Chem Commun* 2015, **51**:11429–11432.

[3] Filonov GS, Moon JD, Svensen N, Jaffrey SR: **Broccoli: Rapid selection of an RNA mimic of green fluorescent protein by fluorescence-based selection and directed evolution**. *J Am Chem Soc* 2014, **136**:16299–16308.

[4] Filonov GS, Kam CW, Song W, Jaffrey SR: **In-gel imaging of RNA processing using broccoli reveals optimal aptamer expression strategies**. *Chem Biol* 2015, **22**:649–660.

[5] Gross LA, Baird GS, Hoffman RC, Baldridge KK, Tsien RY: **The structure of the chromophore within DsRed, a red fluorescent protein from coral**. *Proc Natl Acad Sci U S A* 2000, **97**:11990–11995.

[6] Garamella J, Marshall R, Rustad M, Noireaux V: **The All E. coli TX-TL Toolbox 2.0: A Platform for Cell-Free Synthetic Biology**. *ACS Synth Biol* 2016, **5**:344–355.

[7] Stögbauer T, Windhager L, Zimmer R, Rädler JO: **Experiment and mathematical modeling of gene expression dynamics in a cell-free system**. *Integr Biol* 2012, **4**:494.

[8] Bezanson J, Edelman A, Karpinski S, Shah VB: **Julia: A fresh approach to numerical computing**. *SIAM Rev* 2017, **59**:65–98.

[9] Raue A, Kreutz C, Maiwald T, Bachmann J, Schilling M, Klingmüller U, Timmer J: **Structural and practical identifiability analysis of partially observed dynamical models by exploiting the profile likelihood**. *Bioinformatics* 2009, **25**:1923–1929.

[10] Maiwald T, Hass H, Steiert B, Vanlier J, Engesser R, Raue A, Kipkeew F, Bock HH, Kaschek D, Kreutz C, et al.: **Driving the model to its limit: Profile likelihood based model reduction**. *PLoS One* 2016, **11**:1–18.

[11] Yandrapalli N, Petit J, Bäumchen O, Robinson T: **Surfactant-free production of biomimetic giant unilamellar vesicles using PDMS-based microfluidics**. *Commun Chem 2021 41* 2021, **4**:1–10.

[12] Moga A, Yandrapalli N, Dimova R, Robinson T: **Optimization of the inverted emulsion method for high-yield production of biomimetic giant unilamellar vesicles**. *ChemBioChem* 2019, doi:10.1002/cbic.201900529.

[13] Schindelin J, Arganda-Carreras I, Frise E, Kaynig V, Longair M, Pietzsch T, Preibisch S, Rueden C, Saalfeld S, Schmid B, et al.: **Fiji: An open-source platform for biological-image analysis**. *Nat Methods* 2012, **9**:676–682.

[14] Thévenaz P, Ruttimann UE, Unser M: **A pyramid approach to subpixel registration based on intensity**. *IEEE Trans Image Process* 1998, **7**:27–41.

[15] Van Der Walt S, Schönberger JL, Nunez-Iglesias J, Boulogne F, Warner JD, Yager N, Gouillart E, Yu T: **Scikit-image: Image processing in python**. *PeerJ* 2014, **2014**:1–18.

[16] Moëll MK, Donaldson LA: **Shading Correction Methods for Digital Image Analysis of Confocal Wood Images**. *IAWA J* 2007, **28**:349–364.

[17] Sweeney TE, Beuchat CA: **Limitations of methods of osmometry: measuring the osmolality of biological fluids**. *Am J Physiol Integr Comp Physiol* 1993, **264**:R469–R480.
